# In Silico Prediction of Novel Inhibitors of SARS-CoV-2 Main Protease through Structure-Based Virtual Screening and Molecular Dynamic Simulation

**DOI:** 10.3390/ph14090896

**Published:** 2021-09-03

**Authors:** Sobia Ahsan Halim, Muhammad Waqas, Ajmal Khan, Ahmed Al-Harrasi

**Affiliations:** 1 Natural and Medical Sciences Research Center, University of Nizwa, Nizwa 616, Oman; waqastakkar@gmail.com (M.W.); ajmalkhan@unizwa.edu.om (A.K.); 2 Department of Biotechnology and Genetic Engineering, Hazara University Mansehra, Dhodial 21120, Pakistan

**Keywords:** SARS coronavirus, SARS-CoV-2 main protease, structure-based virtual screening, molecular dynamic simulation, hit identification

## Abstract

The unprecedented pandemic of severe acute respiratory syndrome coronavirus 2 (SARS-CoV-2) is threatening global health. SARS-CoV-2 has caused severe disease with significant mortality since December 2019. The enzyme chymotrypsin-like protease (3CLpro) or main protease (M^pro^) of the virus is considered to be a promising drug target due to its crucial role in viral replication and its genomic dissimilarity to human proteases. In this study, we implemented a structure-based virtual screening (VS) protocol in search of compounds that could inhibit the viral M^pro^. A library of >eight hundred compounds was screened by molecular docking into multiple structures of M^pro^, and the result was analyzed by consensus strategy. Those compounds that were ranked mutually in the ‘Top-100’ position in at least 50% of the structures were selected and their analogous binding modes predicted simultaneously in all the structures were considered as bioactive poses. Subsequently, based on the predicted physiological and pharmacokinetic behavior and interaction analysis, eleven compounds were identified as ‘Hits’ against SARS-CoV-2 M^pro^. Those eleven compounds, along with the apo form of M^pro^ and one reference inhibitor (**X77**), were subjected to molecular dynamic simulation to explore the ligand-induced structural and dynamic behavior of M^pro^. The MM-GBSA calculations reflect that eight out of eleven compounds specifically possess high to good binding affinities for M^pro^. This study provides valuable insights to design more potent and selective inhibitors of SARS-CoV-2 M^pro^.

## 1. Introduction

The current global pandemic, so called COVID-19 (COronaVIrus Disease 2019), has spread rapidly since it initially emerged in Wuhan in China, in late 2019 [1,2,3,4]. The virus called ‘severe acute respiratory syndrome coronavirus 2 (SARS-CoV-2)’ is responsible for the outbreak of this pandemic [5]. SARS-CoV-2 belongs to the β coronavirus subgroup of the Coronaviridae family and was found to be related to acute respiratory syndrome coronavirus (SARS-CoV) [6], which previously emerged in China in February 2003 and caused an outbreak in China and spread to several other countries [5,6]. SARS-CoV-2 specifically infects humans by causing an atypical pneumonia, which possesses specific mild to severe symptoms including dry cough, fatigue, fever, shortness of breath, severe progressive pneumonia, multiorgan failure, and eventually death [1]. The World Health Organization (WHO) has declared a state of global health emergency since the outbreak of SARS-CoV-2. According to the World Health Organization (WHO) Coronavirus (COVID-19) dashboard (https://covid19.who.int/, accessed on 30 June 2021), there have been 181,344,224 confirmed cases of COVID-19 globally, including 3,934,252 deaths worldwide, reported to the WHO [7]. Moreover, during the summer of 2020 and spring of 2021, a huge spike was seen in COVID-19 cases [8,9].

SARS-CoV-2 is a positive-sense single-stranded RNA (+ssRNA) virus, with a single linear RNA sequence with ~30,000 nucleotides [10,11,12]. The SARS-CoV-2 virion is 50–200 nanometers in diameter [6], comprising four structural proteins, known as the S (spike), E (envelope), M (membrane), and N (nucleocapsid) proteins, which are encoded by the 3′ end, whereas two viral replicase polyproteins, called pp1a and pp1b, are encoded by the 5′ end of the genome. The N protein holds the RNA genome, while the viral envelope is composed of S, E, and M proteins. S proteins are glycoproteins that are divided into two functional parts (S1 and S2), which are involved in viral attachment and fusion with the membrane of a host cell. pp1a and pp1b proteolytically cleave into 16 non-structural proteins (nsp1 to nsp16) by the main protease and the papain-like protease. nsp5, also called chymotrypsin-like protease (3CLpro) or main protease (M^pro^) located in the pp1a, is essential in the replication and maturation of coronavirus, while the papain-like protease is a deubiquitinase [13,14,15,16,17,18,19,20].

At present, specific antiviral or targeted therapies against SARS-CoV-2 do not exist. However, supportive care, which is augmented by the combination of broad-spectrum antibiotics, antivirals, corticosteroids, and convalescent plasma, is the main treatment approach for COVID-19 [18,19]. The scientific community is involved in extensive research worldwide to formulate suitable therapeutics to control the effects of SARS-CoV-2. Many efforts have been applied to screen existing drugs as potential treatments to eradicate this infection. Since the beginning of the pandemic, several antiviral drugs have been tested in clinical trials against COVID-19, including remdesivir (which was originally designed for the Ebola virus [21]), anti-malarial drugs including chloroquine and hydroxychloroquine [22,23], anti-rheumatoid arthritis drug ‘tocilizumab’ [24,25], and anti-HIV drugs lopinavir/ritonavir [26], among others [27,28]. Nevertheless, the efficacy of some drugs remains controversial. This is the case with a clinical trial involving lopinavir/ritonavir, which reported that no benefits were observed with this treatment compared to standard care [26].

Vaccines against COVID-19 are now available to control the infection [29]; however, there is still an urgent need to discover specific drugs that can target SARS-CoV-2 in patients suffering from COVID-19 due to various emerging variants of the virus. The important targets of SARS-CoV-2 have been identified [30,31] that may be exploited to develop novel therapeutics. Since M^pro^ is one of the key targets of coronavirus, therefore M^pro^ can be targeted to develop antiviral agents. M^pro^ cleaves polyproteins to produce non-structural proteins that are part of the replicase–transcriptase complex. The advantages of targeting M^pro^ are that it specifically exists in the virus and not in humans, it has high sequence identity (i.e., >96%) with SARS-CoV, and it is highly conserved among related viruses [32,33,34,35]. 

**Figure 1:**
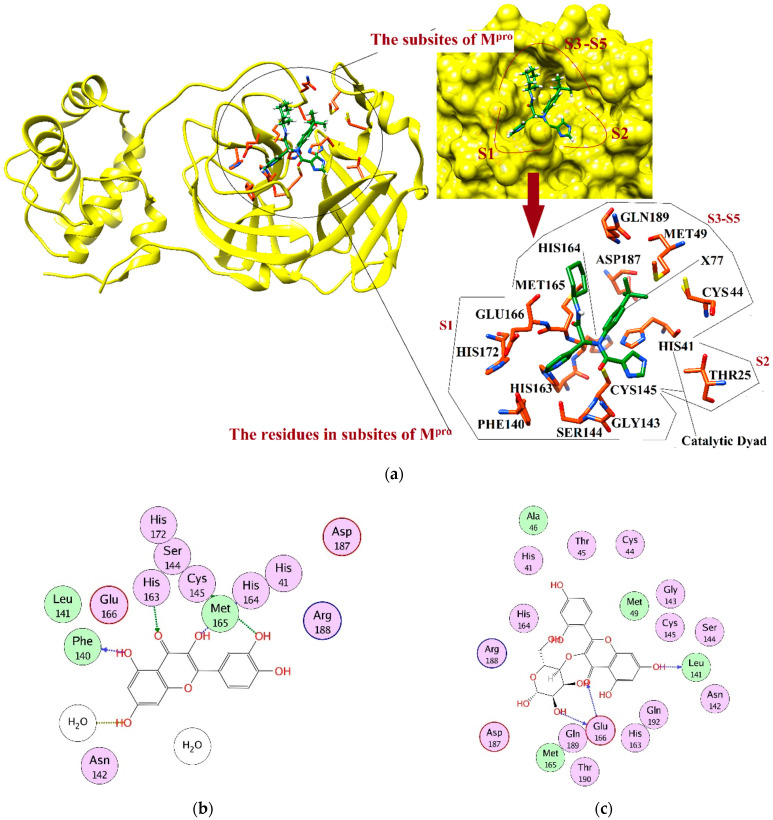

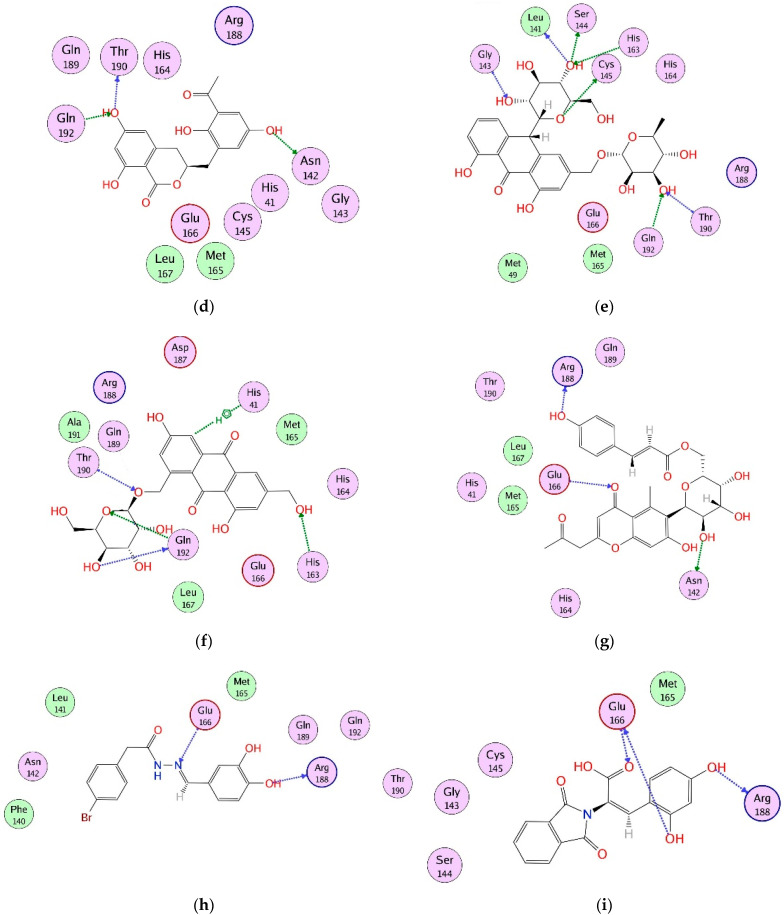

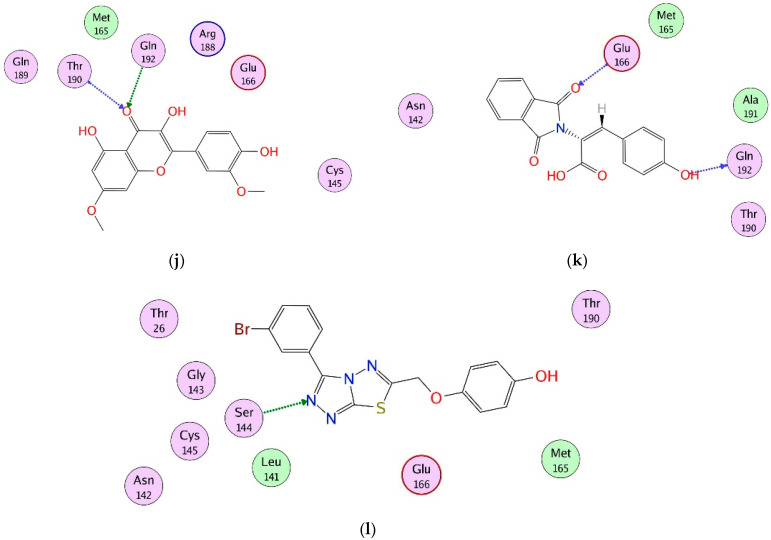
(**a**) The 3D structure of M^pro^ is shown in complex with **X77** (green stick model). The active site residues are displayed in orange stick models. The S1–S5 subsites are highlighted. The protein–ligand binding interactions of compounds **1**, **3**, **6**, **8**, **10**–**13**, **17**, **18**, and **28** are shown in 2D format in (**b**–**l**), respectively. Hydrogen bonds are depicted in dotted arrows. The green and blue colored arrows represent side chain acceptor/donor and backbone acceptor donor atoms, respectively.

M^pro^ is composed of three domains. The domains I and II are composed of 8–101 and 102–184 residues, respectively. These domains acquire a β-barrel shape and resemble chymotrypsin, while domain III (201-306 residues) mainly comprises α-helices. The cleft of domain I and II constitutes the substrate binding region, which consists of the conserved His41 and Cys145 catalytic dyad, where Cys and His act as a nucleophile and a proton acceptor, respectively. Additionally, two deeply buried subsites, called S1 and S2, and three shallow subsites (S3–S5) are also present in the structure. The S1 subsite consists of Phe140, Gly143, Cys145, His163, Glu166, and His172, while S2 consists of Thr25, His41, and Cys145. These residues are mainly involved in hydrophobic and electrostatic interactions. The shallow subsites (S3–S5) are composed of His41, Met49, Met165, Glu166, and Gln189. Despite the high genomic similarity of SARS-CoV-2 with the other members of the coronavirus family, their binding sites have differences in shape and size, which gives us an opportunity to explore more diverse chemical scaffolds by enhanced sampling [32,33,34,35,36]. The three-dimensional (3D-) structure of M^pro^ is depicted in Figure 1a. Herein, we have applied target-specific virtual screening of our in-house compound database with the aim to obtain structurally diverse and potential inhibitors of SARS-CoV-2. Several compounds were identified with high inhibitory potential for M^pro^, and subsequently, could be tested as a treatment against COVID-19.

## 2. Results and Discussion

### 2.1. Validation of Docking Method by Re-Docking and Cross-Docking

Prior to the virtual screening of our in-house database, re-docking and cross-docking of co-crystallized ligands were performed in order to scrutinize the efficiency of the docking method and to select the most appropriate protein file for virtual screening. The re-docking results of twenty protein–ligand complexes showed that 50% of ligands were re-docked with RMSD values of 0.29–1.94 Å, while 30% of ligands were re-docked with RMSD ≤ 3 Å. However, only four ligands showed RMSD in the range of 4 to ≥7 Å. Therefore, 80% of ligands were correctly re-docked in their X-ray-determined conformations. Thus, the docking method was found efficient in predicting the experimentally determined orientations of compounds. RMSD ≤ 3.0 Å is usually considered satisfactory in re-docking experiments; therefore, the results are acceptable. The re-docking results are shown in Table S1.

The cross-docking results (Table S2) showed that 40% of the ligands (ligands in 6Y2F, 6WTK, 6W79, 7BQY, 6ZRT, 7JU7, 6LU7, and 6W63) were correctly ranked between first and third position when docked in their cognate proteins, while two ligands (ligands contained in complexes with PBD codes: 7BRR and 6WNP) were ranked at fifth and seventh position in their X-ray structure. This indicates that MOE accurately ranked 50% of the ligands in good position; therefore, MOE was used in structure-based virtual screening (SB-VS) of our in-house database. The cross-docking results showed that eight proteins (PDB codes: 6Y2F, 6WTK, 6W79, 7BQY, 6ZRT, 7JU7, 6LU7, and 6W63) are appropriate for docking studies; therefore, those proteins were used in the virtual screening experiment. 

#### Analysis of Virtual Screening Accuracy

The predictive accuracy of virtual screening was scrutinized by the ranking or the enrichment of known inhibitors (**KIs**, embedded in the in-house dataset) at the top-ranking position of docked libraries (Table S3). The result was examined by analyzing the percent enrichment factor (%EF) and receiver operating characteristic curves (ROC curves). These matrices are widely used to compare virtual screening results. The results showed that MOE successfully identified **KIs** in 6W79, 7BQY, 6ZRT, 7JU7, and 6W63 with %EF in the range of 33% to 73% at a top-100 position (Table S3), whereas 6W79 showed %EF of 53% at a top-50 position. Moreover, the ROC curve shows an AUC of 0.79–0.84 for 7JU7, 6Y2F, 6WTK, 6LU7, 7BQY, 6W63, and 6ZRT, and 0.90 for 6W79. The %EF and AUC of 6W79 were the highest among all the selected proteins. The ROC curve is displayed in Figure S1.

### 2.2. Selection of Hits after Consensus Approach

The virtual screening of >800 compounds was carried out on multiple structures of M^pro^ (PDB codes: 6Y2F, 6WTK, 6W79, 7BQY, 6ZRT, 7JU7, 6LU7, and 6W63) with the aim of finding out the most potential inhibitors. Later, the consensus approach was used to select the compounds that are ranked among the top 100 positions in all the proteins. We observed that thirteen (**1**–**13**) and eight compounds (**14**–**18**, **28**–**30**) were mutually ranked at a top-100 position in 8/8 and 7/8 proteins, respectively, whereas nine compounds (**19**–**27**) were ranked at a top-100 position in ≥50% of the proteins. Therefore, a total of thirty compounds were selected, and their pharmacokinetic behavior was studied by SwissADME [37] and ADMETsar [38]. The docking results are tabulated in Table S4.

### 2.3. Pharmacokinetic Analysis

The physicochemical properties of the selected compounds showed that the molecular weight of compounds is in the range of 290 to 635 g/mol. A total of 19/30 compounds possess ≤5 rotatable bonds (RBs), while 11/30 compounds possess 6–10 RBs in their structures. The compounds have 3–13 hydrogen bond acceptor atoms (HBA) and 0–9 hydrogen bond donor atoms (HBD). The molar refractivity (MR) and topological polar surface area (TPSA) of these compounds are in the range of 74.33–169.9 and 62.32–226.83 Å^2^, respectively. These results were compared with the physicochemical properties of selected **KIs**. Those **KIs** possess 1–8 HBA atoms, 0–7 HBD atoms, and 1-22 RBs, while 4/15 **KIs** possess a molecular weight in the range of 549 to >681. Similarly, the TPSA of **KIs** was found to be in the range of 20 to >197 Å^2^. The **KIs** including remdesivir, lopinavir and ritonavir also have molecular weight > 600, RB = 15–23 and TPSA in the range of 120 to 203 Å^2^. According to Veber’s rule of drug-likeness [39], TPSA and the number of RBs discriminate between orally active compounds and those that are not orally active for a large dataset of compounds in rats [40]. Therefore, compounds with ≤10 RBs and TPSA ≤ 140 Å^2^ are predicted to have good oral bioavailability [40], while the Ghose filter further improves the predictions of drug-likeness by the following rules: the partition coefficient (LogP) of the compound should be in the range of −0.4 to +5.6, MR = 40 to 130, molecular weight = 180 to 480, and number of atoms from 20 to 70 (including HBDs and HBAs). The predicted partition coefficient (LogP octanol/water) of the selected (30) hits was in the range of +0.25 to 4.74, suggesting their solubility in a hydrophobic medium. The LogP_o/w_ of selected **KIs** is in the range of 0.7 to >5. Similarly, most of the hits demonstrated good to moderate solubility in a water medium. 

The predicted pharmacokinetic properties of the selected hits further helped us to choose more appropriate compounds. The admetSAR showed that all the hits passed human intestinal absorption, while SwissADME showed that 17/30 compounds (**1**, **6**, **12**–**14**, **16**–**19**, **22**–**28**, **30**) had high gastrointestinal absorption (GIA) ability. Similarly, all the compounds (except **16** and **27**) exhibited no blood–brain barrier penetration. Additionally, all compounds (except **15**, **22**–**27,** and **30**) did not have substrate-like properties for P-glycoprotein (P-gp), while compounds **11**, **15**, **19**–**27**, and **30** displayed inhibitory potential against P-gp. Furthermore, most of the compounds were found to be non-inhibitors of cytochrome p450 enzymes (CYP1A2, CYP2C19, CYP2C9, CYP2D6, and CYP3A4). The skin permeation (LogKp) of ligands was found in the range of −5.30 to −10.61 cm/s, showing that these compounds are not permeable through the skin. 

The drug-likeness properties of selected hits were calculated based on the Lipinski rule of five [41] and Ghose [42], Veber’s [39], Egan’s [43], and Muegge’s rules [44]. The compounds **1**, **6**, **12**–**14**, **16**–**18**, **26**, **28** and **30** followed all the drug-likeness criteria given by Lipinski, while compounds **1**, **4**–**6**, **9**, **12**–**14**, **16**–**18**, and **28** fulfilled the Ghose rules of drug-likeness. Similarly, compounds **1**, **6**, **12**–**14**, **16**–**18**, **26**–**28**, and **30** followed Veber’s, Egan’s, and Muegge’s rules of drug-likeness. Comparing these results with the drug-likeness of **KIs** shows that 4/15 **KIs** (including O6K, telaprevir, boceprevir and N3) violate two rules of Lipinski’s drug-likeness criteria, while the known drug, remdesivir, also showed two violations of Lipinski’s rule (MW > 500, HBA > 10), three violations of Ghose rules (MW > 480, MR > 130, number of atoms > 70) and Muegge’s rules (MW > 600, TPSA > 150, HBA > 10), two violations of Veber’s rules (Rotors > 10, TPSA > 140), and one violation of Egan’s rules (TPSA > 131.6). This shows that the selected hits possess comparable drug-likeness with remdesivir. Usually, substrates of biological transporters or natural products do not follow the above-mentioned rules of drug-likeness [45]. Moreover, recently several molecules were approved by the FDA in 2020. Among those approved drugs, several compounds fail on one or the other drug-likeness pharmacokinetic principle, and do not obey Lipinski, Ghose, Veber, Egan, and Muegge’s filters, although this does not question the approval of these molecules. Therefore, it is critical to first look for a potent molecule, and once potency is validated, then to look for improved kinetics [46]. 

The bioavailability score of the compounds was in the range of 0.17–0.56, indicating moderate bioavailability. Among all the selected hits, only a few compounds (**1**, **2**, **4**, **7**, **9**, **10**, **12**, **14**) showed few PAINS alerts, whereas the rest of the compounds did not show any PAINS alerts. Moreover, compounds **1**, **6**, **12**–**14**, **16**–**18** passed the lead-likeness criteria, while compounds **2**, **3**–**5**, **7**–**11**, **15**, and **19**–**30** displayed few violations (i.e., MW > 300, rotors > 7, XlogP 3 > 3.5). The calculated synthetic accessibility of the compounds was in the range of 2.70 to 6.30, which reflects that these compounds are synthesizable. The bioavailability score, lead-likeness, and synthetic accessibility of compounds were compared with remdesivir, which showed that the compounds possess comparable scores. The bioavailability score of remdesivir is also 0.17 and synthetic accessibility = 6.33, and remdesivir depicted two violations in lead-likeness (i.e., MW > 350, Rotors > 7). The predicted physiological properties, pharmacokinetic profiles, drug-likeness, and medicinal properties of the selected compounds are tabulated in Tables S5–S9.

### 2.4. Interaction Analysis

After sequence and structural alignment of eight M^pro^ structures (used in VS), the main pharmacophoric features required for optimal binding were deduced. We observed that His41, Phe140, Gly143, Cys145, His163, His164, Glu166, Gln189, and Thr190 play important roles in the stabilization of protein–ligand binding by providing hydrogen bonds or hydrophobic interactions. Thus, the interactions of the selected ligands with those important residues were scrutinized. The docked view of compound **1** (2-(3,4-dihydroxyphenyl)-3,5,7-trihydroxy-4*H*-chromen-4-one) showed that the compound binds at S1, S2 and S3 subsites, and its hydroxyl groups and the carbonyl moiety formed H-bonds with multiple important residues including Phe140 of S1, Cys145 of S1 and S2, His163 of S1, and His164 of S3. Similarly, the substituted -OH moieties of compound **2** (1R,2R,3*S*,4*S*,6*S*)-6-((E)-3-(3,4-dihydroxyphenyl) acryloyloxy)-2,3,4-trihydroxycyclohexyl 3,4,5-trihydroxybenzoate) formed multiple H-bonds with the side chains of Cys145 (S1 and S2 subsites) and Met165 of S3, and with the main chain carbonyl oxygen of Glu166. Moreover, Glu166 formed bidentate interactions with the -OH group and dihydropyranone oxygen of compound **3** (2-(2,4-dihydroxyphenyl)-5,7-dihydroxy-3-((2R,3*S*,4*S*,5R,6R)-4,5,6-trihydroxy-2-(hydroxy methyl)-tetrahydro-2*H*-pyran-3-yloxy)-4*H*-chromen-4-one), whereas one of the -OH groups of compound **3** mediated H-bonds with Leu141, while the substituted pyran -OH groups of compound **4** (1,3,8-trihydroxy-6-(((2R,3R,4R,5R,6R)-3,4,5-trihydroxy-6-methyl-tetrahydro-2H-pyran-2-yloxy) methyl) anthracene-9,10-dione) interacted with Glu166 and Gln192. Similarly, the -OH and the carbonyl oxygen of compound **5** (2-(2,4-dihydroxyphenyl)-5,7-dihydroxy-3-methyl-4*H*-chromen-4-one) mediated H-bonding with Leu141 and Cys145, respectively. However, the substituted -OH groups of compound **6** ((*S*)-3-(3-acetyl-2,5-dihydroxybenzyl)-6,8-dihydroxy-3,4-dihydroisochromen-1-one) formed H-bonds with Asn142, Thr190, and Gln192. Similarly, Thr190 and Glu166 mediated H-bonds with the -OH groups of compound **7** (1,6-dihydroxy-3-methyl-8-((2R,3R,4R,5R,6R)-3,4,5-trihydroxy-6-(hydroxymethyl)-tetrahydro-2*H*-pyran-2-yloxy) anthracene-9,10-dione). Interestingly, compound **8** ((*S*)-4,5-dihydroxy-9-((2R,3R,4R,5*S*,6R)-3,4,5-trihydroxy-6-(hydroxymethyl)-tetrahydro-2*H*-pyran-2-yl)-2-(((2R,3R,4R,5R,6*S*)-3,4,5-trihydroxy-6-methyl-tetrahydro-2*H*-pyran-2-yloxy) methyl) anthracen-10(9*H*)-one) mediated the highest number of H-bonds with the side chains and backbone atoms of Gly143, Leu141, Ser144, His163, Cys145, Thr190, and Gln192. Therefore, this molecule was considered to be the most promising inhibitor. Moreover, compound **9** (1,3,8-trihydroxy-6-(((1R,2R,3R,4S,5*S*)-2,3,4-trihydroxy-5-methylcyclohexyloxy) methyl) anthracene-9,10-dione) formed multiple interactions with Ser144, Thr190, and Gln192. Like compound **8**, compound **10** (1,6-dihydroxy-3-(hydroxymethyl)-8-(2R,3R,4R,5R,6R)-3,4,5-trihydroxy-6-(hydroxymethyl)-tetrahydro-2*H*-pyran-2-yloxy) methyl) anthracene-9,10-dione) also mediated several interactions with key residues including His163, Thr190, and Gln192, whereas His41 provided H–π interaction to the compound. Similarly, compound **11** ((E)-((2R,3R,4R,5R,6R)-3,4,5-trihydroxy-6-(7-hydroxy-5-methyl-4-oxo-2-(2-oxopropyl)-4*H*-chromen-6-yl)-tetrahydro-2*H*-pyran-2-yl) methyl 3-(4-hydroxyphenyl) acrylate) displayed H-bonding with Asn142, Glu166, and Arg188. The compounds **12** ((E)-*N*′-(3,4-dihydroxybenzylidene)-2-phenylacetohydrazide) and **13** ((E)-3-(2,4-dihydroxyphenyl)-2-(1,3-dioxoisoindolin-2-yl) acrylic acid) formed H-bonds with Glu166 and Arg188, while compounds **14** ((2R,3*S*)-2-(3,4-dihydroxyphenyl)-3,4-dihydro-2*H*-chromene-3,5,7-triol) and **16** (4,11-dibutyl 5,10-bis(2-hydroxyphenyl)-3,12-dithiatricyclo[7 .3.0.02,6]dodeca-1,4,6,8,10-pentaene-4,11-dicarboxylate) interacted with Glu166 and Ser144, and Gly143 and Ser144, respectively. The binding mode of compound **15** ((E)-((2R,3R,5R,6R)-3,4,5-trihydroxy-6-(7-methoxy-5-methyl-4-oxo-2-(2-oxopropyl)-4*H*-chromen-6-yl)-tetrahydro-2*H*-pyran-2-yl) methyl 3-(4-hydroxyphenyl) acrylate) demonstrated that Gln192, Thr190, and His163 stabilized the compound in the active site of M^pro^ through multiple H-bonds, while Thr190 and Gln192, and residues Glu166 and Gln192, provided H-bonds to the compounds **17** (3,5-dihydroxy-2-(4-hydroxy-3-methoxyphenyl)-7-methoxy-4*H*-chromen-4-one) and **18** ((E)-2-(1,3-dioxoisoindolin-2-yl)-3-(4-hydroxyphenyl) acrylic acid), respectively. Compound **19** ((R)-3-((R)-6-(2,2-bis(4-fluorophenyl)ethyl)-4-methoxy-5,6,7,8-tetrahydro-[1,3]dioxolo[4,5-g]isoquinolin-5-yl)-6,7-dimethoxyisobenzofuran-1(3*H*)-one) formed a H-bond with the main chain nitrogen of Glu166, while the amino nitrogen and -OH group of Ser144 and main chain nitrogen of Cys145 stabilized compound **20** (3-{4-[2-carboxy-2-(1,3-dioxo-2,3-dihydro-1*H*-isoindol-2-yl)eth-1-en-1-yl]phenyl}-2-(1,3-dioxo-2,3-dihydro-1*H*-isoindol-2-yl)prop-2-enoic acid) through H-bonding, whereas compounds **21** ((E)-((2R,3R,5R,6R)-3,4,5-trihydroxy-6-(7-hydroxy-5-methyl-4-oxo-2-(2-oxopropyl)-4*H*-chromen-8-yl)-tetrahydro-2*H*-pyran-2-yl)methyl 3-(4-hydroxyphenyl)acrylate)**, 22** ((R)-2-((R)-5-((R)-4,5-dimethoxy-1,3-dihydroisobenzofuran-1-yl)-4-methoxy-7,8-dihydro-[1,3]dioxolo[4,5-g]isoquinolin-6(5*H*)-yl)-2-(9*H*-fluoren-3-yl)ethanamine), **23** ((R)-{6-[2,2-bis(4-fluorophenyl)ethyl]-4-methoxy-2*H*,5*H*,6*H*,7*H*,8*H*-[1,3]dioxolo[4,5-g]isoquinolin-5-yl}[2-(hydroxymethyl)-3,4-dimethoxyphenyl]methanol) and **24** ((*S*)-6-((1-(4-bromobenzyl)-1*H*-1,2,3-triazol-4-yl)methyl)-4-methoxy-5-((R)-5-methoxy-4-methyl-1,3-dihydroisobenzofuran-1-yl)-5,6,7,8-tetrahydro-[1,3]dioxolo[4,5-g]isoquinoline) formed a single H-bond with Cys145, His164, and Glu166, respectively. Moreover, compounds **25** (8-benzyl-*N*-cyclohexyl-14-methyl-7-oxo-5-phenyl-2,3,4,8,18-pentaazatetracyclo [8.8.0.02,6.012,17] octadeca-1(10),3,5,11,13,15,17-heptaene-9-carboxamide) and **26** ((9R,13R)-4-bromo-*N*9-{[(2R)-oxolan-2-yl]methyl}-14-phenyl-8-oxa-14,15,17-triazatetracyclo [8.7.0.02,7.012,16] heptadeca-1(17),2,4,6,10,15-hexaene-9,13-diamine) were stabilized by Glu166 and His163, and Gly143, respectively, while compounds **27** ((9R,13R)-*N*9-benzyl-4-fluoro-14-phenyl-8-oxa-14,15,17-triazatetracyclo[8.7.0.02,7.012,16]heptadeca-1(17),2(7),3,5,10,15-hexaene-9,13-diamine), **28** (4-((3-(3-bromophenyl)-[1,2,4]triazolo[3,4-b][1,3,4]thiadiazol-6-yl)methoxy)phenol) and **30** (4-[(9R,13R)-13-amino-9-(benzylamino)-8-oxa-14,15,17-triazatetracyclo [8.7.0.02,7.012,16] heptadeca-1(17),2(7),3,5,10,15-hexaen-14-yl]benzoic acid) mediated H-bonds with Glu166, Ser144, and Gly143, respectively. The docked view of compound **29** ((*S*)-4,5,9-trihydroxy-2-(hydroxymethyl)-9-((2R,3*S*,4R,5R,6*S*)-4,5,6-trihydroxy-2-(hydroxymethyl)-tetrahydro-2*H*-pyran-3-yloxy) anthracen-10(9*H*)-one) depicts that Glu166 and Phe140 mediated multiple H-bonds with the -OH moieties of **29**, and His41 mediated π–π interaction with this compound. The protein–ligand interactions of **1**–**30** hits are tabulated in Table S10. The binding modes of compounds depict that these compounds mainly bind at S1 and S2 subsites of the active site of M^pro^; however, His164 and Met165 of the S3 subsite, a few residues at the entrance of the active site loop (near S1 subsite including Leu141 and Asn142), and some residues of domain 3 (including Arg188, Thr190, and Gln192) also play important roles in the binding of compounds. Based on the pharmacokinetic profile, drug-likeness, and interaction analysis, eleven compounds (**1**, **3**, **6**, **8**, **10**, **11**, **12**, **13**, **17**, **18**, and **28**) were considered to be good inhibitors; thus, their dynamic behavior was studied by molecular dynamic simulation. The docked orientations and 2D-structures of 11 hits are shown in Figure 1b–l. The chemical structures of compounds **1**–**30** are shown in Figure S2.

### 2.5. Molecular Dynamic Simulation

#### 2.5.1. Convergence of M^pro^ Free and Inhibited States

**Figure 2:**
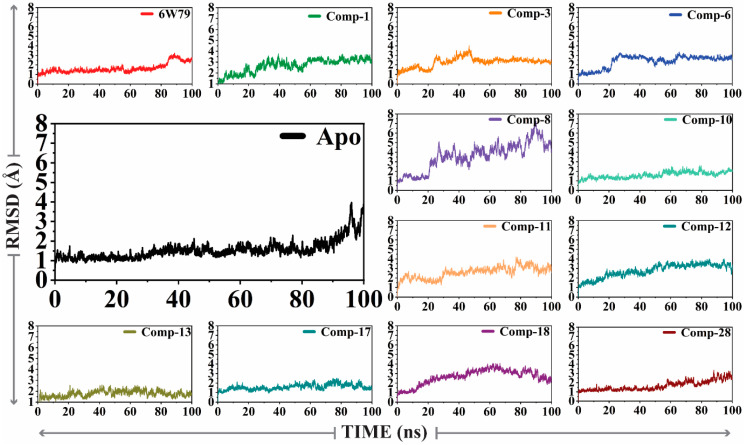
The RMSD plots of apo–M^pro^ and ligand-bound form of M^pro^. The average RMSD of apo–M^pro^ and inhibited M^pro^ was 2.8 Å and <3.9 Å, respectively.

The X-ray structure of M^pro^ (PDB code: 6W79, reported by Mesecar et al. [47]) in complex with the broad-spectrum non-covalent inhibitor (**X77**) was selected for MD simulation as a positive control. The dynamic behavior and structural stability of M^pro^ in the apo form and inhibited states were analyzed through molecular dynamic simulation. The stability of all the complexes was analyzed by calculating the RMSD (alpha carbon, Cα) of all the complexes from the output generated trajectories after 100 ns. The apo–M^pro^ (Figure 2) was stable during the simulation except for the fraction between 96 and 100 ns. The apo–M^pro^ showed an acceptable range of fluctuation and gained stability till 100 ns (showed a smooth and straight graph). This behavior indicates that the free state of the protein was stable. However, the reference complex, M^pro^–**X77**, showed that the RMSD gains equilibrium after 80 ns and is increased up to 100 ns. On the other hand, compound **1** formed several key interactions with the protein, and therefore showed a considerable increase in the RMSD after 25 ns, which affected the overall stability of the complex. A drastic deviation in the RMSD was observed from 20–80 ns during the simulation, whereas the RMSD for the M^pro^–compound **3** complex was found stable till 20 ns, excluding the substantial convergence at the 25–45 ns period where the RMSD of the complex increased significantly. Shortly after the increase in the RMSD, no convergence was seen. Similarly, the M^pro^–compound **6** complex depicted a major stability drift between 20 and 60 ns, while the RMSD remained increased till 100ns. Interestingly, the M^pro^–**8** complex revealed a substantial convergence in the stability until the simulation time. At different levels, significant convergence was observed in the RMSD of the M^pro^–**8** complex. The M^pro^–**10** complex showed a small drift in the convergence at 80 ns; however, the system remained stable. The M^pro^–**11** complex showed drastic shifts in the stability at several intervals of 15–25, 30–60, and 65–85 ns, which significantly affected the stability of the complex. Moreover, the stability of the M^pro^–**12** complex was also affected due to a continuous increase in the RMSD after 40 ns. In contrast, the M^pro^–**13** complex mediated friction between 30 and 40 ns; however, it showed minimal effect on the system’s stability. Similarly, the RMSD of the M^pro^–**17** complex was stable up to 50 ns; however, it increased at 50–60 and 75–90 ns. We observed that the M^pro^–**8** complex depicted a rapid increase in the RMSD between 20 and 90 ns (and therefore destabilized the system); however, after a drastic increase, the RMSD was stabilized after 90 ns. The M^pro^–**28** complex remained relaxed until 55 ns; however, the RMSD was increased after 55 ns and remained elevated throughout the simulation. The sudden increase indicates the fluctuation in the stability of the complex. Altogether, the results indicate that M^pro^–**3,** M^pro^–**8,** M^pro^–**11,** M^pro^–**18,** and M^pro^–**28** complexes attained more variation as compared to the apo–M^pro^, while M^pro^–**8,** M^pro^–**11,** and M^pro^–**28** complexes were found unstable till the end of simulation and reached a maximum RMSD of 7 Å, 4 Å, and 3.9 Å, respectively, as compared to apo–M^pro^ and M^pro^–**X77** complex. During the simulation, no destruction in the simulated complexes (both apo and ligand-bound forms) was observed, which confirms the significance of the simulation. The RMSD graphs of all the complexes are shown in Figure 2.

#### 2.5.2. Root Mean Square Fluctuation (RMSF)

**Figure 3:**
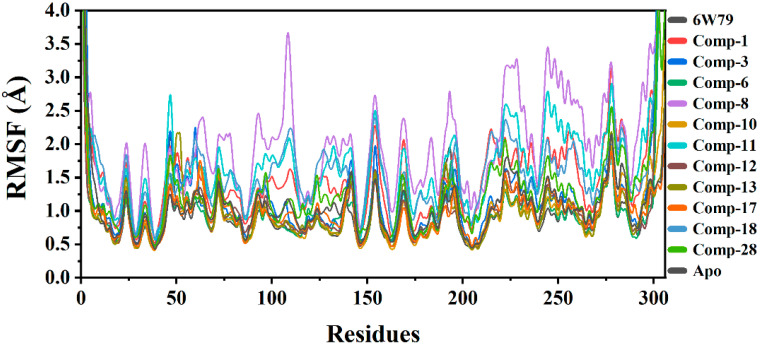
RMSF graphs of apo–M^pro^, M^pro^–**X77**, and M^pro^ in complex with selected hits. The RMSF of free state was in range of 0.5 Å to 1.3 Å. Compound **8** attained highest RMSF (between 1.5 Å and 3.5 Å), while the complex with **X77** showed RMSF between 1.0 Å and 1.8 Å.

RMSF was calculated to observe the fluctuation in different regions of M^pro^ upon ligand binding during the simulation. The main purpose was to see the effects of ligand binding on the flexibility of each residue of protein. The RMSF graphs of all complexes and apo–M^pro^ are shown in Figure 3. The results show that compound **8** increased the flexibility of all regions of the M^pro^–**8** complex as compared to the apo–M^pro^, M^pro^–**X77** complex, and complexes of the rest of the selected hits from the in-house database. The apo–M^pro^ showed the lowest RMSF as compared to the ligand-bound states, reflecting that the protein is not very flexible in the un-ligated state. The average RMSF of all the systems was found in the range of 1.5 Å. The M^pro^–**3,** M^pro^–**8,** M^pro^–**11,** and M^pro^–**28** complexes exhibited high flexibility, while the flexibility of M^pro^–**10** and M^pro^–**13** complexes was low. The loops in the protein structure fluctuated the RMSF at different regions. The M^pro^–**1,** M^pro^–**6,** M^pro^–**12,** M^pro^–**17,** and M^pro^–**18** complexes depicted lower flexibility due to the differential dynamics upon ligand binding. The secondary structures with loops are responsible for the fluctuation in the RMSF at different levels, justifying the residual flexibility. The flexibility of complexes with the selected eleven hits varies as compared to the apo–M^pro^ and **X77**-inhibited states (Figure 3).

**Figure 4:**
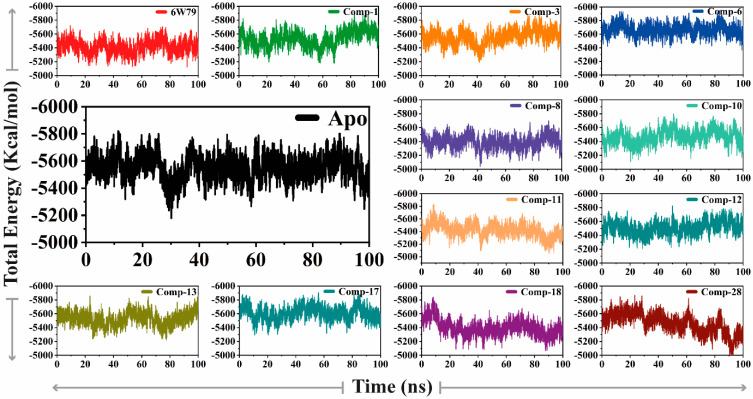
The difference of total energy of M^pro^ is shown in the apo–M^pro^ and inhibited states. The *x*-axis and *y*-axis depict time (nanoseconds) and the total energy of the protein during the simulation (Kcal/mol), respectively.

The total energy of the apo–M^pro^ was stable (energy = −5600 kcal/mol), while the M^pro^–**X77** exhibited slightly lower energy (−5400 kcal/mol) as compared to apo–M^pro^. The M^pro^–**8,** M^pro^–**11,** M^pro^–**18,** and M^pro^–**28** complexes revealed a similar energy pattern (in the range of −5400 kcal/mol to −5200 Kcal/mol), whereas the M^pro^–**10** and M^pro^–**13** complexes possess slightly higher total energy (between −5500 kcal/mol and −5700 kcal/mol) than the apo–M^pro^. The M^pro^–**1,** M^pro^–**3,** M^pro^–**6,** M^pro^–**12,** and M^pro^–**17** complexes showed increased total energy ranging from −5600 kcal/mol to −5800 kcal/mol (Figure 4). The inhibited states of M^pro^ shared similar patterns and variations as compared to the apo form, therefore showing the effects of deviation on the structure of the protein created by each inhibitor.

The total energy of the apo–M^pro^ was stable (energy = −5600 kcal/mol), while the M^pro^–**X77** exhibited slightly lower energy (−5400 kcal/mol) as compared to apo–M^pro^. The M^pro^–**8,** M^pro^–**11,** M^pro^–**18,** and M^pro^–**28** complexes revealed a similar energy pattern (in the range of −5400 kcal/mol to −5200 Kcal/mol), whereas the M^pro^–**10** and M^pro^–**13** complexes possess slightly higher total energy (between −5500 kcal/mol and −5700 kcal/mol) than the apo–M^pro^. The M^pro^–**1,** M^pro^–**3,** M^pro^–**6,** M^pro^–**12,** and M^pro^–**17** complexes showed increased total energy ranging from −5600 kcal/mol to −5800 kcal/mol (Figure 4). The inhibited states of M^pro^ shared similar patterns and variations as compared to the apo form, therefore showing the effects of deviation on the structure of the protein created by each inhibitor.

#### 2.5.3. Protein Motions and Trajectories Clustering

**Figure 5:**
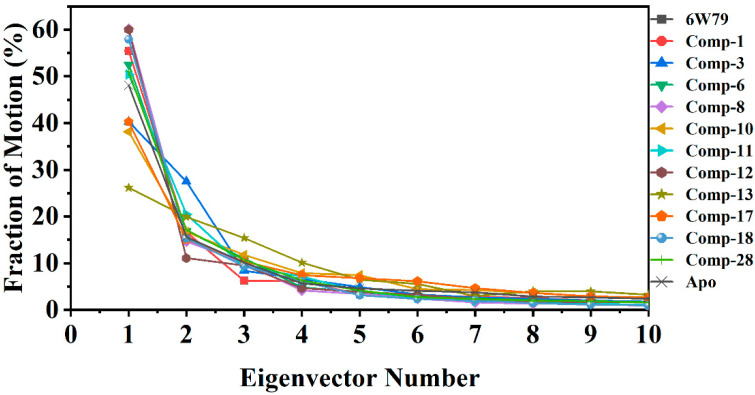
First 10 eigenvector fractions are shown. Each eigenvector contribution (in percentage, %) is acquired from covariance matrix plotted against the corresponding eigenvector from MD trajectory of each complex.

The dynamic impact of eleven hits on the structure of M^pro^ is shown in Figure 5. The structural changes in each complex due to the protein–ligand binding was observed through principal component analysis (PCA). The significant dominant motions (Figure 5) are shown in the first three eigenvectors, while the others indicated localized fluctuation. In the apo–M^pro^, a total of 48% of variances were contributed by the first three eigenvectors to the total observed motion. Unlike the apo–M^pro^, the inhibited states showed different behavior of motion. In inhibited states, compounds **8** and **12** showed 60%, compounds **18, 1** and **X77** reflected 57% and 55%, respectively, whereas compounds **6, 28,** and **11** showed 52–50% of total motion. The total motion of compounds **3** and **17** was 40%, while compounds **10** and **13** demonstrated least motion of 38% and 26%, respectively. These structural behavior clearly demonstrated the structural rearrangement of the protein upon ligand binding.

**Figure 6:**
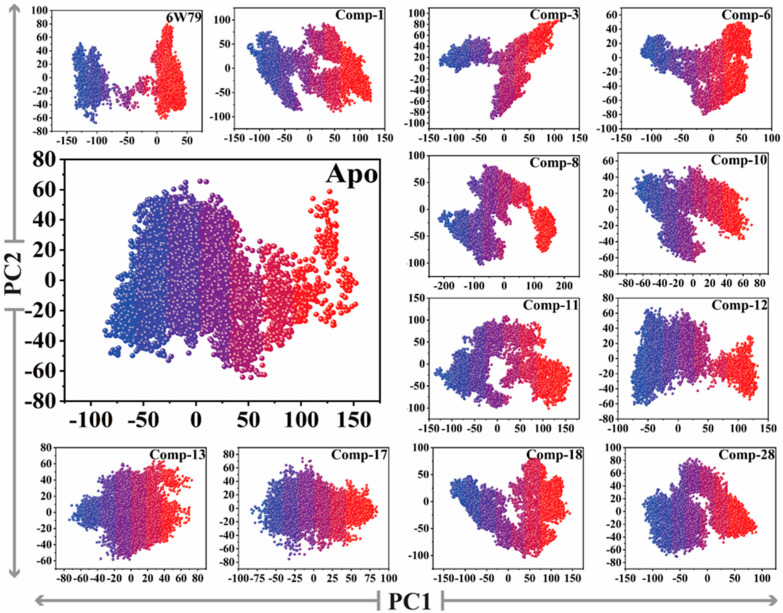
The PCA analysis of apo–M^pro^ and inhibited states of M^pro^. Principal component 1 (PC1) and principal component 2 (PC2) were plotted against each other using the backbone carbon atoms.

The reliability of attributed motions was achieved by plotting the two initial eigenvectors of each trajectory against each other. During the simulation production run, the flipping over conformation was shown by color blue to red. The dots represented each frame from blue to red. To understand the conformational transformation of the complexes, a 2D subspace was mapped from the trajectories using PC1 and PC2. Figure 6 clearly shows that each complex acquired two conformational states on the subspace differentiated by the colors (blue and red). The unstable conformational state (shown in blue) can be easily separated in neared convergence to obtain a stable conformational state (shown in red). Subsequently, the apo–M^pro^ showed more energetic conformation, while the inhibited states showed stable energy conformation with different periodic jumps. The M^pro^–**X77** complex reflected very stable lower energy conformation as compared to apo–M^pro^, while the rest of the inhibitors followed the same pattern and acquired stability with lower energy states.

#### 2.5.4. Metastable to Native State Transition Pathway

**Figure 7:**
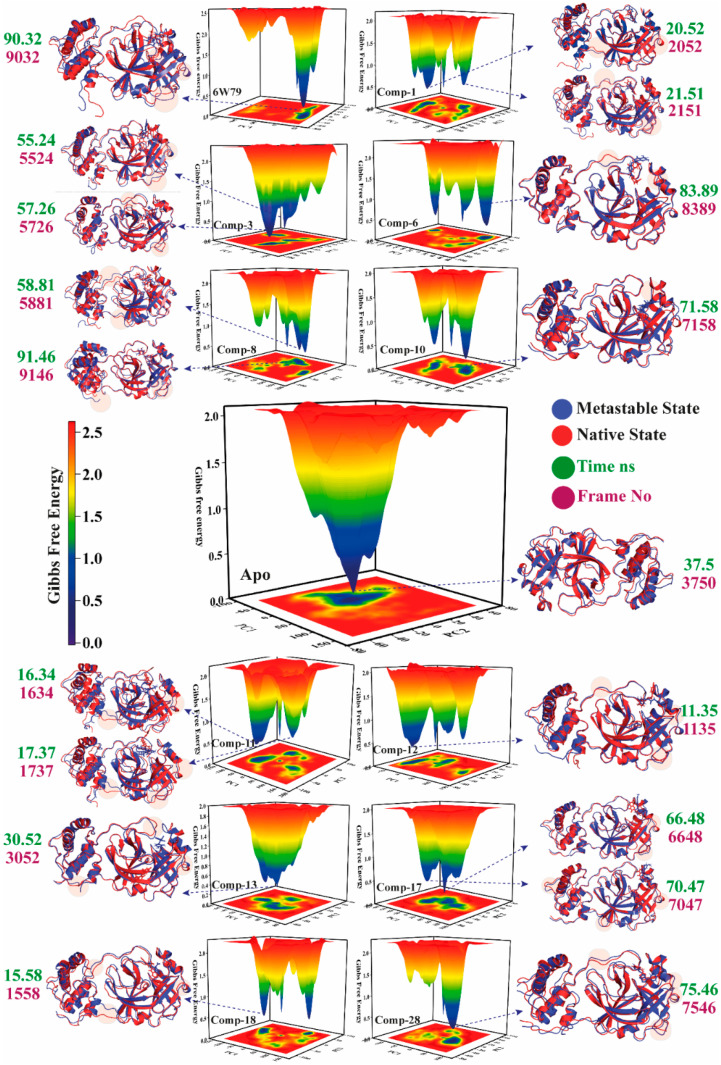
The free energy landscape (FEL) of free state and inhibited states is shown. High and low energy states are shown in distinct colors in the plot. The minimal, intermediate, and the high energy states are presented in dark blue, yellow, and red, respectively. The time of the metastable states (ns) and frame number is presented in green and purple, respectively. The metastable states and the native structures of M^pro^ are depicted in cartoon model in blue and red, respectively.

The transition states of the apo–M^pro^ and inhibited M^pro^ complexes were studied using the free energy landscape (FEL). The FEL plot was constructed from the first two eigenvectors of the trajectory time to explore the transition mechanism from the metastable state to the native state. The lowest energy states of each complex were examined to investigate the structural changes. The apo–M^pro^ showed a significant change in the energy states as compared to the inhibited states (represented by red, yellow, green, and blue in Figure 7). The highest energy levels and the metastable stage in the plots are shown by red and blue colors, respectively. The apo–M^pro^ was stable as compared to the inhibited states because the red color (high energy state) is more prominent in the inhibited states (**X77**, **1**, **3**, **6**, **8**, **10**–**13**, **17**–**18**, and **28**). The compounds **3**, **8**, **6**, **11**, **18**, and **28** showed the highest transition states due to the interaction with the active site domain of M^pro^. The apo–M^pro^ acquired only one state with no energy barriers, and similarly, compound **13** showed a pattern like apo–M^pro^ due to the sliding of compound **13** from the active pocket because of weak interactions. Moreover, compound **10** acquired two states with a stable energy level for the maximum time (shown in yellow). The reference ligand, **X77**, remained mostly in the high energy state, which confirmed the effect on the stability of the protein structure due to the rearrangement of the bonds upon binding with **X77**. Figure 7 depicts that the apo–M^pro^ remains in the green and yellow energy states, while ligand-inhibited complexes are found in the high energy state (red) for most of the simulation time. The inhibition of M^pro^ by the selected hits is evident by FEL, which clearly shows the structural rearrangement of the protein upon binding with small drug-like molecules. The ligand-bound complexes (inhibited states) displayed more conformational transitions as compared to the free state. Various metastable states showed conformational changes in the M^pro^ structure in the ligand-inhibited complexes. The protein structure was ensembled at a distinct nanosecond time scale. In Figure 7, the crucial areas in the structures are shown in shaded form. The X and Y coordinates were obtained from the metastable states from all the trajectories with their respective frame number and time (ns), which are tabulated in Table 1.

#### 2.5.5. Dynamic Cross-Correlated Map Analysis

**Figure 8:**
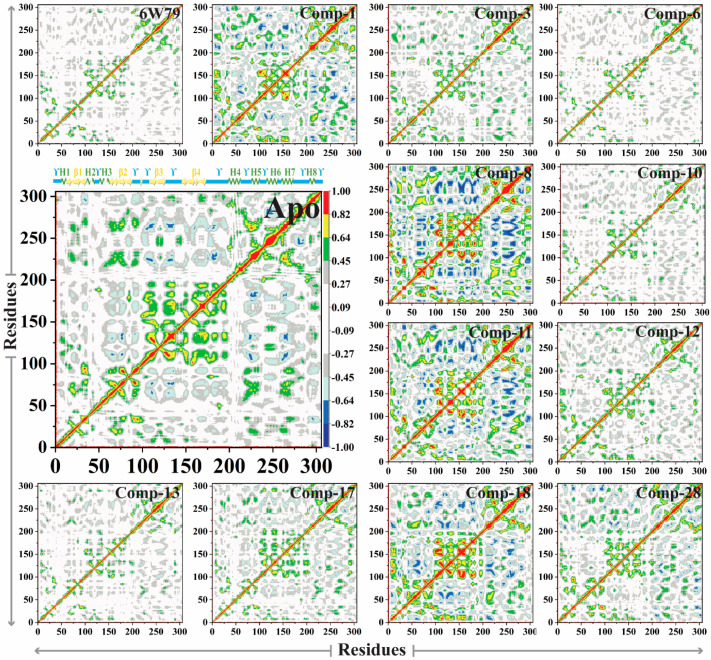
The DCCM plot of apo– and inhibited states of M^pro^ is shown. The positive (green, yellow, and red) and the negative correlation motion (dark blue, light blue, and cyan) are shown in different colors. The incline in color (from one to another) indicates a slow decline in the correlation motion.

The dynamic cross-correlation matrix (DCCM) was constructed to elaborate the functional displacements of the protein’s interactive atoms as a function of time. The apo–M^pro^ reflected more positive correlation motion during 100 ns of simulation, while the dominant-negative correlation motion of the loop was observed. The inhibited M^pro^ demonstrated variation in correlated motion, where maximum residues of the inhibited M^pro^ showed positive correlation motion compared to the apo–M^pro^. The correlation motion of all the systems is graphically presented in Figure 8. The overall motions in each system are dominated by the correlated motions. In the **X77**-inhibited M^pro^, the β1 and β2 displayed negative correlation motion and ϒ4 and ϒ5 loops showed positive correlation motion, while in the M^pro^–**1** complex, ϒ2, ϒ3, and ϒ4 reflected negative correlation motion, while β1, β2, and β4 displayed positive correlation motion. On the other hand, compounds **8**, **11**, and **18** showed negative high correlation motion in loops ϒ3, ϒ4, and ϒ5, while the β1, β2, β3, and β4 regions acquired apparent positive correlation motion. The compounds **6**, **10**, and **13** showed similar patterns as compared to the apo–M^pro^, while they displayed insignificant positive correlation motion in ϒ4 and ϒ5 and negative correlation motion at β1 to β2. However, compound **17** did not show significant positive correlation motion in ϒ3, ϒ4, and β4 regions of M^pro^. Furthermore, compound **28** showed a weak negative correlation motion in β1 and ϒ1 regions, and slight positive correlation motion at β4. Hence, the internal dynamics of the protein have substitution effects upon ligand binding with the protein. The results indicate that dynamic variability and conformational changes were caused by small inhibitors, therefore revealing the affinity of ligands toward M^pro^.

#### 2.5.6. Binding Free Energy Calculations

The binding free energy of each ligand was estimated to quantitatively compare the energy differences of the selected hits (from the in-house database) with **X77**. The binding free energy was computed from the last 1000 frames of the 100 ns of MD trajectory. MM-GBSA analysis was performed for each system by calculating each contributing energy, such as van der Waals (∆VDW), total electrostatic energy (∆EET), polar and non-polar contributions (∆EGB), and non-polar solvation energy (SASA) (Table 2). The MM-GBSA results showed variation in energies among **X77** and the eleven molecules. The effect is high in terms of total and electrostatic energies. The reference inhibitor, **X77**, exhibited ∆VDW (−46.7396 Kcal/mol), ∆EEL (−7.2011 Kcal/mol), ∆EGB (22.3537 Kcal/mol), and ∆SASA (−5.6612), with the total energy (∆G_TOTAL_) of −37.2483 Kcal/mol, while compounds **11** and **28** reflected total energies of −33.6485 and −33.6723 Kcal/mol, respectively, which varies slightly from **X77**, with a decrease in the ∆VDW (compound **11** = −39.9829, comp. **28** = −41.1238 Kcal/mol) and an increase in the ∆EEL (compound **11** = −15.1839, compound **28** = −7.7362 Kcal/mol). Both **11** and **28** exhibited the highest binding free energy among the eleven hits. Furthermore, compounds **3**, **8**, and **18** showed ∆G_TOTAL_ of −26.9034 Kcal/mol, −26.5848 and −24.7101 Kcal/mol, respectively, which reflects the good affinity of these compounds for M^pro^. Compounds **1**, **3**, and **17** also showed appropriate binding potential with M^pro^ by making stable complexes with ∆G_TOTAL_ of −22.7848 Kcal/mol, −22.9067 Kcal/mol, and −22.0295 Kcal/mol, respectively. However, compounds **10** and **13** reflected the lowest total binding free energies (compound **10** = −6.4968 Kcal/mol and **13** = −9.4012 Kcal/mol) due to poor binding interactions within the active site of M^pro^.

The ∆SASA energy of compound **8** was significantly higher than the M^pro^–**X77** complex, indicating that **8** has greater impact on the structure of the protein. The total energies of compounds **1**, **3**, **6**, **8**, **11**–**12**, **17**–**18**, and **28** indicate that these compounds exhibit inhibitory potential by specifically binding with the active site of the SARS-CoV-2 M^pro^.

## 3. Materials and Methods

### 3.1. Preparation of Protein’s Structures for Docking

The re-docking and cross-docking experiments were carried out in order to examine the efficiency of the docking method. For re-docking, twenty protein–ligand complexes were taken from Research Collaboratory for Structural Bioinformatics Protein databank (RCSB-PDB). The complexes were chosen based on good resolution (<2.5 Å, Table S11). Only water molecules within the 3 Å of co-crystallized ligand molecule were retained in the protein files, while the rest were removed. Moreover, other heteroatoms (other than ligands) were also deleted from each file. The protein files were imported in MOE interface [48], where proteins were prepared for docking by adding hydrogen atoms and molecular charges using MOE Protonate 3D tool. Each protein was parameterized by MOE Potential setup using Amber12:EHT force field.

#### Preparation of Compound Database for Docking

For re-docking and cross-docking, the ligands were extracted from the selected proteins (Table S11), their atom types were corrected, hydrogen atoms were added and partial charges were applied using MOE Potential setup (Amber12:EHT force field) [48,49]. Subsequently, each ligand was minimized with Amber12:EHT force field (eps = r, and Cutoff (8, 10)) with RMS gradient of 0.1kcal/mol/Å. Each ligand was imported into MOE database for re-docking, cross-docking, and virtual screening experiments. For virtual screening, the chemical entities were collected from our institute (Natural and Medical Sciences Research Center, University of Nizwa, Oman) [50], which has a diverse set of compounds, originating from natural and synthetic sources. Virtual screening was conducted on our in-house molecular database, comprising >800 chemical compounds. The structures of compounds in the library are given in SMILE format in the Supplementary Materials. Moreover, fifteen known inhibitors (**KIs**) were also added in our in-house database as positive controls (Table S11). The 3D-structure of each ligand (in mol2 format) was imported into MOE compound database, where Wash module of MOE was used to add hydrogen atoms and partial charges (based on Amber12:EHT force field) on each structure, and the structures were minimized with the same parameters as discussed above.

### 3.2. Structure-Based Screening by Molecular Docking

After the preparation of protein and ligand files, molecular docking was performed by Triangle Matcher docking algorithm and London dG scoring function [48,51,52]. The active site/ligand binding site was defined on the co-crystallized ligand in each protein. For re-docking, the ligands were extracted from each protein and re-docked in its cognate binding protein (with the above-mentioned settings), and the results were quantified by calculating root mean square deviation (RMSD) between the docked and X-ray conformation of each ligand. Similarly, cross-docking was performed by docking all the twenty (extracted) ligands in each of twenty proteins and results were examined by ranking (at top position) of compounds in their native X-ray crystal structure. By default, thirty docked conformations of each ligand were obtained. The virtual screening of in-house database was performed on those PDB files that displayed good results in cross-docking experiment.

#### Analysis Measures and Conformational Sampling after Virtual Screening

The inhibitor with the most potential against SARS-CoV-2 M^pro^ was chosen after virtual screening by consensus approach. The in-house library was docked in eight protein structures individually. Later, each docked library was sorted based on the docking score, and those compounds that were ranked mutually in ‘Top-100’ position in at least 50% of the structures were declared as potential ‘Hits’. The optimal binding modes of the selected compounds were chosen through conformational sampling. The docked orientation of each compound found analogous in all the proteins was considered as the possible binding mode. The interactions of ligand were visualized by Protein–Ligand Interaction Fingerprints (PLIF) [48] of MOE, which calculates several types of interactions between protein and ligands including H-bonds, water-mediated protein–ligand bridging, ionic interactions, surface contacts, metal ligation, and arene attraction in 2D format.

### 3.3. Prediction of Pharmacokinetic Properties

After virtual screening, the pharmacokinetic (ADMET: absorption, distribution, metabolism, excretion, and toxicity) behavior of the selected compounds was studied through SwissADME [37] and admetSAR [38], which predicts ADMET properties and drug-likeness of small molecules by using physicochemical descriptors.

### 3.4. Molecular Dynamic Simulation

The atomic coordinates of PDB ID: 6W79 [47] were chosen for the molecular dynamic simulation studies. Thirteen systems were generated for MD simulation, including apo form of 6W79 (apo–M^pro^), 6W79 in complex with co-crystallized ligand (M^pro^–**X77**), and 6W79 in complex with docked conformations of eleven hits. The apo–M^pro^ and M^pro^–**X77** complex were used as positive controls. The possible overlaps/clashes in the initial structure were eliminated by minimizing the structure with 10,000 cycles of steepest descent [53] (macromolecule was frozen), followed by 20,000 cycles of conjugate gradient method [54]. LEaP module of AMBER20 [55] was used to add the missing hydrogen atoms. To keep the systems neutral, counter-ions from OPC model [56] were added. A truncated octahedral box of the OPC water model [57] was added to all the systems with a 10 Å buffer (8 Å cut-off was used to compute the pairwise interactions, the van der Waals, and direct Coulomb forces). Long-range electrostatic forces were treated with the particle mesh Ewald (PME) algorithm [58]. The intermolecular interactions were calculated by ff19SB [59]. In preparation runs, Langevin thermostat [60] was used with 1 ps^−1^ friction constant, while Berendsen thermostat [61] was used in the production runs. MD simulation was accelerated using the PMEMD CUDA version in GPU cores. Before running MD production, all the systems were heated for 400 ps, followed by equilibration of up to 2000 ps in the NVT ensemble at 300 K. The conditions applied in the simulation of all systems are given in Table 3.

#### 3.4.1. Post-Dynamic Evaluation

The coordinates of all the simulated systems were extracted from the generated trajectories after every 1 ps and analyzed by PTRAJ [62] module of the AMBER20. The Root Mean Square Deviation (RMSD), Root Mean Square Fluctuation (RMSF), and radius of gyration (Rg) of all the systems were calculated by CPPTRAJ module of AMBER20 on Cα atoms via Equations (1)–(3).

(1) $$\mathrm{RMSD}=\sqrt{\frac{\sum_{i=0}^{N}[m_{i}*\left(X_{i}-Y_{i}{)}^{2}\right]}{M}}\;$$

In RMSD calculation, N = the number of atoms, mi = the mass of atoms, Xi = the target atom i vector coordinate, Yi = the reference atom i vector coordinate, and M = the total mass.

(2) $$\mathrm{RMSF}\left(i\right)=\sqrt{\;\langle {\left(x_{i}-\langle x_{i}\rangle \right)}^{2}\rangle }$$

The RMSF of selected atom i was calculated as: the atomic positions averages over the total input frames (denoted by x).

(3) $$\mathrm{Rg}=\sqrt{\frac{1}{N}\overset{i=0}{\underset{N}{\sum }}{\left(r_{i}-r_{m}\right)}^{2}}$$

The Rg of N number of atoms was calculated: the atomic position was denoted by ri, and the mean position was denoted by r_m_ of all the atoms. The Altona and Sundaralingam method [63] was used to calculate the five-membered ring pucker. Standard deviation and averages were reported in the analysis utilities, with proper cyclic averages being computed for periodic values (torsions). Furthermore, the total energy of all the systems (apo– and inhibited states) was calculated.

#### 3.4.2. MD Trajectories Unsupervised Clustering and Free Energy Landscape

Principal component analysis (PCA, focuses on matrix covariance) was used to demonstrate atom movement and protein loop dynamics. The internal motions of the systems were analyzed by PCA approach of CPPTRAJ. The atomic coordinates of eigenvectors and the positional covariance matrix were calculated. The orthogonal coordinate transformation was used to obtain the eigenvalue diagonal matrix by the diagonalizing of the matrix. The principal components were obtained based on eigenvalues and eigenvectors to emphasize the motion of the atoms in MD simulation trajectories. The isolated first principal components, PC1 and PC2, showing the largest variation in the data, were utilized for the free energy landscape (FEL) using Equation (4) from 100 bins of the data population. The energies were calculated in kcal/mol at 300 °K.

(4) $$G_{i}=-K_{B}T\left(\frac{N_{i}}{N_{\mathrm{Max}}}\right)$$

where K_B_ = Boltzmann’s constant, T = specified temperature, N_i_ = bin_i_ population, and N_Max_ = most populated bins. The artificial barrier population size of 0.5 was applied to the bins with no population.

#### 3.4.3. Dynamic Cross-Correlation Analysis (DCC)

The dynamic cross-correlation map (DCCM) method was used to obtain the Cα atom’s time subordinate movements caused by the attachment of a small molecule (inhibitor) with the protein. The correlation matrix was derived by observing the Cα atoms’ correlated and anti-correlated motions of each system. DCCM was calculated by Equation (5).

(5) $$C_{\mathrm{ij}}=\langle {\Delta r}_{i}\times {\Delta r}_{j}\rangle /{\left(\langle {\Delta r}_{i}^{2}\rangle \langle {\Delta r}_{j}^{2}\rangle \right)}^{2}$$

where C_ij_ = time correlated data between the atoms i and j in a protein. We used 0.002 ns interval to construct the matrix of Cα from the 10,000 snapshots. The positive and negative values indicate the correlated and anti-correlated motion during the MD simulation, respectively, in the matrix plot.

#### 3.4.4. MM/GBSA Free Energy Calculation

In MD simulation, free energy calculations give quantitative production of protein–ligand binding energies. The binding energy (G_bind_) was calculated by Equation (6).

(6) $$G_{\mathrm{bind}}={\;G}_{R+L}-\left(G_{R}+G_{L}\right)$$

where G_R+L_ represents the M^pro^ in complex with inhibitors, while G_R_ and G_L_ represent the apo–M^pro^ and inhibited M^pro^, respectively.

In the generalized born surface area (MM/GBSA) approach, each free energy term in Equation (6) was calculated using Equation (7).

(7) $$G=E_{\mathrm{bond}}+E_{\mathrm{VDW}}+E_{\mathrm{elec}}+G_{\mathrm{GB}}+G_{\mathrm{SA}}-{\mathrm{TS}}_{S}$$

where E_bond_ represents bond angles and dihedral energy, E_vdw_ and E_elec_ indicate the contribution of van der Waals and electrostatic energy, respectively, while the related polar and non-polar contribution of solvation energy are reported as G_GB_ and G_SA_. T and S_s_ show the absolute temperature of the system and the solute entropy, respectively.

The performance of the MMGBSA algorithm is based on the specificity of the forcefield and inhibitor’s partial charges, the specificity of protein–inhibitor complex, MD simulation, inner dielectric constant, and the docking pose number based on top scoring. Here, the binding free energies of each system were calculated by MM/PB(GB)SA model of GBSA. The solvent probe of 2 Å radius was used, and the radii were used to optimize the topology files.

#### 3.4.5. Data Analysis

The results were analyzed by MOE [48], UCSF Chimera [64], and Pymol [65]. The average structures were extracted from structure ensembles of the lowest energy. All the analysis graphs were plotted using Origin pro [66] and GnuPlot [67].

## 4. Conclusions

The main protease or chymotrypsin-like protease of SARS-CoV-2 is considered to be a potential anti-viral drug target. We have employed an efficient structure-based virtual screening protocol to search for novel inhibitors of SARS-CoV-2. The binding potential of several compounds was tested on multiple structures of M^pro^, and consensus strategy was applied to select the most promising binders. Based on the physiological and pharmacokinetic behavior and protein–ligand binding pattern, eleven compounds were identified as good inhibitors of M^pro^. Therefore, the structural and dynamic behavior of M^pro^ upon binding with those eleven compounds was further explored through molecular dynamic simulation. Based on the MM-GBSA calculations, two compounds (**11** and **28**) were retrieved with the highest binding affinities for M^pro^, whereas six compounds (**3**, **8**, **18**, **6**, **1**, and **17**) showed good binding affinities for M^pro^. Based on our in silico findings, we suggest that these compounds can inhibit the replication of SARS-CoV-2 by specifically inhibiting its M^pro^ enzyme. Therefore, these compounds can act as potential anti-viral candidates against SARS-CoV-2. However, further in vitro testing is required to confirm these in silico results.

## Figures and Tables

**Table 1:** The X and Y coordinates of metastable states with frame number and time (ns).

Complex Name	X Coordinates	Y Coordinates	Frame No	Time ns
Apo–M^pro^	1.859	−0.699	3750	37.5
M^pro^–**X77**	29.197	−20.599	9032	90.32
M^pro^–**1**	−73.840	3.321	2052	20.52
	−71.395	1.503	2151	21.51
M^pro^–**3**	1.713	−50.468	5524	55.24
	6.430	−46.903	5726	57.26
M^pro^–**6**	34.503	34.145	8389	83.89
M^pro^–**8**	7.810	45.191	5881	58.81
	136.017	−19.440	9146	91.46
M^pro^–**10**	28.891	3.061	7158	71.58
M^pro^–**11**	−89.371	−31.716	1634	16.34
	−86.385	−25.418	1737	17.37
M^pro^–**12**	−54.007	−18.018	1135	11.35
M^pro^–**13**	−29.833	3.606	3052	30.52
M^pro^–**17**	27.298	−4.897	6648	66.48
	33.972	−6.394	7047	70.47
M^pro^–**18**	−91.981	0.876	1558	15.58
M^pro^–**28**	37.636	−1.614	7546	75.46

**Table 2:** The MMGBSA analysis of **X77** and eleven hits for M^pro^.

Complex Name	Kcal/mol				
	∆_VDW_	∆_EEL_	∆_EGB_	∆_SASA_	∆_G TOTAL_
M^pro^–**X77**	−46.7396	−7.2011	22.3537	−5.6612	−37.2483
M^pro^–**1**	−27.9473	−11.8236	20.6925	−3.7064	−22.7848
M^pro^–**3**	−36.3657	−28.7266	43.2732	−5.0843	−26.9034
M^pro^–**6**	−34.5680	−15.4295	31.3375	−4.2467	−22.9067
M^pro^–**8**	−41.0277	−15.6216	36.141	−6.0766	−26.5848
M^pro^–**10**	−10.8292	−5.7753	11.5464	−1.4387	−6.4968
M^pro^–**11**	−39.9829	−15.1839	25.9731	−4.4549	−33.6485
M^pro^–**12**	−25.5687	−13.1445	22.4730	−3.8878	−20.1279
M^pro^–**13**	−15.3599	−5.7322	13.8263	−2.135	−9.4012
M^pro^–**17**	−25.1406	−18.6810	25.3125	−3.5204	−22.0295
M^pro^–**18**	−33.2958	−9.3268	22.2269	−4.3144	−24.7101
M^pro^–**28**	−41.1238	−7.7362	19.6643	−4.4767	−33.6723

**Table 3:** The conditions used in the molecular dynamics of apo– and inhibited states of M^pro^.

S. No.	System Composition (Complexes)	Temperature (K)	Force Fields	Water Model	Time (ns)
1	Full length apo–M^pro^	300	FF19SB	Octahedral OPC	100
2	M^pro^–**X77** (6W79)	300	FF19SB+Gaff2	Octahedral OPC	100
3	M^pro^–**1**	300	FF19SB+Gaff2	Octahedral OPC	100
4	M^pro^–**3**	300	FF19SB+Gaff2	Octahedral OPC	100
5	M^pro^–**6**	300	FF19SB+Gaff2	Octahedral OPC	100
6	M^pro^–**8**	300	FF19SB+Gaff2	Octahedral OPC	100
7	M^pro^–**10**	300	FF19SB+Gaff2	Octahedral OPC	100
8	M^pro^–**11**	300	FF19SB+Gaff2	Octahedral OPC	100
9	M^pro^–**12**	300	FF19SB+Gaff2	Octahedral OPC	100
10	M^pro^–**13**	300	FF19SB+Gaff2	Octahedral OPC	100
11	M^pro^–**17**	300	FF19SB+Gaff2	Octahedral OPC	100
12	M^pro^–**18**	300	FF19SB+Gaff2	Octahedral OPC	100
13	M^pro^–**28**	300	FF19SB+Gaff2	Octahedral OPC	100

## Data Availability

All the data are included in this paper.

## Supplementary Materials

The following are available online at https://www.mdpi.com/article/10.3390/ph14090896/s1, Table S1: Re-docking results of MOE, Table S2: The cross-docking analysis of MOE, Calculation of Enrichment Factor (EF) and %EF, Table S3: % Enrichment Factor and AUC of VS, Figure S1: The receiver operating characteristic (ROC) curve of MOE on eight proteins, Table S4: Docking scores and rank of selected hits, Figure S2: The chemical structures of compounds 1–30, Table S5: Physicochemical properties of selected hits (1–30), Table S6: Solubility of selected hits (1–30), Table S7: Pharmacokinetic properties of selected hits, Table S8: Drug-likeness and medicinal properties of selected hits (1–30), Table S9: The ADMET results of admetSAR server, Table S10: Interaction analysis of selected hits (1–30), Table S11. Protein–ligand complexes used in re-docking and cross-docking. The structures of compounds in the in-house library are given in SMILE format in the Supplementary Materials.
