# Supplementary material for: In Silico Prediction of Novel Inhibitors of SARS-CoV-2 Main Protease through Structure-Based Virtual Screening and Molecular Dynamic Simulation
Source: Pharmaceuticals (Basel). 2021 Sep 3;14(9):896. doi: 10.3390/ph14090896 (PMC8471057; doi:10.3390/ph14090896)
Supplement: Supplementary file 1 [file pharmaceuticals-14-00896-s001.zip › SI-2-compounds-SMILES.pdf]

	mol	SMILES
1	S60	BrC1=C[C@]2([C@@H](CC[C@@]3(C2C(=O)C=C2[C@@H]4[C@@H](C)[C@@H](CC[C@@]4(CC[C@]23C)C)C)C)[C
2	S11	Br[C@@H]1C[C@@]2(C3[C@](CCC2[C@](C(OCc2ccccc2)=O)(C)C1=O)(C)[C@@]1(CC[C@@]2([C@H](C1=CC3=
3	S61	Br[C@@H]1C[C@]2([C@@H](CC[C@@]3(C2C(=O)C=C2[C@@H]4[C@@H](C)[C@@H](CC[C@@]4(CC[C@]23C)C)C)
4	S12	Br[C@@H]1C[C@]2([C@@H](CC[C@@]3(C2C(=O)C=C2[C@@H]4[C@@H](C)[C@@H](CC[C@@]4(CC[C@]23C)C)C)
5	S14	Br[C@@H]1C[C@]2([C@@H](CC[C@@]3(C2C(=O)C=C2[C@@H]4[C@@H](C)[C@@H](CC[C@@]4(CC[C@]23C)C)C)
6	S13	Br[C@@H]1C[C@]2([C@@H](CC[C@@]3(C2C(=O)C=C2[C@@H]4[C@@H](C)[C@@H](CC[C@@]4(CC[C@]23C)C)C)
7	S46	Br[C@H]1C=C[C@@]([C@@H]2CC[C@@]3(C([C@@]12C)C(=O)C=C1[C@@H]2[C@@H](C)[C@@H](CC[C@@]2(CC[C
8	S42	Br[C@H]1[C@@]([C@@H]2CC[C@@]3(C([C@]2(CC1=O)C)C(=O)C=C1[C@@H]2[C@@H](C)[C@@H](CC[C@@]2(CC
9	S43	Br[C@H]1[C@@]([C@@H]2CC[C@@]3(C([C@]2(C[C@@H]1Br)C)C(=O)C=C1[C@@H]2[C@@H](C)[C@@H](CC[C@@
10	S40	Br[C@H]1[C@@]([C@@H]2CC[C@@]3(C([C@]2(C[C@@H]1O)C)C(=O)C=C1[C@@H]2[C@@H](C)[C@@H](CC[C@@]
11	C-7.mol	Brc1c2c(C(N(CC2)Cc2nnn(c2)-c2cc(OC)c(OC)cc2)C2OCc3c2ccc(OC)c3OC)c(OC)c2OCOc12
12	C-4.mol	Brc1c2c(C(N(CC2)Cc2nnn(c2)-c2ccc(F)cc2)C2OCc3c2ccc(OC)c3OC)c(OC)c2OCOc12
13	N-T-10	Brc1c2c(C(N(CC2)Cc2nnn(c2)-c2ccc(OC)cc2)C2OC(=O)c3c2ccc(OC)c3OC)c(OC)c2OCOc12
14	C-5.mol	Brc1c2c(C(N(CC2)Cc2nnn(c2)-c2ccc(cc2)C)C2OCc3c2ccc(OC)c3OC)c(OC)c2OCOc12
15	C-10.mol	Brc1c2c(C(N(CC2)Cc2nnn(c2)-c2ccccc2)C2OCc3c2ccc(OC)c3OC)c(OC)c2OCOc12
16	N-T-8	Brc1c2c(C(N(CC2)Cc2nnn(c2)CC(O)(C)C)C2OC(=O)c3c2ccc(OC)c3OC)c(OC)c2OCOc12
17	N-T-11	Brc1c2c(C(N(CC2)Cc2nnn(c2)CC(O)COc2ccccc2)C2OC(=O)c3c2ccc(OC)c3OC)c(OC)c2OCOc12
18	C-12.mol	Brc1c2c(C(N(CC2)Cc2nnn(c2)CC(O)COc2ccccc2)C2OCc3c2ccc(OC)c3OC)c(OC)c2OCOc12
19	C-9.mol	Brc1c2c(C(N(CC2)Cc2nnn(c2)CC(O)c2ccccc2)C2OCc3c2ccc(OC)c3OC)c(OC)c2OCOc12
20	C-11.mol	Brc1c2c(C(N(CC2)Cc2nnn(c2)Cc2ccc(Br)cc2)C2OCc3c2ccc(OC)c3OC)c(OC)c2OCOc12
21	N-T-9	Brc1c2c(C(N(CC2)Cc2nnn(c2)Cc2ccc(cc2)C)C2OC(=O)c3c2ccc(OC)c3OC)c(OC)c2OCOc12
22	C-6.mol	Brc1c2c(C(N(CC2)Cc2nnn(c2)Cc2ccccc2)C2OCc3c2ccc(OC)c3OC)c(OC)c2OCOc12
23	TS-109.cdx	Brc1cc(N2/C(/SC=C2c2ccccc2)=N/N=C(\C)/c2cc3OCOc3cc2)ccc1
24	MI-142.mol	Brc1cc(NC(=S)N\N=C(/C)\c2cc(sc2C)C)ccc1
25	MI-22.mol	Brc1cc(NC(=S)N\N=C(/C)\c2cc3c(cc2)cccc3)ccc1
26	MI-102.mol	Brc1cc(NC(=S)N\N=C(/C)\c2nccnc2C)ccc1
27	MI-122.mol	Brc1cc(NC(=S)N\N=C(/C)\c2sc(nc2C)C)ccc1
28	MI-182.mol	Brc1cc(NC(=S)N\N=C\2/CCc3cc(OC)c(OC)cc/23)ccc1
29	MI-202.mol	Brc1cc(NC(=S)N\N=C\c2ccc([N+](=O)[O-])cc2)ccc1
30	RXN-20	Brc1cc(ccc1)-c1n2N=C(CSc2nn1)c1c2c(ccc1)cccc2
31	RXN-22	Brc1cc(ccc1)-c1n2N=C(CSc2nn1)c1cc(Cl)c(Cl)cc1
32	RXN-16.cdx	Brc1cc(ccc1)-c1n2N=C(CSc2nn1)c1ccc(Cl)cc1
33	RXN-17.cdx	Brc1cc(ccc1)-c1n2N=C(CSc2nn1)c1ccc(F)cc1
34	RXN-15.cdx	Brc1cc(ccc1)-c1n2N=C(CSc2nn1)c1ccc(OC)cc1
35	RXN-21	Brc1cc(ccc1)-c1n2N=C(CSc2nn1)c1ccc([N+](=O)[O-])cc1
36	RXN-19	Brc1cc(ccc1)-c1n2N=C(CSc2nn1)c1ccc(cc1)-c1ccccc1
37	RXN-18.cdx	Brc1cc(ccc1)-c1n2N=C(CSc2nn1)c1ccc(cc1)C
38	RXN-28	Brc1cc(ccc1)-c1n2N=C(Sc2nn1)COc1ccc(F)cc1
39	RXN-24	Brc1cc(ccc1)-c1n2N=C(Sc2nn1)COc1ccc(O)cc1
40	RXN-27	Brc1cc(ccc1)-c1n2N=C(Sc2nn1)COc1ccc(OC)cc1
41	RXN-26	Brc1cc(ccc1)-c1n2N=C(Sc2nn1)COc1ccc(cc1)C
42	RXN-25	Brc1cc(ccc1)-c1n2N=C(Sc2nn1)COc1ccccc1C
43	RXN-23	Brc1cc(ccc1)-c1n2N=C(Sc2nn1)COc1ccccc1O
44	N-T-2	Brc1cc(ccc1)Cn1nnc(c1)CN1CCc2c(C1C1OC(=O)c3c1ccc(OC)c3OC)c(OC)c1OCOc1c2
45	R-NPG-7.cdx	Brc1cc(ccc1)\C=C(\N1C(=O)c2c(cccc2)C1=O)/C(O)=O
46	6JA.mol	Brc1cc2C3=NC4=NN(C(N)C4C=C3C(Oc2cc1)NCC1OCCC1)c1ccccc1
47	6KA.mol	Brc1cc2C3=NC4=NN(C(N)C4C=C3C(Oc2cc1)NCc1ccccc1)c1ccc(OC)cc1
48	6GA.mol	Brc1cc2C3=NC4=NN(C(N)C4C=C3C(Oc2cc1)NCc1ccccc1)c1ccccc1
49	4D.mol	Brc1cc2c(OC=C(C3C4C(OC5C3C(=O)CC(C5)(C)C)CC(CC4=O)(C)C)C2=O)cc1
50	4G.mol	Brc1cc2c(OC=C(C3C4C(OC5C3C(=O)CCC5)CCCC4=O)C2=O)cc1
51	F-12	Brc1ccc(-n2nnc(c2)CN2CCc3c(C2C2OCc4c2ccc(OC)c4OC)c(OC)c2OCOc2c3)cc1
52	SB-10.cdx	Brc1ccc(cc1)CC(=O)N\N=C\C(C)c1ccccc1
53	SB-11.cdx	Brc1ccc(cc1)CC(=O)N\N=C\CCCC
54	SB-1.cdx	Brc1ccc(cc1)CC(=O)N\N=C\c1cc(O)c(O)cc1
55	SB-3.cdx	Brc1ccc(cc1)CC(=O)N\N=C\c1cc(OC(=O)C)c(OC)cc1
56	SB-8.cdx	Brc1ccc(cc1)CC(=O)N\N=C\c1cc(OC(=O)C)ccc1
57	SB-14.cdx	Brc1ccc(cc1)CC(=O)N\N=C\c1cc([N+](=O)[O-])ccc1
58	SB-6.cdx	Brc1ccc(cc1)CC(=O)N\N=C\c1ccc(OC(=O)C)cc1
59	SB-15.cdx	Brc1ccc(cc1)CC(=O)N\N=C\c1ccc(OCC)cc1
60	SB-7.cdx	Brc1ccc(cc1)CC(=O)N\N=C\c1ccc(OCC=C)cc1
61	SB-2.cdx	Brc1ccc(cc1)CC(=O)N\N=C\c1ccc(OCc2ccccc2)cc1O

	mol	SMILES
62	SB-13.cdx	Brc1ccc(cc1)CC(=O)N\N=C\c1ccc(cc1)-c1ccccc1
63	SB-9.cdx	Brc1ccc(cc1)CC(=O)N\N=C\c1ccc(cc1)C(C)C
64	SB-5.cdx	Brc1ccc(cc1)CC(=O)N\N=C\c1ccccc1OCC=C
65	SB-4.cdx	Brc1ccc(cc1)CC(=O)N\N=C\c1ccccc1OCc1ccccc1
66	SB-12.cdx	Brc1ccc(cc1)CC(=O)N\N=C\c1ccccc1[N+](=O)[O-]
67	SB-16.cdx	Brc1ccc(cc1)CC(=O)N\N=C\c1occc1
68	N-D-3a	Brc1ccc(cc1)CN1CCc2c(C1C1OC(=O)c3c1ccc(OC)c3OC)c(OC)c1OCOc1c2
69	M-3.cdx	Brc1ccc(cc1)Cc1oc(S)nn1
70	F-14	Brc1ccc(cc1)Cn1nnc(c1)CN1CCc2c(C1C1OCc3c1ccc(OC)c3OC)c(OC)c1OCOc1c2
71	KES-5.cdx	Brc1ccccc1C(OCC(=O)c1cc(Cl)c(Cl)cc1)=O
72	KES-7.cdx	Brc1ccccc1C(OCC(=O)c1cc2c(cc1)cccc2)=O
73	KES-1.cdx	Brc1ccccc1C(OCC(=O)c1ccc(Cl)cc1)=O
74	KES-4.cdx	Brc1ccccc1C(OCC(=O)c1ccc(F)cc1)=O
75	KES-3.cdx	Brc1ccccc1C(OCC(=O)c1ccc(OC)cc1)=O
76	KES-6.cdx	Brc1ccccc1C(OCC(=O)c1ccc([N+](=O)[O-])cc1)=O
77	KES-8.cdx	Brc1ccccc1C(OCC(=O)c1ccc(cc1)-c1ccccc1)=O
78	KES-2.cdx	Brc1ccccc1C(OCC(=O)c1ccc(cc1)C)=O
79	MI-84.mol	Brc1sc(cc1)/C(=N/NC(=S)N)/C
80	MI-99.mol	Brc1sc(cc1)/C(=N/NC(=S)NC(=O)c1ccccc1)/C
81	MI-90.mol	Brc1sc(cc1)/C(=N/NC(=S)NC1CCCCC1)/C
82	MI-86.mol	Brc1sc(cc1)/C(=N/NC(=S)Nc1c2c(ccc1)cccc2)/C
83	MI-91.mol	Brc1sc(cc1)/C(=N/NC(=S)Nc1cc(Cl)ccc1)/C
84	MI-93.mol	Brc1sc(cc1)/C(=N/NC(=S)Nc1ccc(F)cc1)/C
85	MI-83.mol	Brc1sc(cc1)/C(=N/NC(=S)Nc1ccc([N+](=O)[O-])cc1)/C
86	MI-100.mol	Brc1sc(cc1)/C(=N/NC(=S)Nc1ccc(cc1)CC)/C
87	MI-92.mol	Brc1sc(cc1)/C(=N/NC(=S)Nc1ccccc1)/C
88	10-Cembrene	C1CC(=CCCC(=CCC=C(C=CC1C(C)C)C)C)C
89	4-Cembrene-A	C1CC(=CCCC(=CCC=C(CCC1C(C)=C)C)C)C
90	6-Cembrene-C	C1CC(=CCCC(=CCCC(=CC=C1C(C)C)C)C)C
91	11-Isocembrene	C1CC(=CCCC(=CCCC(C=CC1C(C)=C)=C)C)C
92	5-Isocembrene	C1CC(=CCCC(=CCCC(C=CC1C(C)C)=C)C)C
93	6BA.mol	ClC(\C=C/1\C2=NC3=NN(C(N)C3C=C2C(OC\1)NCc1ccccc1)c1ccccc1)=C
94	S45	Cl[C@H]1C=C[C@@]([C@@H]2CC[C@@]3(C([C@@]12C)C(=O)C=C1[C@@H]2[C@@H](C)[C@@H](CC[C@@]2(CC[C
95	8.cdx	Clc1c2c(sc1C(=O)Nc1ccccc1)c1sc(C(=O)Nc3ccccc3)c(Cl)c1cc2
96	2.cdx	Clc1c2c(sc1C(Cl)=O)c1sc(C(Cl)=O)c(Cl)c1cc2
97	3.cdx	Clc1c2c(sc1C(OCCCC)=O)c1sc(C(OCCCC)=O)c(Cl)c1cc2
98	N-T-1	Clc1cc(-n2nnc(c2)CN2CCc3c(C2C(OC)c2ccc(OC)c(OC)c2C=O)c(OC)c2OCOc2c3)ccc1Cl
99	F-17.mol	Clc1cc(-n2nnc(c2)CN2CCc3c(C2C2OCc4c2ccc(OC)c4OC)c(OC)c2OCOc2c3)ccc1Cl
100	A5D.mol	Clc1cc(Cl)ccc1C(N1CCc2c([C@@H]1[C@H]1OCc3c1ccc(OC)c3OC)c(OC)c1OCOc1c2)C(=O)N
101	M-8.cdx	Clc1cc(Cl)ccc1Cc1oc(S)nn1
102	TT-6.cdx	Clc1cc(F)c(F)cc1C=1Sc2n(N=1)c(nn2)-c1ccncc1
103	FT-8.cdx	Clc1cc(F)c(F)cc1C=1Sc2n(N=1)c(nn2)-c1occc1
104	M-17.cdx	Clc1cc(F)c(cc1)-c1oc(S)nn1
105	NT-1.cdx	Clc1cc(F)c(cc1)C=1Sc2n(N=1)c(nn2)-c1cccnc1
106	TT-1.cdx	Clc1cc(F)c(cc1)C=1Sc2n(N=1)c(nn2)-c1ccncc1
107	FT-1.cdx	Clc1cc(F)c(cc1)C=1Sc2n(N=1)c(nn2)-c1occc1
108	MI-151.mol	Clc1cc(NC(=S)N\N=C(/C)\c2cc(sc2C)C)ccc1
109	MI-31.mol	Clc1cc(NC(=S)N\N=C(/C)\c2cc3c(cc2)cccc3)ccc1
110	MI-111.mol	Clc1cc(NC(=S)N\N=C(/C)\c2nccnc2C)ccc1
111	MI-131.mol	Clc1cc(NC(=S)N\N=C(/C)\c2sc(nc2C)C)ccc1
112	MI-191.mol	Clc1cc(NC(=S)N\N=C\2/CCc3cc(OC)c(OC)cc/23)ccc1
113	MI-211.mol	Clc1cc(NC(=S)N\N=C\c2ccc([N+](=O)[O-])cc2)ccc1
114	R-NPG-20.cdx	Clc1cc(O)cc(Cl)c1\C=C(\N1C(=O)c2c(cccc2)C1=O)/C(O)=O
115	I-2.cdx	Clc1cc(ccc1)C1=Nn2c(SC1)nnc2-c1ccncc1
116	R-NPG-8.cdx	Clc1cc(ccc1)\C=C(\N1C(=O)c2c(cccc2)C1=O)/C(O)=O
117	IN-3CB.cdx	Clc1cc(ccc1)\C=N/Nc1sc2c(n1)cccc2
118	17.cdx	Clc1cc(ccc1Cl)-c1c2c(sc1C(OCCCC)=O)c1sc(C(OCCCC)=O)c(c1cc2)-c1cc(Cl)c(Cl)cc1
119	RXN-8.cdx	Clc1cc(ccc1Cl)C1=Nn2c(SC1)nnc2-c1ccc(OC)cc1
120	N-8.cdx	Clc1cc(ccc1Cl)C1=Nn2c(SC1)nnc2-c1cccnc1
121	I-8.cdx	Clc1cc(ccc1Cl)C1=Nn2c(SC1)nnc2-c1ccncc1
122	F-8.cdx	Clc1cc(ccc1Cl)C1=Nn2c(SC1)nnc2-c1occc1

	mol	SMILES
123	M-5.cdx	Clc1cc(ccc1Cl)Cc1oc(S)nn1
124	KES-13.cdx	Clc1cc(ccc1F)C(OCC(=O)c1cc(Cl)c(Cl)cc1)=O
125	KES-15.cdx	Clc1cc(ccc1F)C(OCC(=O)c1cc2c(cc1)cccc2)=O
126	KES-10.cdx	Clc1cc(ccc1F)C(OCC(=O)c1ccc(Cl)cc1)=O
127	KES-9.cdx	Clc1cc(ccc1F)C(OCC(=O)c1ccc(F)cc1)=O
128	KES-12.cdx	Clc1cc(ccc1F)C(OCC(=O)c1ccc(OC)cc1)=O
129	KES-14.cdx	Clc1cc(ccc1F)C(OCC(=O)c1ccc([N+](=O)[O-])cc1)=O
130	KES-16.cdx	Clc1cc(ccc1F)C(OCC(=O)c1ccc(cc1)-c1ccccc1)=O
131	KES-11.cdx	Clc1cc(ccc1F)C(OCC(=O)c1ccc(cc1)C)=O
132	NT-2.cdx	Clc1cc(ccc1F)C=1Sc2n(N=1)c(nn2)-c1cccnc1
133	TT-2.cdx	Clc1cc(ccc1F)C=1Sc2n(N=1)c(nn2)-c1ccncc1
134	FT-2.cdx	Clc1cc(ccc1F)C=1Sc2n(N=1)c(nn2)-c1occc1
135	6CA.mol	Clc1cc2C3=NC4=NN(C(N)C4C=C3C(Oc2cc1)NCCc1ccccc1)c1ccccc1
136	4C.mol	Clc1cc2c(OC=C(C3C4C(OC5C3C(=O)CCC5)CCCC4=O)C2=O)cc1
137	4H.mol	Clc1cc2c(OC=C(C3C4C(OC=C3C(=O)CCC)CC(CC4=O)(C)C)C2=O)cc1
138	Chloroquine.cdx	Clc1cc2nccc(NC(CCCN(CC)CC)C)c2cc1
139	MI-181.mol	Clc1ccc(NC(=S)N\N=C\2/CCc3cc(OC)c(OC)cc/23)cc1
140	MI-201.mol	Clc1ccc(NC(=S)N\N=C\c2ccc([N+](=O)[O-])cc2)cc1
141	7D.mol	Clc1ccc(NC2Oc3c(C4=NC(N=Nc5ccccc5)C(C=C24)C#N)cccc3)cc1
142	25.cdx	Clc1ccc(cc1)-c1c2c(sc1C(OCCCC)=O)c1sc(C(OCCCC)=O)c(c1cc2)-c1ccc(Cl)cc1
143	RXN-2.cdx	Clc1ccc(cc1)C1=Nn2c(SC1)nnc2-c1ccc(OC)cc1
144	N-2.cdx	Clc1ccc(cc1)C1=Nn2c(SC1)nnc2-c1cccnc1
145	F-2.cdx	Clc1ccc(cc1)C1=Nn2c(SC1)nnc2-c1occc1
146	M-10.cdx	Clc1ccc(cc1)Cc1oc(S)nn1
147	R-NPG-13.cdx	Clc1ccc(cc1)\C=C(\N1C(=O)c2c(cccc2)C1=O)/C(O)=O
148	IN-4-CB.cdx	Clc1ccc(cc1)\C=N/Nc1sc2c(n1)cccc2
149	F-13	Clc1cccc(Cl)c1-n1nnc(c1)CN1CCc2c(C1C1OCc3c1ccc(OC)c3OC)c(OC)c1OCOc1c2
150	M-16.cdx	Clc1cccc(Cl)c1Cc1oc(S)nn1
151	S-4.mol	Clc1ccccc1C(N1CCc2c([C@@H]1[C@H]1OCc3c1ccc(OC)c3OC)c(OC)c1OCOc1c2)C#N
152	A4D.mol	Clc1ccccc1C(N1CCc2c([C@@H]1[C@H]1OCc3c1ccc(OC)c3OC)c(OC)c1OCOc1c2)C(=O)N
153	A4U.mol	Clc1ccccc1C(N1CCc2c([C@@H]1[C@H]1OCc3c1ccc(OC)c3OC)c(OC)c1OCOc1c2)C(=O)N
154	MI-36.mol	Clc1ccccc1CNC(=S)N\N=C(/C)\c1cc2c(cc1)cccc2
155	M-11.cdx	Clc1ccccc1Cc1oc(S)nn1
156	R-NPG-22.cdx	Clc1ccccc1\C=C(\N1C(=O)c2c(cccc2)C1=O)/C(O)=O
157	6EA.mol	Fc1cc2C3=NC4=NN(C(N)C4C=C3C(Oc2cc1)NCCc1ccccc1)c1ccccc1
158	6FA.mol	Fc1cc2C3=NC4=NN(C(N)C4C=C3C(Oc2cc1)NCc1ccccc1)c1ccccc1
159	4F.mol	Fc1cc2c(OC=C(C3C4C(OC5C3C(=O)CC(C5)(C)C)CC(CC4=O)(C)C)C2=O)cc1
160	4E.mol	Fc1cc2c(OC=C(C3C4C(OC5C3C(=O)CCC5)CCCC4=O)C2=O)cc1
161	F-8	Fc1ccc(-n2nnc(c2)CN2CCc3c(C2C2OCc4c2ccc(OC)c4OC)c(OC)c2OCOc2c3)cc1
162	6HA.mol	Fc1ccc(N2N=C3N=C4C(=CC3C2N)C(Oc2c4cccc2)NC2CCCCC2)cc1
163	6IA.mol	Fc1ccc(N2N=C3N=C4C(=CC3C2N)C(Oc2c4cccc2)NCc2ccccc2)cc1
164	N-D-12b	Fc1ccc(cc1)C(CN1CCc2c(C1C(O)c1ccc(OC)c(OC)c1CO)c(OC)c1OCOc1c2)c1ccc(F)cc1
165	N-D-12a	Fc1ccc(cc1)C(CN1CCc2c(C1C1OC(=O)c3c1ccc(OC)c3OC)c(OC)c1OCOc1c2)c1ccc(F)cc1
166	S-5.mol	Fc1ccc(cc1)C(N1CCc2c([C@@H]1[C@H]1OCc3c1ccc(OC)c3OC)c(OC)c1OCOc1c2)C#N
167	S-6D.mol	Fc1ccc(cc1)C(N1CCc2c([C@@H]1[C@H]1OCc3c1ccc(OC)c3OC)c(OC)c1OCOc1c2)C#N
168	A6D.mol	Fc1ccc(cc1)C(N1CCc2c([C@@H]1[C@H]1OCc3c1ccc(OC)c3OC)c(OC)c1OCOc1c2)C(=O)N
169	A6U.mol	Fc1ccc(cc1)C(N1CCc2c([C@@H]1[C@H]1OCc3c1ccc(OC)c3OC)c(OC)c1OCOc1c2)C(=O)N
170	AM6.mol	Fc1ccc(cc1)C(N1CCc2c([C@@H]1[C@H]1OCc3c1ccc(OC)c3OC)c(OC)c1OCOc1c2)CN
171	N-D-27a	Fc1ccc(cc1)CN1CCc2c(C1C1OC(=O)c3c1ccc(OC)c3OC)c(OC)c1OCOc1c2
172	N-T-3	Fc1ccc(cc1)Cn1nnc(c1)CN1CCc2c(C1C1OC(=O)c3c1ccc(OC)c3OC)c(OC)c1OCOc1c2
173	F-2	Fc1ccc(cc1)Cn1nnc(c1)CN1CCc2c(C1C1OCc3c1ccc(OC)c3OC)c(OC)c1OCOc1c2
174	Favipiravir	Fc1nc(C(=O)N)c(O)nc1
175	S37	I[C@H]1[C@@]([C@@H]2CC[C@@]3(C([C@]2(C[C@@H]1OC(=O)C)C)C(=O)C=C1[C@@H]2[C@@H](C)[C@@H](CC
176	N11.mol	N(=C(/N)\c1ccccc1)/c1ccccc1CCC
177	16-Duva-3,9,13-triene-1,5-?-diol	O(C(=O)C)C1(CC=CC(O)(CCC=C(CCC(C=C1)C(C)C)C)C)C
178	39-3-O-acetyl-9,11-dehydro-b- boswell	O(C(=O)C)C1CCC2(C(CCC3(C2=CC=C2C4C(C)C(CCC4(CCC23C)C)C)C)C1(C(O)=O)C)C
179	41-3-O-acetyl-9,11-dehydro-?- boswell	O(C(=O)C)C1CCC2(C(CCC3(C2=CC=C2C4CC(CCC4(CCC23C)C)(C)C)C)C1(C(O)=O)C)C
180	30-3-?-acetyl-b-Boswellic acid	O(C(=O)C)C1CCC2(C(CCC3(C2CC=C2C4C(C)C(CCC4(CCC23C)C)C)C)C1(C(O)=O)C)C
181	33-3- ? -acetyl-?-Boswellic acid	O(C(=O)C)C1CCC2(C(CCC3(C2CC=C2C4CC(CCC4(CCC23C)C)(C)C)C)C1(C(O)=O)C)C
182	31-3-?-acetyl-11-keto-b-Boswellic aci	O(C(=O)C)C1CCC2(C3C(CCC2C1(C(O)=O)C)(C)C1(C(C2C(C)C(CCC2(CC1)C)C)=CC3=O)C)C
183	43-3-acetyl-11-hydroxy-12-ene-24-oic	O(C(=O)C)C1CCC2(C3C(CCC2C1(C(O)=O)C)(C)C1(C(C2C(C)C(CCC2(CC1)C)C)=CC3O)C)C

	mol	SMILES
184	42-3a-acetoxyurs-5:12-dien-24-oic aci	O(C(=O)C)C1CCC2(C3CC=C4C5C(C)C(CCC5(CCC4(C)C3(CC=C2C1(C(O)=O)C)C)C)C)C
185	3a-acetoxyurs-5:12-dien-24-oic acid	O(C(=O)C)[C@@H]1CC[C@@]2(C3CC=C4C5[C@@H](C)[C@@H](CC[C@@]5(CC[C@@]4(C)[C@@]3(CC=C2[C@]1(C
186	3-O-acetyl-9,11-dehydro-b-Boswellic a	O(C(=O)C)[C@@H]1CC[C@]2(C(CC[C@@]3(C2=CC=C2C4[C@@H](C)[C@@H](CC[C@@]4(CC[C@]23C)C)C)C)[C@
187	Alfa-Amyrin Acetae.mol	O(C(=O)C)[C@@H]1CC[C@]2(C(CC[C@@]3(C2C=CC2[C@@H]4[C@@H](C)[C@@H](CC[C@@]4(CC[C@]23C)C)C)C
188	Acetyl-Boswellic acid-b-ABA	O(C(=O)C)[C@@H]1CC[C@]2(C(CC[C@@]3(C2CC=C2C4[C@@H](C)[C@@H](CC[C@@]4(CC[C@]23C)C)C)C)[C@]
189	DCMM100.mol	O(C(=O)C)[C@@H]1CC[C@]2(C(CC[C@@]3(C2CC=C2C4[C@@H](C)[C@@H](CC[C@@]4(CC[C@]23C)C)C)C)[C@]
190	3a-acetoxylup-12:20(29)-dien-24-oic a	O(C(=O)C)[C@@H]1CC[C@]2(C(CC[C@@]3(C2CC=C2C4[C@@](CC[C@]23C)(CC[C@H]4C(C)=C)C)C)[C@]1(C(O
191	a-ABA	O(C(=O)C)[C@@H]1CC[C@]2(C(CC[C@@]3(C2CC=C2[C@@H]4CC(CC[C@@]4(CC[C@]23C)C)(C)C)C)[C@]1(C(O
192	BETA-amyrine acetate	O(C(=O)C)[C@H]1CC[C@]2(C(CC[C@@]3(C2CC=C2C4[C@@H](C)[C@@H](CC[C@@]4(CC[C@]23C)C)C)C)C1(C)
193	ß-amyrine acetate.mol	O(C(=O)C)[C@H]1CC[C@]2(C(CC[C@@]3(C2CC=C2C4[C@@H](C)[C@@H](CC[C@@]4(CC[C@]23C)C)C)C)C1(C)
194	3-epi-a-ABA	O(C(=O)C)[C@H]1CC[C@]2(C(CC[C@@]3(C2CC=C2[C@@H]4CC(CC[C@@]4(CC[C@]23C)C)(C)C)C)[C@]1(C(O)
195	3-epi-b-ABA	O(C(=O)C)[C@H]1CC[C@]2(C(CC[C@@]3(C2CC=C2[C@@H]4[C@@H](C)[C@@H](CC[C@@]4(CC[C@]23C)C)C)C)
196	3a-Acetoxylup-12-20(29).mol	O(C(=O)CC(=O)C)[C@@H]1CC[C@]2(C(CC[C@@]3(C2CCC2C4C(CC[C@]23C)CC[C@H]4C(=O)C)C)[C@]1(C(O)=
197	SF-10-2	O(C(=O)CCCCCCCCCCCCCCCCCCCC)CCC
198	Oseltamivir	O(C(CC)CC)C1C=C(CC(N)C1NC(=O)C)C(OCC)=O
199	AJAOMD2	O(C)C1=CC(=O)C(OC)=CC1=O
200	AJAOMD1	O(C)C1=CC(=O)C=C(OC)C1=O
201	36-11-?-methoxy-b-boswellic acid	O(C)C1C2C(CCC3C(C(O)=O)(C)C(O)CCC23C)(C)C2(C(C3C(C)C(CCC3(CC2)C)C)=C1)C
202	BEMD-12	O(C)C1[C@H](O)[C@@H](O)C(O)[C@H](O)[C@H]1O
203	Quebratichol.mol	O(C)C1[C@H](O)[C@@H](O)C(O)[C@H](O)[C@H]1O
204	U-16.mol	O(C)c1cc(N(C(C(=O)NC(C)(C)C)c2ccc(OC)cc2)C(=O)CCC(=O)N2CCC(N(C(=O)CC)c3ccccc3)(CC2)COC)cc
205	R-NPG-2.cdx	O(C)c1cc(ccc1O)\C=C(\N1C(=O)c2c(cccc2)C1=O)/C(O)=O
206	U-13.mol	O(C)c1cc(ccc1OC)C(N(C(=O)CCC(=O)N1CCC(N(C(=O)CC)c2ccccc2)(CC1)COC)c1cc(OC)c(OC)cc1)C(=O)N
207	R-NPG-15.cdx	O(C)c1cc(ccc1OC)\C=C(\N1C(=O)c2c(cccc2)C1=O)/C(O)=O
208	R-NPG-4.cdx	O(C)c1ccc(OC)cc1\C=C(\N1C(=O)c2c(cccc2)C1=O)/C(O)=O
209	SBEH-17	O(C)c1ccc(cc1)C=O
210	R-NPG-19.cdx	O(C)c1ccc(cc1)\C=C(\N1C(=O)c2c(cccc2)C1=O)/C(O)=O
211	R-NPG-25.cdx	O(C)c1cccc(C)c1\C=C(\N1C(=O)c2c(cccc2)C1=O)/C(O)=O
212	R-NPG-10.cdx	O(C)c1ccccc1\C=C(\N1C(=O)c2c(cccc2)C1=O)/C(O)=O
213	R-NPG-26.cdx	O(C)c1ccccc1\C=C(\N1C(=O)c2c(cccc2)C1=O)/C(O)=O
214	Lopinavir	O(CC(=O)NC(Cc1ccccc1)C(O)CC(NC(=O)C(N1CCCNC1=O)C(C)C)Cc1ccccc1)c1c(cccc1C)C
215	37-11-?-ethoxy-b-boswellic acid	O(CC)C1C2C(CCC3C(C(O)=O)(C)C(O)CCC23C)(C)C2(C(C3C(C)C(CCC3(CC2)C)C)=C1)C
216	11a-Ethoxy-ß-boswellic Acid.mol	O(CC)[C@H]1C2[C@](CCC3[C@](C(O)=O)(C)[C@H](O)CC[C@]23C)(C)[C@]2(C(C3[C@@H](C)[C@@H](CC[C@
217	U-6.mol	O(CC1(N(C(=O)CC)c2ccccc2)CCN(CC1)C(=O)CCC(=O)N(C(C(=O)NC(C)(C)C)c1ccccc1)c1ccccc1)C
218	U-14.mol	O(CC1(N(C(=O)CC)c2ccccc2)CCN(CC1)C(=O)CCC(=O)N(C(C(CC)C)C(=O)NC1CCCCC1)c1ccc(cc1)C)C
219	U-5.mol	O(CC1(N(C(=O)CC)c2ccccc2)CCN(CC1)C(=O)CCC(=O)N(Cc1ccccc1)C(C(CC)C)C(=O)NC(C)(C)C)C
220	U-15.mol	O(CC1(N(C(=O)CC)c2ccccc2)CCN(CC1)C(=O)CCC(=O)N(Cc1ccccc1)C(C(CC)C)C(=O)NC(C)(C)C)C
221	U-17.mol	O(CC1(N(C(=O)CC)c2ccccc2)CCN(CC1)C(=O)CCC(=O)N(Cc1ccccc1)C(CC)C(=O)NC(C)(C)C)C
222	5.cdx	O(C\C=C(\C)/C)C=1c2c(NC(=O)C=1CC(O)C(C)=C)cccc2
223	10.cdx	O(C\C=C(\C)/C)C=1c2c(NC(=O)C=1C\C=C(\C)/C)cccc2
224	SBME-P5	O1C(C(O)C(O)C(O)C1COC(=O)\C=C\c1ccc(O)cc1)c1c(c2c(OC(=CC2=O)CC(=O)C)cc1O)C
225	SBME-P3	O1C(C(O)C(O)C(O)C1COC(=O)\C=C\c1ccc(O)cc1)c1c(c2c(OC(=CC2=O)CC(=O)C)cc1OC)C
226	S5-5	O1C(C(O)C(O)C(O)C1COC(=O)\C=C\c1ccc(O)cc1)c1c2OC(=CC(=O)c2c(cc1O)C)CC(=O)C
227	QTME-16	O1C(C)C(O)C(O)C(O)C1OCc1cc(O)c2c(c1)C(=O)c1c(C2=O)c(O)cc(O)c1
228	SBEH-60	O1C(C)C(O)C(O)C(O)C1OCc1cc(O)c2c(c1)C(=O)c1c(C2=O)c(O)cc(O)c1
229	14.cdx	O1C(CCC(OC(=O)C)C(OC(=O)C)(C)C)(C)C1COc1ccc(cc1)CCNC(=O)c1ccccc1
230	13.cdx	O1C(CCC(OC(=O)C)C(OC(=O)C)(C)C)(C)C1Oc1ccc(cc1)CCNC(O)\C(=C(/C)\C)\C
231	QTME-14	O1C(CO)C(O)C(O)C(O)C1OCc1c2c(cc(O)c1)C(=O)c1c(C2=O)c(O)cc(c1)CO
232	QTME-12	O1C(CO)C(O)C(O)C(O)C1Oc1c2c(cc(O)c1)C(=O)c1c(C2=O)c(O)cc(c1)C
233	QTEM-10	O1C(CO)C(O)C(O)C(OC(=O)c2cc(O)c(O)c(O)c2)C1OC(=O)\C=C\c1cc(O)c(O)cc1
234	N-D-1a	O1C(c2c(c(OC)c(OC)cc2)C1=O)C1N(CCc2c1c(OC)c1OCOc1c2)CC1CC1
235	N-D-25a	O1C(c2c(c(OC)c(OC)cc2)C1=O)C1N(CCc2c1c(OC)c1OCOc1c2)CCCC
236	N-D-19a	O1C(c2c(c(OC)c(OC)cc2)C1=O)C1N(CCc2c1c(OC)c1OCOc1c2)CCOCCOCCCC
237	N-D-15a	O1C(c2c(c(OC)c(OC)cc2)C1=O)C1N(CCc2c1c(OC)c1OCOc1c2)CCOc1ccccc1
238	N-D-18a	O1C(c2c(c(OC)c(OC)cc2)C1=O)C1N(CCc2c1c(OC)c1OCOc1c2)Cc1c2OCOc2ccc1
239	N-D-11a	O1C(c2c(c(OC)c(OC)cc2)C1=O)C1N(CCc2c1c(OC)c1OCOc1c2)Cc1c2c(cc3c1cccc3)cccc2
240	N-D-24a	O1C(c2c(c(OC)c(OC)cc2)C1=O)C1N(CCc2c1c(OC)c1OCOc1c2)Cc1ccc(OC)cc1
241	N-D-10a	O1C(c2c(c(OC)c(OC)cc2)C1=O)C1N(CCc2c1c(OC)c1OCOc1c2)Cc1ccc(cc1)C(C)(C)C
242	N-T-7	O1C(c2c(c(OC)c(OC)cc2)C1=O)C1N(CCc2c1c(OC)c1OCOc1c2)Cc1nnn(c1)CC(O)CC
243	N-T-6	O1C(c2c(c(OC)c(OC)cc2)C1=O)C1N(CCc2c1c(OC)c1OCOc1c2)Cc1nnn(c1)CC(O)COCC=C
244	N-T-5	O1C(c2c(c(OC)c(OC)cc2)C1=O)C1N(CCc2c1c(OC)c1OCOc1c2)Cc1nnn(c1)CC(O)COCCCC

	mol	SMILES
245	N-T-4	O1C(c2c(c(OC)c(OC)cc2)C1=O)C1N(CCc2c1c(OC)c1OCOc1c2)Cc1nnn(c1)CC(O)c1ccccc1
246	6DA.mol	O1C/C(/C2=NC3=NN(C(N)C3C=C2C1NC1CCCCC1)c1ccccc1)=C\C=C
247	3.cdx	O1C2(C1C(C(O)(C)C)C\C=C(\C)/C)C(=O)c1c(NC2=O)cccc1
248	19-Boscartin-C	O1C2(CCC1(C1OC(CC1)(C)C(O)CC(=O)C(=CC2)C)C)C(C)C
249	18-Boscartin-B	O1C2(CCC1(C1OC(CC1)(C)C(O)CC1OC1(CC2)C)C)C(C)C
250	9-Isoincensole-Oxide	O1C2(CCC1(C1OC(CC1)(C)C(O)CCC(=CC2)C)C)C(C)C
251	26-Incensfuran	O1C2(CCC1(C1OC(CC1)(CCCC(=CC2)C)C)C)C(C)C
252	17-Boscartin-A	O1C2(CCC1(CC(O)C1(OC(CC1)C(=CCC2O)C)C)C(C)C)C
253	22-Boscartin-F	O1C2(CCC1(CC1OC1(CC(O)C=C(C)C(=O)CC2O)C)C(C)C)C
254	20--Boscartin-D	O1C2(CCC1(CC1OC1(CC(O)C=C(CCC2O)C)C)C(C)C)C
255	23-Boscartin-G	O1C2(CCC1(CC1OC1(CCC=C(C)C(=O)CC2O)C)C(C)C)C
256	24-Boscartin-H	O1C2(CCC1(CC1OC1(CCC=C(C)C(=O)CC2O)C)C(C)C)C
257	7-Incensole-Oxide	O1C2(CCC1(CC1OC1(CCC=C(CCC2O)C)C)C(C)C)C
258	8-Incensole-Oxide-Acetate	O1C2(CCC1(CC1OC1(CCC=C(CCC2OC(=O)C)C)C)C(C)C)C
259	21-Boscartin-E	O1C2(CCC1(CC1OC1(CCC=C(OC)CCC2O)C)C(C)C)C
260	27-Isoincensolol	O1C2(CCC1(CC=C(CCC(O)C(O)(CCC2O)C)C)C(C)C)C
261	2-Incensole	O1C2(CCC1(CCC(=CCCC(=CCC2O)C)C)C(C)C)C
262	3-Incensole_Acetate	O1C2(CCC1(CCC(=CCCC(=CCC2OC(=O)C)C)C)C(C)C)C
263	4.cdx	O1C2=C(C=CC1(C)C)C(=O)Nc1c2cccc1
264	4B.mol	O1C2C(C(C3C1CC(CC3=O)(C)C)C1=COc3c(cccc3)C1=O)C(=O)CC(C2)(C)C
265	4A.mol	O1C2C(C(C3C1CCCC3=O)C1=COc3c(cccc3)C1=O)C(=O)CCC2
266	14-Duva-3-9-13-triene-1-?-hydroxy-5-8	O1C2CCC1(C=CCC(OC(=O)C)(C=CC(CC=C2C)C(C)C)C)C
267	S64	O1C[C@]12CC[C@]1([C@@H](CC[C@@]3(C1C(=O)C=C1[C@@H]4[C@@H](C)[C@@H](CC[C@@]4(CC[C@]13C)C)C
268	F-18.mol	O1Cc2c(ccc(OC)c2OC)C1C1N(CCc2c1c(OC)c1OCOc1c2)Cc1nnn(c1)-c1cc(OC)c(OC)cc1
269	F-4.mol	O1Cc2c(ccc(OC)c2OC)C1C1N(CCc2c1c(OC)c1OCOc1c2)Cc1nnn(c1)-c1ccc(OC)cc1
270	F-3.mol	O1Cc2c(ccc(OC)c2OC)C1C1N(CCc2c1c(OC)c1OCOc1c2)Cc1nnn(c1)-c1ccc(cc1)C
271	F-11.mol	O1Cc2c(ccc(OC)c2OC)C1C1N(CCc2c1c(OC)c1OCOc1c2)Cc1nnn(c1)-c1ccc(cc1)CC
272	F-9	O1Cc2c(ccc(OC)c2OC)C1C1N(CCc2c1c(OC)c1OCOc1c2)Cc1nnn(c1)-c1ccccc1
273	F-23.mol	O1Cc2c(ccc(OC)c2OC)C1C1N(CCc2c1c(OC)c1OCOc1c2)Cc1nnn(c1)CC(O)C
274	F-16.mol	O1Cc2c(ccc(OC)c2OC)C1C1N(CCc2c1c(OC)c1OCOc1c2)Cc1nnn(c1)CC(O)CC
275	F-22.mol	O1Cc2c(ccc(OC)c2OC)C1C1N(CCc2c1c(OC)c1OCOc1c2)Cc1nnn(c1)CC(O)COC(C)C
276	F-15.mol	O1Cc2c(ccc(OC)c2OC)C1C1N(CCc2c1c(OC)c1OCOc1c2)Cc1nnn(c1)CC(O)COCC=C
277	F-19.mol	O1Cc2c(ccc(OC)c2OC)C1C1N(CCc2c1c(OC)c1OCOc1c2)Cc1nnn(c1)CC(O)COCCCC
278	F-21.mol	O1Cc2c(ccc(OC)c2OC)C1C1N(CCc2c1c(OC)c1OCOc1c2)Cc1nnn(c1)CC(O)COc1ccccc1
279	F-6.mol	O1Cc2c(ccc(OC)c2OC)C1C1N(CCc2c1c(OC)c1OCOc1c2)Cc1nnn(c1)CC(O)c1ccccc1
280	F-7.mol	O1Cc2c(ccc(OC)c2OC)C1C1N(CCc2c1c(OC)c1OCOc1c2)Cc1nnn(c1)Cc1ccc(cc1)C
281	F-1.mol	O1Cc2c(ccc(OC)c2OC)C1C1N(CCc2c1c(OC)c1OCOc1c2)Cc1nnn(c1)Cc1ccccc1
282	S-3.mol	O1Cc2c(ccc(OC)c2OC)[C@H]1[C@@H]1N(CCc2c1c(OC)c1OCOc1c2)C(C#N)c1cc(OC)c(OC)cc1
283	S-12.mol	O1Cc2c(ccc(OC)c2OC)[C@H]1[C@@H]1N(CCc2c1c(OC)c1OCOc1c2)C(C#N)c1cc2Cc3c(-c2cc1)cccc3
284	S-12U.mol	O1Cc2c(ccc(OC)c2OC)[C@H]1[C@@H]1N(CCc2c1c(OC)c1OCOc1c2)C(C#N)c1cc2Cc3c(-c2cc1)cccc3
285	S-7.mol	O1Cc2c(ccc(OC)c2OC)[C@H]1[C@@H]1N(CCc2c1c(OC)c1OCOc1c2)C(C#N)c1ccc(OC)cc1OC
286	S-11.mol	O1Cc2c(ccc(OC)c2OC)[C@H]1[C@@H]1N(CCc2c1c(OC)c1OCOc1c2)C(C#N)c1ccc(OCc2ccccc2)cc1
287	S-16.mol	O1Cc2c(ccc(OC)c2OC)[C@H]1[C@@H]1N(CCc2c1c(OC)c1OCOc1c2)C(C#N)c1ccc(cc1)C
288	S-16U.mol	O1Cc2c(ccc(OC)c2OC)[C@H]1[C@@H]1N(CCc2c1c(OC)c1OCOc1c2)C(C#N)c1ccc(cc1)C
289	S-9.mol	O1Cc2c(ccc(OC)c2OC)[C@H]1[C@@H]1N(CCc2c1c(OC)c1OCOc1c2)C(C#N)c1ccc(cc1)C(C)C
290	S-1.mol	O1Cc2c(ccc(OC)c2OC)[C@H]1[C@@H]1N(CCc2c1c(OC)c1OCOc1c2)C(C#N)c1ccccc1
291	S-15.mol	O1Cc2c(ccc(OC)c2OC)[C@H]1[C@@H]1N(CCc2c1c(OC)c1OCOc1c2)C(C#N)c1ccccc1O
292	S-8.mol	O1Cc2c(ccc(OC)c2OC)[C@H]1[C@@H]1N(CCc2c1c(OC)c1OCOc1c2)C(C#N)c1cccnc1
293	A3D.mol	O1Cc2c(ccc(OC)c2OC)[C@H]1[C@@H]1N(CCc2c1c(OC)c1OCOc1c2)C(C(=O)N)c1cc(OC)c(OC)cc1
294	A3U.mol	O1Cc2c(ccc(OC)c2OC)[C@H]1[C@@H]1N(CCc2c1c(OC)c1OCOc1c2)C(C(=O)N)c1cc(OC)c(OC)cc1
295	A2D.mol	O1Cc2c(ccc(OC)c2OC)[C@H]1[C@@H]1N(CCc2c1c(OC)c1OCOc1c2)C(C(=O)N)c1cc(Oc2ccccc2)ccc1
296	A2U.mol	O1Cc2c(ccc(OC)c2OC)[C@H]1[C@@H]1N(CCc2c1c(OC)c1OCOc1c2)C(C(=O)N)c1cc(Oc2ccccc2)ccc1
297	A12D.mol	O1Cc2c(ccc(OC)c2OC)[C@H]1[C@@H]1N(CCc2c1c(OC)c1OCOc1c2)C(C(=O)N)c1cc2Cc3c(-c2cc1)cccc3
298	A7D.mol	O1Cc2c(ccc(OC)c2OC)[C@H]1[C@@H]1N(CCc2c1c(OC)c1OCOc1c2)C(C(=O)N)c1ccc(OC)cc1OC
299	A7U.mol	O1Cc2c(ccc(OC)c2OC)[C@H]1[C@@H]1N(CCc2c1c(OC)c1OCOc1c2)C(C(=O)N)c1ccc(OC)cc1OC
300	A11D.mol	O1Cc2c(ccc(OC)c2OC)[C@H]1[C@@H]1N(CCc2c1c(OC)c1OCOc1c2)C(C(=O)N)c1ccc(OCc2ccccc2)cc1
301	A11U.mol	O1Cc2c(ccc(OC)c2OC)[C@H]1[C@@H]1N(CCc2c1c(OC)c1OCOc1c2)C(C(=O)N)c1ccc(OCc2ccccc2)cc1
302	A16D.mol	O1Cc2c(ccc(OC)c2OC)[C@H]1[C@@H]1N(CCc2c1c(OC)c1OCOc1c2)C(C(=O)N)c1ccc(cc1)C
303	A9.mol	O1Cc2c(ccc(OC)c2OC)[C@H]1[C@@H]1N(CCc2c1c(OC)c1OCOc1c2)C(C(=O)N)c1ccc(cc1)C(C)C
304	A1D.mol	O1Cc2c(ccc(OC)c2OC)[C@H]1[C@@H]1N(CCc2c1c(OC)c1OCOc1c2)C(C(=O)N)c1ccccc1
305	Untitled Document-1	O1Cc2c(ccc(OC)c2OC)[C@H]1[C@@H]1N(CCc2c1c(OC)c1OCOc1c2)C(C(=O)N)c1ccccc1

	mol	SMILES
306	ALU.mol	O1Cc2c(ccc(OC)c2OC)[C@H]1[C@@H]1N(CCc2c1c(OC)c1OCOc1c2)C(C(=O)N)c1ccccc1
307	A8D.mol	O1Cc2c(ccc(OC)c2OC)[C@H]1[C@@H]1N(CCc2c1c(OC)c1OCOc1c2)C(C(=O)N)c1cccnc1
308	A8U.mol	O1Cc2c(ccc(OC)c2OC)[C@H]1[C@@H]1N(CCc2c1c(OC)c1OCOc1c2)C(C(=O)N)c1cccnc1
309	S-14.mol	O1Cc2c(ccc(OC)c2OC)[C@H]1[C@@H]1N(CCc2c1c(OC)c1OCOc1c2)C(C(C)C)C#N
310	S-17.mol	O1Cc2c(ccc(OC)c2OC)[C@H]1[C@@H]1N(CCc2c1c(OC)c1OCOc1c2)C(CC)C#N
311	AM-12.mol	O1Cc2c(ccc(OC)c2OC)[C@H]1[C@@H]1N(CCc2c1c(OC)c1OCOc1c2)C(CN)c1cc2Cc3c(-c2cc1)cccc3
312	AM-16.mol	O1Cc2c(ccc(OC)c2OC)[C@H]1[C@@H]1N(CCc2c1c(OC)c1OCOc1c2)C(CN)c1ccc(cc1)C
313	AM1.mol	O1Cc2c(ccc(OC)c2OC)[C@H]1[C@@H]1N(CCc2c1c(OC)c1OCOc1c2)C(CN)c1ccccc1
314	AM8.mol	O1Cc2c(ccc(OC)c2OC)[C@H]1[C@@H]1N(CCc2c1c(OC)c1OCOc1c2)C(CN)c1cccnc1
315	S47	O1O[C@H]2C=C[C@@]([C@@H]3CC[C@@]4([C@@]1([C@@]23C)C(=O)C=C1[C@@H]2[C@@H](C)[C@@H](CC[C@@]
316	SBEH-40	O1[C@@H](Cc2c(C1=O)c(O)cc(O)c2)Cc1cc(O)cc(C(=O)C)c1O
317	JAOMET4	O1[C@@H](O)[C@@H](O)C(O)[C@H](OC2=C(Oc3c(C2=O)c(O)cc(O)c3)c2ccc(O)cc2O)C1CO
318	QTEH-14	O1[C@@H](O)[C@@H](O)C(O)[C@H](O[C@@H]2CC3=CCC4C5CCC([C@@H](CC[C@H](C(C)C)CC)C)[C@]5(CCC4[
319	SBME-P6	O1[C@@H](O)[C@@H](O)C(O)[C@H](O[C@]2(O)c3c(C(=O)c4c2cccc4O)c(O)cc(c3)CO)C1CO
320	Desmiflavaside B	O1[C@@H](O[C@@H]2C(OC)[C@H](O)[C@@H](OC2C)O[C@H]2CC[C@@]3([C@@H]4C(CC[C@H]3C2)[C@]2(O)[C@
321	Desmiflavaside A	O1[C@@H](O[C@@H]2C(OC)[C@H](O)[C@@H](OC2C)O[C@H]2CC[C@@]3([C@@H]4C(CC[C@H]3C2)[C@]2(O)[C@
322	Desmiflavaside D	O1[C@@H](O[C@@H]2C(O[C@@H](O[C@H]3C(OC)[C@H](O)[C@@H](OC3C)O[C@H]3CC[C@@]4([C@@H]5C(CC[C@
323	Desflavasides A	O1[C@@H](O[C@@H]2C(O[C@@H](O[C@H]3CC[C@@]4([C@@H]5C(CC[C@H]4C3)[C@@]3(O)CC[C@H](C(OC(=O)c
324	Desflavasides B	O1[C@@H](O[C@@H]2C(O[C@@H](O[C@H]3CC[C@@]4([C@@H]5C(CC[C@H]4C3)[C@@]3(O)CC[C@H](C(OC(=O)c
325	Desflavasides C	O1[C@@H](O[C@@H]2C(O[C@@H](O[C@H]3CC[C@@]4([C@@H]5C(CC[C@H]4C3)[C@@]3(O)CC[C@H](C(OC(=O)c
326	S78	O1[C@@H](O[C@@H]2CC[C@]3([C@@H](CC[C@@]4(C3C(=O)C=C3[C@@H]5[C@@H](C)[C@@H](CC[C@@]5(CC[C@
327	S76	O1[C@@H](O[C@@H]2CC[C@]3([C@@H](CC[C@@]4(C3C(=O)C=C3[C@@H]5[C@@H](C)[C@@H](CC[C@@]5(CC[C@
328	S77	O1[C@@H](O[C@@H]2CC[C@]3([C@@H](CC[C@@]4(C3C(=O)C=C3[C@@H]5[C@@H](C)[C@@H](CC[C@@]5(CC[C@
329	S74	O1[C@@H](O[C@@H]2CC[C@]3([C@@H](CC[C@@]4(C3C(=O)C=C3[C@@H]5[C@@H](C)[C@@H](CC[C@@]5(CC[C@
330	S75	O1[C@@H](O[C@@H]2CC[C@]3([C@@H](CC[C@@]4(C3C(=O)C=C3[C@@H]5[C@@H](C)[C@@H](CC[C@@]5(CC[C@
331	S79	O1[C@@H](O[C@@H]2CC[C@]3([C@@H](CC[C@@]4(C3C(=O)C=C3[C@H]5[C@@H](C)[C@@H](CC[C@@]5(CC[C@]
332	BEMK-30	O1[C@@H](O[C@H]2C(O)[C@H](O)[C@@H](OC2CO)OC)[C@@H](O)[C@H](O)C(OC)C1CO
333	Desflavasides D	O1[C@@H](O[C@H]2C(O)[C@H](O)[C@@H](OC2CO)OCC2O[C@@H](O[C@H]3CC[C@@]4([C@@H]5C(CC[C@H]4C3)
334	Desmiflavaside C	O1[C@@H](O[C@H]2CC[C@@]3([C@@H]4C(CC[C@H]3C2)[C@@]2(O)CC[C@H](C(OC(=O)c3ccccc3)C)[C@@]2(C
335	Nizwaside	O1[C@@H](O[C@H]2CC[C@@]3([C@@H]4C(CC[C@H]3C2)[C@@]2(O)CC[C@H](C(OC(=O)c3ccccc3)C)[C@@]2(C
336	NOR.mol	O1[C@@H](c2c(c(OC)c(OC)cc2)C1=O)[C@@H]1NCCc2c1c(OC)c1OCOc1c2
337	QTH-3	O1[C@@H]2[C@@H]3[C@@H](CCC3=C)C(CCC2C(=C)C1=O)=C
338	S39	O1[C@@H]2[C@H]1C[C@]1([C@@H](CC[C@@]3(C1C(=O)C=C1[C@@H]4[C@@H](C)[C@@H](CC[C@@]4(CC[C@]13
339	Incensone	O1[C@@]2(CC[C@]1(C\C=C(\CC\C=C(\CCC2=O)/C)/C)C(C)C)C
340	Incensone.mol	O1[C@@]2(CC[C@]1(C\C=C(\CC\C=C(\CCC2=O)/C)/C)C(C)C)C
341	Epi-Incensole	O1[C@@]2(CC[C@]1(C\C=C(\CC\C=C(\CC[C@H]2O)/C)/C)C(C)C)C
342	Incensole	O1[C@@]2(CC[C@]1(C\C=C(\CC\C=C(\CC[C@H]2O)/C)/C)C(C)C)C
343	Epi-Incensole.mol	O1[C@@]2(CC[C@]1(C\C=C(\CC\C=C(\CC[C@H]2O)/C)/C)C(C)C)C
344	Incensole.mol	O1[C@@]2(CC[C@]1(C\C=C(\CC\C=C(\CC[C@H]2O)/C)/C)C(C)C)C
345	Incensole acetate	O1[C@@]2(CC[C@]1(C\C=C(\CC\C=C(\CC[C@H]2OC(=O)C)/C)/C)C(C)C)C
346	Incensole acetate.mol	O1[C@@]2(CC[C@]1(C\C=C(\CC\C=C(\CC[C@H]2OC(=O)C)/C)/C)C(C)C)C
347	BEMD-14	O1[C@H](O[C@@]2(O[C@H](CO)C(O)[C@H]2O)CO)[C@@H](O)[C@H](O)[C@H](O)C1CO
348	BEMD-14.mol	O1[C@H](O[C@@]2(O[C@H](CO)C(O)[C@H]2O)CO)[C@@H](O)[C@H](O)[C@H](O)C1CO
349	SBME-25	O1[C@H]([C@H](O)[C@@H](O)[C@H](O)[C@H]1CO)[C@H]1c2c(C(=O)c3c1cccc3O)c(O)cc(c2)CO[C@@H]1O[
350	QTH-4	O1[C@H]/2[C@@H](CC\C(=C\CC\C(=C\2)\C)\C)C(=C)C1=O
351	QTH-70	O1[C@H]2[C@@H](CC[C@H](O)[C@@]3([C@@H]2[C@](O)(CC3)C)C)C(=C)C1=O
352	S41	O1[C@H]2[C@@H]1C[C@]1([C@@H](CC[C@@]3(C1C(=O)C=C1[C@@H]4[C@@H](C)[C@@H](CC[C@@]4(CC[C@]13
353	11.cdx	O1[C@](CC[C@@H]1C(OC(=O)C)(C)C)(C(O)COc1ccc(cc1)CCNC(=O)c1ccccc1)C
354	BAEH-30	O1[C@]2([C@H](CC[C@H]1OC)[C@@]1([C@@H](CC2)C(C)(C)C(=O)CC1)C)C
355	JAOETH35	O1c2c(C(=O)C(O)=C1c1cc(O)c(O)cc1)c(O)cc(O)c2
356	JCDACH90	O1c2c(C(=O)C(O)=C1c1cc(OC)c(O)cc1)c(O)cc(OC)c2
357	JAOMET4.mol	O1c2c(C(=O)C(OC(=O)CO)=C1c1ccc(O)cc1O)c(O)cc(O)c2
358	JAOETH-90	O1c2c(C(=O)C(O[C@@H]3O[C@@H](C)[C@H](O)[C@@H](O)[C@H]3O)=C1c1ccc(O)cc1O)c(O)cc(O)c2
359	7C.mol	O1c2c(C3=NC(N=Nc4ccc(OC)cc4)C(C=C3C1NCCc1ccccc1)C#N)cccc2
360	7B.mol	O1c2c(C3=NC(N=Nc4ccccc4)C(C=C3C1NCCc1ccccc1)C#N)cccc2
361	7A.mol	O1c2c(C3=NC(N=Nc4ccccc4)C(C=C3C1NCc1ccccc1)C#N)cccc2
362	6MA.mol	O1c2c(C3=NC4=NN(C(N)C4C=C3C1NCc1ccccc1)c1ccc(cc1)C(O)=O)cccc2
363	6AA.mol	O1c2c(C3=NC4=NN(C(N)C4C=C3C1NCc1ccccc1)c1ccccc1)cccc2
364	QTH-90	O1c2c(C[C@H](O)[C@H]1c1cc(O)c(O)cc1)c(O)cc(O)c2
365	N-1	O1c2c(OC1)cc1c(C(N(CC1)C)C(O)c1ccc(OC)c(OC)c1CO)c2OC
366	N-D-1b	O1c2c(OC1)cc1c(C(N(CC1)CC1CC1)C(O)c1ccc(OC)c(OC)c1CO)c2OC

	mol	SMILES
367	N-D-11b	O1c2c(OC1)cc1c(C(N(CC1)Cc1c3c(cc4c1cccc4)cccc3)C(O)c1ccc(OC)c(OC)c1CO)c2OC
368	N-D-2b	O1c2c(OC1)cc1c(C(N(CC1)Cc1ccccc1)C(O)c1ccc(OC)c(OC)c1CO)c2OC
369	S-2.mol	O1c2c(OC1)cc1c([C@@H](N(CC1)C(C#N)c1cc(Oc3ccccc3)ccc1)[C@@H]1CCc3c1ccc(OC)c3OC)c2OC
370	N-D-18b	O1c2c(OC1)cccc2CN1CCc2c(C1C(O)c1ccc(OC)c(OC)c1CO)c(OC)c1OCOc1c2
371	SBEH-50.mol	O1c2c(c(cc(O)c2)C)C(OC)=CC1=O
372	47-amino-KBA.cdx	O=C1C2C(CCC3C(C(O)=O)(C)C(N)CCC23C)(C)C2(C(C3C(C)C(CCC3(CC2)C)C)=C1)C
373	S97	O=C1C2[C@@]3(C(CC[C@]2(C2C(=C1)C1[C@@H](C)[C@@H](CC[C@@]1(CC2)C)C)C)[C@](C(OCCCCOC(=O)[C@
374	S91	O=C1C2[C@@]3(C(CC[C@]2(C2C(=C1)C1[C@@H](C)[C@@H](CC[C@@]1(CC2)C)C)C)[C@](C(OCCCCOC(=O)[C@
375	S96	O=C1C2[C@@]3(C(CC[C@]2(C2C(=C1)C1[C@@H](C)[C@@H](CC[C@@]1(CC2)C)C)C)[C@](C(OCCCOC(=O)[C@@
376	S90	O=C1C2[C@@]3(C(CC[C@]2(C2C(=C1)C1[C@@H](C)[C@@H](CC[C@@]1(CC2)C)C)C)[C@](C(OCCCOC(=O)[C@@
377	S95	O=C1C2[C@@]3(C(CC[C@]2(C2C(=C1)C1[C@@H](C)[C@@H](CC[C@@]1(CC2)C)C)C)[C@](C(OCCOC(=O)[C@@]
378	S89	O=C1C2[C@@]3(C(CC[C@]2(C2C(=C1)C1[C@@H](C)[C@@H](CC[C@@]1(CC2)C)C)C)[C@](C(OCCOC(=O)[C@@]
379	SF-14	O=C1C2[C@](CCC3[C@@]2(CC[C@H](O)C3(C)C)C)(C)[C@]2(C(=C1)C1[C@@H](C)[C@@H](CC[C@@]1(CC2)C)
380	3-beta-hydroxy-11-oxours-12ene.mol	O=C1C2[C@](CCC3[C@@]2(CC[C@H](O)C3(C)C)C)(C)[C@]2(C(=C1)C1[C@@H](C)[C@@H](CC[C@@]1(CC2)C)
381	PDCM-55-3-hydroxy-20(29)-lupene-11-on	O=C1C2[C@](CCC3[C@@]2(CC[C@H](O)C3(C)C)C)(C)[C@]2(C(C1)C1[C@@](CC2)(CC[C@H]1C(C)=C)C)C
382	3-hydroxy-20(29)-lupene-11-one.mol	O=C1C2[C@](CCC3[C@@]2(CC[C@H](O)C3(C)C)C)(C)[C@]2(C(C1)C1[C@@](CC2)(CC[C@H]1C(C)=C)C)C
383	S20	O=C1C2[C@](CCC3[C@@]2(Cc2nc4c(nc2[C@H]3C)cccc4)C)(C)[C@@]2(CC[C@@]3([C@H](C2=C1)[C@@H](C)
384	S21	O=C1C2[C@](CCC3[C@@]2(Cc2nccnc2[C@H]3C)C)(C)[C@@]2(CC[C@@]3([C@H](C2=C1)[C@@H](C)[C@@H](C
385	3-epi-a-KBA.mol	O=C1C2[C@](CCC3[C@](C(O)=O)(C)[C@@H](O)CC[C@]23C)(C)[C@@]2(CC[C@@]3([C@@H](CC(CC3)(C)C)C2
386	3-epi-b-KBA	O=C1C2[C@](CCC3[C@](C(O)=O)(C)[C@@H](O)CC[C@]23C)(C)[C@@]2(CC[C@@]3([C@H](C2=C1)[C@@H](C)
387	3-epi-a-AKBA	O=C1C2[C@](CCC3[C@](C(O)=O)(C)[C@@H](OC(=O)C)CC[C@]23C)(C)[C@@]2(CC[C@@]3([C@@H](CC(CC3)(
388	3-epi-b-AKBA	O=C1C2[C@](CCC3[C@](C(O)=O)(C)[C@@H](OC(=O)C)CC[C@]23C)(C)[C@@]2(CC[C@@]3([C@H](C2=C1)[C@
389	a-KBA	O=C1C2[C@](CCC3[C@](C(O)=O)(C)[C@H](O)CC[C@]23C)(C)[C@@]2(CC[C@@]3([C@@H](CC(CC3)(C)C)C2=
390	Keto-b-Boswellic acid-bKBA	O=C1C2[C@](CCC3[C@](C(O)=O)(C)[C@H](O)CC[C@]23C)(C)[C@]2(C(=C1)C1[C@@H](C)[C@@H](CC[C@@]1
391	Keto-boswellic acid.mol	O=C1C2[C@](CCC3[C@](C(O)=O)(C)[C@H](O)CC[C@]23C)(C)[C@]2(C(=C1)C1[C@@H](C)[C@@H](CC[C@@]1
392	a-AKBA	O=C1C2[C@](CCC3[C@](C(O)=O)(C)[C@H](OC(=O)C)CC[C@]23C)(C)[C@@]2(CC[C@@]3([C@@H](CC(CC3)(C
393	3-a-Acetyl-11-keto-b-Boswellic acid-A	O=C1C2[C@](CCC3[C@](C(O)=O)(C)[C@H](OC(=O)C)CC[C@]23C)(C)[C@]2(C(=C1)C1[C@@H](C)[C@@H](CC
394	BCL-21.mol	O=C1C2[C@](CCC3[C@](C(O)=O)(C)[C@H](OC(=O)C)CC[C@]23C)(C)[C@]2(C(=C1)C1[C@@H](C)[C@@H](CC
395	S85	O=C1C2[C@](CCC3[C@](C(OCCCCOC(=O)[C@@]4(C5CC[C@@]6(C([C@]5(CC[C@H]4O)C)C(=O)C=C4C5[C@@H](
396	S94	O=C1C2[C@](CCC3[C@](C(OCCCCOC(=O)[C@@]4(C5CC[C@@]6(C([C@]5(CC[C@H]4OC(=O)C)C)C(=O)C=C4C5[
397	S82	O=C1C2[C@](CCC3[C@](C(OCCCCOC(=O)[C@@]4(C5CC[C@@]6(C([C@]5(CC[C@H]4OC(=O)C)C)C(=O)C=C4C5[
398	S84	O=C1C2[C@](CCC3[C@](C(OCCCOC(=O)[C@@]4(C5CC[C@@]6(C([C@]5(CC[C@H]4O)C)C(=O)C=C4C5[C@@H](C
399	S93	O=C1C2[C@](CCC3[C@](C(OCCCOC(=O)[C@@]4(C5CC[C@@]6(C([C@]5(CC[C@H]4OC(=O)C)C)C(=O)C=C4C5[C
400	S81	O=C1C2[C@](CCC3[C@](C(OCCCOC(=O)[C@@]4(C5CC[C@@]6(C([C@]5(CC[C@H]4OC(=O)C)C)C(=O)C=C4C5[C
401	S83	O=C1C2[C@](CCC3[C@](C(OCCOC(=O)[C@@]4(C5CC[C@@]6(C([C@]5(CC[C@H]4O)C)C(=O)C=C4C5[C@@H](C)
402	S92	O=C1C2[C@](CCC3[C@](C(OCCOC(=O)[C@@]4(C5CC[C@@]6(C([C@]5(CC[C@H]4OC(=O)C)C)C(=O)C=C4C5[C@
403	S80	O=C1C2[C@](CCC3[C@](C(OCCOC(=O)[C@@]4(C5CC[C@@]6(C([C@]5(CC[C@H]4OC(=O)C)C)C(=O)C=C4C5[C@
404	S4	O=C1C2[C@](CCC3[C@](C(OCc4ccccc4)=O)(C)[C@H](O)CC[C@]23C)(C)[C@@]2(CC[C@@]3([C@H](C2=C1)[
405	S19	O=C1C2[C@](CCC3[C@](c4nc5c(nc4C[C@]23C)cccc5)(C(OCc2ccccc2)=O)C)(C)[C@@]2(CC[C@@]3([C@H](
406	S22	O=C1C2[C@](CCC3[C@](c4nccnc4C[C@]23C)(C(OCc2ccccc2)=O)C)(C)[C@@]2(CC[C@@]3([C@H](C2=C1)[C
407	S58	O=C1C2[C@](CC[C@@H]([C@](CN)(C(OCc3ccccc3)=O)C)C2(C)C)(C)[C@@]2(CC[C@@]3([C@H](C2=C1)[C@@
408	S9	O=C1C2[C@](CC[C@@H]3[C@@]2(Cc2c([nH]c4c2cccc4)C3(C)C)C)(C)[C@@]2(CC[C@@]3([C@H](C2=C1)[C@
409	S49	O=C1C2[C@](CC[C@@H]3[C@]2(C)[C@@H](OC)C=C[C@@]3(C(OC)=O)C)(C)[C@@]2(CC[C@@]3([C@H](C2=C1)
410	S48	O=C1C2[C@](CC[C@@H]3[C@]2(C)[C@H](OC)C=C[C@@]3(C(OC)=O)C)(C)[C@@]2(CC[C@@]3([C@H](C2=C1)[
411	S70	O=C1C2[C@](CC[C@H]3[C@](C(=O)NCC(OCC)=O)(C)[C@H](OC(=O)C)CC[C@]23C)(C)[C@@]2(CC[C@@]3([C@
412	S71	O=C1C2[C@](CC[C@H]3[C@](C(=O)NCCC(OC)=O)(C)[C@H](OC(=O)C)CC[C@]23C)(C)[C@@]2(CC[C@@]3([C@
413	S73	O=C1C2[C@](CC[C@H]3[C@](C(=O)NCCCO)(C)[C@H](OC(=O)C)CC[C@]23C)(C)[C@@]2(CC[C@@]3([C@H](C2
414	S72	O=C1C2[C@](CC[C@H]3[C@](C(=O)N[C@H](CCC(OC)=O)C(OC)=O)(C)[C@H](OC(=O)C)CC[C@]23C)(C)[C@@]
415	S59	O=C1C2[C@](CC[C@H]3[C@](C(O)=O)(C)[C@@H](N)CC[C@]23C)(C)[C@@]2(CC[C@@]3([C@H](C2=C1)[C@@H
416	S27	O=C1C2[C@](CC[C@H]3[C@](C(OC)=O)(C)[C@@H](O)CC[C@]23C)(C)[C@@]2(CC[C@@]3([C@H](C2=C1)[C@@
417	S32	O=C1C2[C@](CC[C@H]3[C@](C(OC)=O)(C)[C@@H](O)CC[C@]23C)(C)[C@@]2(CC[C@@]3([C@H](C2=C1)[C@@
418	S66	O=C1C2[C@](CC[C@H]3[C@](C(OC)=O)(C)[C@H](CC[C@]23C)C(O)=O)(C)[C@@]2(CC[C@@]3([C@H](C2=C1)
419	S65	O=C1C2[C@](CC[C@H]3[C@](C(OC)=O)(C)[C@H](CC[C@]23C)C=O)(C)[C@@]2(CC[C@@]3([C@H](C2=C1)[C@
420	S35	O=C1C2[C@](CC[C@H]3[C@](C(OC)=O)(C)[C@H](O)C=C[C@]23C)(C)[C@@]2(CC[C@@]3([C@H](C2=C1)[C@@
421	S24	O=C1C2[C@](CC[C@H]3[C@](C(OC)=O)(C)[C@H](O)CC[C@]23C)(C)[C@@]2(CC[C@@]3([C@H](C2=C1)[C@@H
422	S29	O=C1C2[C@](CC[C@H]3[C@](C(OC)=O)(C)[C@H](O)CC[C@]23C)(C)[C@@]2(CC[C@@]3([C@H](C2=C1)[C@@H
423	S50	O=C1C2[C@](CC[C@H]3[C@](C(OC)=O)(C)[C@H](O)CC[C@]23C)(C)[C@@]2(CC[C@@]3([C@H](C2=C1)[C@@H
424	S38	O=C1C2[C@](CC[C@H]3[C@](C(OC)=O)(C)[C@H](O)[C@@H](O)C[C@]23C)(C)[C@@]2(CC[C@@]3([C@H](C2=
425	S10	O=C1C2[C@](CC[C@H]3[C@](C(OCc4ccccc4)=O)(C)/C(=N/Nc4ccc([N+](=O)[O-])cc4[N+](=O)[O-])/CC[
426	S57	O=C1C2[C@](CC[C@H]3[C@](C(OCc4ccccc4)=O)(C)/C(=N/O)/CC[C@]23C)(C)[C@@]2(CC[C@@]3([C@H](C2
427	S34	O=C1C2[C@](CC[C@H]3[C@](C=CC[C@]23C)(C(OC)=O)C)(C)[C@@]2(CC[C@@]3([C@H](C2=C1)[C@@H](C)[C

	mol	SMILES
428	S44	O=C1C2[C@](CC[C@H]3[C@](C=CC[C@]23C)(C(OC)=O)C)(C)[C@@]2(CC[C@@]3([C@H](C2=C1)[C@H](CCC3)
429	S36	O=C1C2[C@](CC[C@H]3[C@](CCC[C@]23C)(C(OC)=O)C)(C)[C@@]2(CC[C@@]3([C@H](C2=C1)[C@@H](C)[C@
430	S7	O=C1C2[C@](CC[C@H]3[C@](CCC[C@]23C)(C(OCc2ccccc2)=O)C)(C)[C@@]2(CC[C@@]3([C@H](C2=C1)[C@@
431	S5	O=C1C2[C@](CC[C@H]3[C@](c4[nH]c5c(c4C[C@]23C)cccc5)(C(OCc2ccccc2)=O)C)(C)[C@@]2(CC[C@@]3(
432	S17	O=C1C=C[C@@]2(C3[C@](CCC2[C@@H]1C)(C)[C@@]1(CC[C@@]2([C@H](C1=CC3=O)[C@@H](C)[C@@H](CC2)C
433	S25	O=C1C=C[C@]2([C@@H](CC[C@@]3(C2C(=O)C=C2[C@@H]4[C@@H](C)[C@@H](CC[C@@]4(CC[C@]23C)C)C)C)[
434	S30	O=C1C=C[C@]2([C@@H](CC[C@@]3(C2C(=O)C=C2[C@@H]4[C@@H](C)[C@@H](CC[C@@]4(CC[C@]23C)C)C)C)[
435	DJ-501	O=C1CC2CC=C3C4CC[C@H]([C@@H](CCCC(C)C)C)[C@]4(CCC3[C@]2(CC1)C)C
436	S16	O=C1CC[C@@]2(C3[C@](CCC2=C1C)(C)[C@@]1(CC[C@@]2([C@H](C1=CC3=O)[C@@H](C)[C@@H](CC2)C)C)C)
437	SF-1P-2	O=C1CC[C@@]2(C3[C@](CCC2C1(C)C)(C)[C@]1(C(=CC3=O)C2[C@@H](C)[C@@H](CC[C@@]2(CC1)C)C)C)C
438	S2	O=C1CC[C@@]2(C3[C@](CCC2[C@@H]1C)(C)[C@@]1(CC[C@@]2([C@H](C1=CC3=O)[C@@H](C)[C@@H](CC2)C)
439	S3	O=C1CC[C@@]2(C3[C@](CCC2[C@]1(C(O)=O)C)(C)[C@@]1(CC[C@@]2([C@H](C1=CC3=O)[C@@H](C)[C@@H](
440	DJEH-10-S1	O=C1CC[C@]2(C(CC=C3C4CC[C@H]([C@@H](CCCC(C)C)C)[C@]4(CCC23)C)[C@@H]1C)C
441	11b-Hydroxy-3-oxours-12-ene	O=C1CC[C@]2(C(CC[C@@]3(C2[C@@H](O)C=C2C4[C@@H](C)[C@@H](CC[C@@]4(CC[C@]23C)C)C)C)C1(C)C)C
442	11ß_Hydroxy_3_oxours_12_ene.mol	O=C1CC[C@]2(C(CC[C@@]3(C2[C@@H](O)C=C2C4[C@@H](C)[C@@H](CC[C@@]4(CC[C@]23C)C)C)C)C1(C)C)C
443	11-beta-hydroxy-3-oxours-12ene.mol	O=C1CC[C@]2(C(CC[C@@]3(C2[C@@H](O)C=C2C4[C@@H](C)[C@@H](CC[C@@]4(CC[C@]23C)C)C)C)C1(C)C)C
444	BAEH-10	O=C1CC[C@]2(C(CC[C@](O)(C)C2CC\C=C(\CC\C=C(\CC\C=C(\C(O)=O)/C)/C)/C)C1(C)C)C
445	Nizwanone	O=C1CC[C@]2(C(C[C@H](O)[C@@]3(C2CC=C2C4[C@@H](C)[C@@H](CC[C@@]4(CC[C@]23C)C)C)C)C1(C)C)C
446	Nizwanon.mol	O=C1CC[C@]2(C(C[C@H](O)[C@@]3(C2CCC2C4[C@@H](C)[C@@H](CC[C@@]4(CC[C@]23C)C)C)C)C1(C)C)C
447	S54	O=C1CC[C@]2([C@@H](CC[C@@]3(C2C(=O)C=C2[C@@H]4[C@@H](C)[C@@H](CC[C@@]4(CC[C@]23C)C)C)C)[C
448	S26	O=C1CC[C@]2([C@@H](CC[C@@]3(C2C(=O)C=C2[C@@H]4[C@@H](C)[C@@H](CC[C@@]4(CC[C@]23C)C)C)C)[C
449	S31	O=C1CC[C@]2([C@@H](CC[C@@]3(C2C(=O)C=C2[C@@H]4[C@@H](C)[C@@H](CC[C@@]4(CC[C@]23C)C)C)C)[C
450	S55	O=C1CC[C@]2([C@@H](CC[C@@]3(C2C(=O)C=C2[C@@H]4[C@@H](C)[C@@H](CC[C@@]4(CC[C@]23C)C)C)C)[C
451	S8	O=C1CC[C@]2([C@@H](CC[C@@]3(C2C(=O)C=C2[C@@H]4[C@@H](C)[C@@H](CC[C@@]4(CC[C@]23C)C)C)C)[C
452	S15	O=C1CC[C@]2([C@@H](CC[C@@]3(C2C(=O)C=C2[C@@H]4[C@@H](C)[C@@H](CC[C@@]4(CC[C@]23C)C)C)C)[C
453	S56	O=C1CC[C@]2([C@@H](CC[C@@]3(C2C(=O)C=C2[C@@H]4[C@@H](C)[C@@H](CC[C@@]4(CC[C@]23C)C)C)C)[C
454	S1	O=C1CC[C@]2([C@@H](CC[C@@]3(C2CC=C2[C@@H]4[C@@H](C)[C@@H](CC[C@@]4(CC[C@]23C)C)C)C)C1(C)C
455	6G.mol	O=C1N(CCc2ccccc2)C(c2cc3c(nc2-n2nnc(c12)-c1ccccc1)cccc3)C(=O)NC(C)(C)C
456	6H.mol	O=C1N(CCc2ccccc2)C(c2cc3c(nc2-n2nnc(c12)-c1ccccc1)cccc3)C(=O)NC1CCCCC1
457	6I.mol	O=C1N(CCc2ccccc2)C(c2cc3c(nc2-n2nncc12)cccc3)C(=O)NC(C)(C)C
458	6J.mol	O=C1N(CCc2ccccc2)C(c2cc3c(nc2-n2nncc12)cccc3)C(=O)NC1CCCCC1
459	6C.mol	O=C1N(CCc2ccccc2)C(c2cc3cc(ccc3nc2-n2nnc(c12)-c1ccccc1)C)C(=O)NC(C)(C)C
460	6N.mol	O=C1N(Cc2ccc(cc2)C)C(c2cc3c(nc2-n2nncc12)cccc3)C(=O)NC(C)(C)C
461	6M.mol	O=C1N(Cc2ccc(cc2)C)C(c2cc3c(nc2-n2nncc12)cccc3)C(=O)NC1CCCCC1
462	6O.mol	O=C1N(Cc2ccc(cc2)C)C(c2cc3cc(ccc3nc2-n2nnc(c12)-c1ccccc1)C)C(=O)NC(C)(C)C
463	6K.mol	O=C1N(Cc2ccc(cc2)C)C(c2cc3cc(ccc3nc2-n2nnc(c12)-c1ccccc1)C)C(=O)NC1CCCCC1
464	6B.mol	O=C1N(Cc2ccccc2)C(c2cc3c(nc2-n2nnc(c12)-c1ccccc1)cccc3)C(=O)NC(C)(C)C
465	6A.mol	O=C1N(Cc2ccccc2)C(c2cc3c(nc2-n2nnc(c12)-c1ccccc1)cccc3)C(=O)NC1CCCCC1
466	6F.mol	O=C1N(Cc2ccccc2)C(c2cc3c(nc2-n2nncc12)cccc3)C(=O)NC(C)(C)C
467	6E.mol	O=C1N(Cc2ccccc2)C(c2cc3c(nc2-n2nncc12)cccc3)C(=O)NC1CCCCC1
468	6L.mol	O=C1N(Cc2ccccc2)C(c2cc3cc(ccc3nc2-n2nnc(c12)-c1ccccc1)C)C(=O)NC(C)(C)C
469	6D.mol	O=C1N(Cc2ccccc2)C(c2cc3cc(ccc3nc2-n2nnc(c12)-c1ccccc1)C)C(=O)NC1CCCCC1
470	R-NPG-21.cdx	O=C1N(\C(=C/c2ccc(cc2)\C=C(\N2C(=O)c3c(cccc3)C2=O)/C(O)=O)\C(O)=O)C(=O)c2c1cccc2
471	R-NPG-16.cdx	O=C1N(\C(=C\c2cc(ccc2)\C=C(\N2C(=O)c3c(cccc3)C2=O)/C(O)=O)\C(O)=O)C(=O)c2c1cccc2
472	R-NPG-17.cdx	O=C1N(\C(=C\c2ccc([N+](=O)[O-])cc2)\C(O)=O)C(=O)c2c1cccc2
473	R-NPG-18.cdx	O=C1N(\C(=C\c2ccc(cc2)C(C)C)\C(O)=O)C(=O)c2c1cccc2
474	R-NPG-5.cdx	O=C1N(\C(=C\c2ccc(cc2)C(OC)=O)\C(O)=O)C(=O)c2c1cccc2
475	R-NPG-12.cdx	O=C1N(\C(=C\c2ccc(cc2)C)\C(O)=O)C(=O)c2c1cccc2
476	R-NPG-11.cdx	O=C1N(\C(=C\c2ccc(cc2C)C)\C(O)=O)C(=O)c2c1cccc2
477	R-NPG-1.cdx	O=C1N(\C(=C\c2ccccc2)\C(O)=O)C(=O)c2c1cccc2
478	R-NPG-23.cdx	O=C1N(\C(=C\c2ncccc2)\C(O)=O)C(=O)c2c1cccc2
479	S18	O=C1NCC[C@@]2(C3[C@](CCC2[C@]1(C(=O)N)C)(C)[C@@]1(CC[C@@]2([C@H](C1=CC3=O)[C@@H](C)[C@@H]
480	S6	O=C1NN=C2CC[C@]3([C@@H](CC[C@@]4(C3C(=O)C=C3[C@@H]5[C@@H](C)[C@@H](CC[C@@]5(CC[C@]34C)C)C
481	S62	O=C1[C@@]([C@@H]2CC[C@@]3(C([C@]2(C=C1NC(=O)C)C)C(=O)C=C1[C@@H]2[C@@H](C)[C@@H](CC[C@@]2(
482	S68	O=C1[C@@]([C@@H]2CC[C@@]3(C([C@]2(C[C@@H]1O)C)C(=O)C=C1[C@@H]2[C@@H](C)[C@@H](CC[C@@]2(CC
483	SF-10-3	OC(=O)CCCCCCCCCCCCCCCCCCCCCCCCCC
484	1.cdx	OC(=O)\C=C\c1ccc(cc1)\C=C\C(O)=O
485	25-Isoserratol	OC(C)(C)C1CC=C(CCCC(=CCCC(=CC1)C)C)C
486	13-Duva-4-8-13-triene-1-3-?-diol	OC1(CC(O)C=C(CCC=C(CCC(C=C1)C(C)C)C)C)C
487	1-Serratol	OC1(CC=C(CCCC(=CCCC(=CC1)C)C)C)C(C)C
488	15-Duva-3,9,13-triene-1,5-?-diol-1-ac	OC1(CC=CC(O)(CCC=C(CCC(C=C1)C(C)C)C)C)C

	mol	SMILES
489	12-Thumbergol	OC1(CCC=C(CCC=C(CCC(C=C1)C(C)=C)C)C)C
490	S63	OC1(C[C@]2([C@H](CC[C@@]3(C2C(=O)C=C2[C@@H]4[C@@H](C)[C@@H](CC[C@@]4(CC[C@]23C)C)C)C)[C@]
491	2.cdx	OC1(C\C=C(\C)/C)C(=O)c2c(NC1=O)cccc2
492	S69	OC1=C[C@]2([C@@H](CC[C@@]3(C2C(=O)C=C2[C@@H]4[C@@H](C)[C@@H](CC[C@@]4(CC[C@]23C)C)C)C)[C@
493	35-2a,3a-dihydroxy-urs-12-ene-24-oic	OC1C(C2CCC3(C(CC=C4C5C(C)C(CCC5(CCC34C)C)C)C2(CC1O)C)C)(C(O)=O)C
494	PDCM-28	OC1C2[C@](CCC3[C@@]2(CCC(=O)C3(C)C)C)(C)[C@]2(C(C1)C1[C@@](CC2)(CC[C@H]1C(C)=C)C)C
495	11-hydroxy 20(29)-lupene-3-one.mol	OC1C2[C@](CCC3[C@@]2(CCC(=O)C3(C)C)C)(C)[C@]2(C(C1)C1[C@@](CC2)(CC[C@H]1C(C)=C)C)C
496	DJEH-2	OC1CC2CC=C3C4CC[C@H]([C@@H](CCC(C(C)C)CC)C)[C@]4(CCC3[C@]2(CC1)C)C
497	DJEH-11	OC1CC2CC=C3C4CC[C@H]([C@@H](CCCC(C)C)C)[C@]4(CCC3[C@]2(CC1)C)C
498	38-9,11-dehydro-b-boswellic acid	OC1CCC2(C(CCC3(C2=CC=C2C4C(C)C(CCC4(CCC23C)C)C)C)C1(C(O)=O)C)C
499	40-9,11-dehydro-?- boswellic acid	OC1CCC2(C(CCC3(C2=CC=C2C4CC(CCC4(CCC23C)C)(C)C)C)C1(C(O)=O)C)C
500	NJAODCH-75C	OC1CCC2(C(CCC3(C2CC=C2C4C(C)C(CCC4(CCC23C)C(O)=O)C)C)C1(C)C)C
501	28-b-Boswellic acid	OC1CCC2(C(CCC3(C2CC=C2C4C(C)C(CCC4(CCC23C)C)C)C)C1(C(O)=O)C)C
502	44-3-epi-b-Boswellic acid	OC1CCC2(C(CCC3(C2CC=C2C4C(C)C(CCC4(CCC23C)C)C)C)C1(C(O)=O)C)C
503	BEDH-40	OC1CCC2(C(CCC3(C2CC=C2C4C(C)C(CCC4(CCC23C)C)C)C)C1(C)C)C
504	a-amyrin	OC1CCC2(C(CCC3(C2CC=C2C4C(C)C(CCC4(CCC23C)C)C)C)C1(C)C)C
505	32-?-Boswellic acid	OC1CCC2(C(CCC3(C2CC=C2C4CC(CCC4(CCC23C)C)(C)C)C)C1(C(O)=O)C)C
506	46-3-epi-?-Boswellic acid	OC1CCC2(C(CCC3(C2CC=C2C4CC(CCC4(CCC23C)C)(C)C)C)C1(C(O)=O)C)C
507	QTH-25	OC1CCC2(C(CCC3(C2CCC2C4C(CCC23C)(CCC4C(C)=C)C(O)=O)C)C1(C)C)C
508	29-11-keto-b-Boswellic acid	OC1CCC2(C3C(CCC2C1(C(O)=O)C)(C)C1(C(C2C(C)C(CCC2(CC1)C)C)=CC3=O)C)C
509	45-3-epi-11-keto-b-Boswellic acid	OC1CCC2(C3C(CCC2C1(C(O)=O)C)(C)C1(C(C2C(C)C(CCC2(CC1)C)C)=CC3=O)C)C
510	34-3,11-dihydroxy-12-ene-24-oic acids	OC1CCC2(C3C(CCC2C1(C(O)=O)C)(C)C1(C(C2C(C)C(CCC2(CC1)C)C)=CC3O)C)C
511	DJEH-10	OC1CC[C@]2(C(CC=C3C4CC[C@H]([C@@H](CCCC(C)C)C)[C@]4(CCC23)C)[C@@H]1C)C
512	NBEH-101	OC1CC[C@]2(C(CC[C@@]3(C2CCC2C4[C@](CCC4C(C)=C)(CC[C@]23C)C)C)C1(C)C)C
513	Lupeol.mol	OC1CC[C@]2(C(CC[C@@]3(C2CCC2C4[C@](CCC4C(C)=C)(CC[C@]23C)C)C)C1(C)C)C
514	BAEH-5	OC1CC[C@]2(C(CC[C@](O)(C)C2CC\C=C(\CC\C=C(\CC\C=C(\C(O)=O)/C)/C)/C)C1(C)C)C
515	6.cdx	OC=1c2c(NC(=O)C=1C\C=C(\C)/C)cccc2
516	R-NPG-6.cdx	OC=1c2c(cccc2)C(CC=1)\C=C(\N1C(=O)c2c(cccc2)C1=O)/C(O)=O
517	NJAODH-70	OCCCCCCCCCCCCCCCCCCCCC
518	BEMD-40	O[C@@H]1CC2=CCC3C4CCC([C@@H](CC[C@H](C(C)C)CC)C)[C@]4(CCC3[C@]2(CC1)C)C
519	UA.mol	O[C@@H]1CC2C[C@H](O)[C@H]3[C@@H]4CC[C@H]([C@@H](CCC(=O)N)C)[C@]4(CC[C@@H]3[C@]2(CC1)C)C
520	9,11-dehydro-b-Boswellic acid	O[C@@H]1CC[C@]2(C(CC[C@@]3(C2=CC=C2C4[C@@H](C)[C@@H](CC[C@@]4(CC[C@]23C)C)C)C)[C@]1(C(O)=
521	3-beta,24-dihydroxius-9(11)-12 diene.	O[C@@H]1CC[C@]2(C(CC[C@@]3(C2=CC=C2C4[C@@H](C)[C@@H](CC[C@@]4(CC[C@]23C)C)C)C)[C@]1(CO)C)
522	Olean-11-13 (18).mol	O[C@@H]1CC[C@]2(C(CC[C@@]3(C2C=CC2=C4[C@@H](C)[C@@H](CC[C@@]4(CC[C@]23C)C)C)C)[C@]1(CO)C)
523	Ursolic Acid.mol	O[C@@H]1CC[C@]2(C(CC[C@@]3(C2C=CC2[C@@H]4[C@@H](C)[C@@H](CC[C@@]4(CC[C@]23C)C(O)=O)C)C)C1
524	Alfa-Amyrin.mol	O[C@@H]1CC[C@]2(C(CC[C@@]3(C2C=CC2[C@@H]4[C@@H](C)[C@@H](CC[C@@]4(CC[C@]23C)C)C)C)C1(C)C)
525	Epi-amyrine	O[C@@H]1CC[C@]2(C(CC[C@@]3(C2CC=C2C4[C@@H](C)[C@@H](CC[C@@]4(CC[C@]23C)C)C)C)C1(C)C)C
526	PDCM-701	O[C@@H]1CC[C@]2(C(CC[C@@]3(C2CC=C2C4[C@@H](C)[C@@H](CC[C@@]4(CC[C@]23C)C)C)C)[C@]1(C(O)=O
527	B-Boswellic acid	O[C@@H]1CC[C@]2(C(CC[C@@]3(C2CC=C2C4[C@@H](C)[C@@H](CC[C@@]4(CC[C@]23C)C)C)C)[C@]1(C(O)=O
528	3-epi-b-Amyrin	O[C@@H]1CC[C@]2(C(CC[C@@]3(C2CC=C2[C@@H]4CC(CC[C@@]4(CC[C@]23C)C)(C)C)C)C1(C)C)C
529	a-Boswellic Acid	O[C@@H]1CC[C@]2(C(CC[C@@]3(C2CC=C2[C@@H]4CC(CC[C@@]4(CC[C@]23C)C)(C)C)C)[C@]1(C(O)=O)C)C
530	S88	O[C@@H]1CC[C@]2(C(CC[C@@]3(C2CC=C2[C@@H]4[C@@H](C)[C@@H](CC[C@@]4(CC[C@]23C)C)C)C)[C@]1(C
531	S87	O[C@@H]1CC[C@]2(C(CC[C@@]3(C2CC=C2[C@@H]4[C@@H](C)[C@@H](CC[C@@]4(CC[C@]23C)C)C)C)[C@]1(C
532	S86	O[C@@H]1CC[C@]2(C(CC[C@@]3(C2CC=C2[C@@H]4[C@@H](C)[C@@H](CC[C@@]4(CC[C@]23C)C)C)C)[C@]1(C
533	Lupeol.mol	O[C@@H]1CC[C@]2(C(CC[C@@]3(C2CCC2C4C[C@@H](CC[C@@]4(CC[C@]23C)C)C(O)=O)C)C1(C)C)C
534	L-A.mol	O[C@@H]1CC[C@]2([C@@H](C1)CC[C@H]1[C@@H]3CCC([C@H](CCC(=O)N)C)[C@]3(CC[C@@H]12)C)C.CC
535	S53	O[C@@H]1CC[C@]2([C@@H](CC[C@@]3(C2=CC=C2[C@@H]4[C@@H](C)[C@@H](CC[C@@]4(CC[C@]23C)C)C)[
536	S28	O[C@@H]1CC[C@]2([C@@H](CC[C@@]3(C2C(C=C2[C@@H]4[C@@H](C)[C@@H](CC[C@@]4(CC[C@]23C)C)C)=C)
537	S33	O[C@@H]1CC[C@]2([C@@H](CC[C@@]3(C2C(C=C2[C@@H]4[C@@H](C)[C@@H](CC[C@@]4(CC[C@]23C)C)C)=C)
538	S51	O[C@@H]1CC[C@]2([C@@H](CC[C@@]3(C2[C@@H](O)C=C2[C@@H]4[C@@H](C)[C@@H](CC[C@@]4(CC[C@]23C)
539	S52	O[C@@H]1CC[C@]2([C@@H](CC[C@@]3(C2[C@H](O)C=C2[C@@H]4[C@@H](C)[C@@H](CC[C@@]4(CC[C@]23C)C
540	FR-40	O[C@@H]1[C@](C2CC[C@@]3(C(CC=C4C5[C@@](CC[C@@]34C)(CC[C@H]5C(C)=C)C)[C@]2(C[C@@H]1O)C)C)(
541	SF-7-2	O[C@H]1CC[C@]2(C(CC[C@@]3(C2=CC=C2C4[C@@H](C)[C@@H](CC[C@@]4(CC[C@]23C)C)C)C)C1(C)C)C
542	3b, 24-dihydroxiurs, 9(11):12 diene	O[C@H]1CC[C@]2(C(CC[C@@]3(C2=CC=C2C4[C@@H](C)[C@@H](CC[C@@]4(CC[C@]23C)C)C)C)[C@]1(CO)C)C
543	3, 11-dihydroxy-12-enen-24 oic acid	O[C@H]1CC[C@]2(C(CC[C@@]3(C2C=CC2=C4CC(CC[C@@]4(CC[C@]23C)C)(C)C)C)[C@]1(CO)C)C
544	Olean-11,13(18)-dien-3b, 24 diol	O[C@H]1CC[C@]2(C(CC[C@@]3(C2C=CC2=C4CC(CC[C@@]4(CC[C@]23C)C)(C)C)C)[C@]1(CO)C)C
545	ALPHA-amyrine acetate	O[C@H]1CC[C@]2(C(CC[C@@]3(C2CC=C2C4[C@@H](C)[C@@H](CC[C@@]4(CC[C@]23C)C)C)C)C1(C)C)C
546	R12	O[C@H]1CC[C@]2(C(CC[C@@]3(C2CC=C2C4[C@@H](C)[C@@H](CC[C@@]4(CC[C@]23C)C)C)C)C1(C)C)C
547	R12.mol	O[C@H]1CC[C@]2(C(CC[C@@]3(C2CC=C2C4[C@@H](C)[C@@H](CC[C@@]4(CC[C@]23C)C)C)C)C1(C)C)C
548	3-epi-a-Boswellic Acid	O[C@H]1CC[C@]2(C(CC[C@@]3(C2CC=C2[C@@H]4CC(CC[C@@]4(CC[C@]23C)C)(C)C)C)[C@]1(C(O)=O)C)C
549	3-epi-b-Boswellic acid	O[C@H]1CC[C@]2(C(CC[C@@]3(C2CC=C2[C@@H]4[C@@H](C)[C@@H](CC[C@@]4(CC[C@]23C)C)C)C)[C@]1(C(

	mol	SMILES
550	HJCDEH-15	O[C@H]1CC[C@]2(C(CC[C@@]3(C2CCC2C4[C@@](CC[C@@]23C)(CC[C@H]4C(C)=C)C(O)=O)C)C1(C)C)C
551	NJAOMD2	O[C@H]1CC[C@]2(C(CC[C@@]3(C2CCC2C4[C@@](CC[C@@]23C)(CC[C@H]4C(C)=C)C(O)=O)C)C1(C)C)C
552	PDCM-55	O[C@H]1CC[C@]2(C(CC[C@@]3(C2CCC2C4[C@@](CC[C@]23C)(CC[C@H]4C(C)=C)C)C)C1(C)C)C
553	PDCM-64	O[C@H]1CC[C@]2(C(CC[C@@]3(C2CCC2C4[C@@](CC[C@]23C)(CC[C@H]4C(C)=C)C)C)[C@]1(C(O)=O)C)C
554	CH-A.mol	O[C@H]1C[C@H]2[C@H]([C@@H]3CC[C@H](C(CCC(=O)N)C)[C@@]13C)CC[C@@H]1C[C@H](O)CC[C@@]12C
555	DA.mol	O[C@H]1C[C@H]2[C@H]([C@@H]3CC[C@H](C(CCC(=O)N)C)[C@@]13C)CC[C@@H]1C[C@H](O)CC[C@@]12C
556	C-A.mol	O[C@H]1C[C@H]2[C@H]([C@@H]3CC[C@H](C(CCC(=O)N)C)[C@@]13C)[C@H](O)C[C@@H]1C[C@H](O)CC[C@@]
557	Commic acid-D.mol	O[C@H]1[C@@](C2CC[C@@]3(C(CC=C4[C@@H]5[C@@H](C)[C@@H](CC[C@@]5(CC[C@@]34C)C)C)[C@]2(C[C@@
558	QTH-301	Oc1c2c(cc(c1)C)C(=O)c1c(C2=O)c(O)cc(O)c1
559	QTH-20	Oc1c2c(cc(c1)CO)C(=O)c1c(C2=O)c(O)cc(O)c1
560	SBEH-20	Oc1c2c(cc(c1)CO)C(=O)c1c(C2=O)c(O)cc(O)c1
561	SBEH-16	Oc1cc(O)cc(C)c1C(=O)C
562	SBDM-114	Oc1cc(O)cc2c1[C@@H](C[C@@H](O)C2)C
563	R-NPG-14.cdx	Oc1cc(O)ccc1\C=C(\N1C(=O)c2c(cccc2)C1=O)/C(O)=O
564	SBEH-11	Oc1ccc(cc1)C
565	SBM-002	Oc1ccc(cc1)CCC(O)=O
566	SBDM-M1	Oc1ccc(cc1)CCC(OC)=O
567	R-NPG-3.cdx	Oc1ccc(cc1)\C=C(\N1C(=O)c2c(cccc2)C1=O)/C(O)=O
568	SBEH-18	Oc1ccccc1C=O
569	R-NPG-27.cdx	Oc1ccccc1\C=C(\N1C(=O)c2c(cccc2)C1=O)/C(O)=O
570	Remdesivir	P(Oc1ccccc1)(OCC1OC(C#N)(C(O)C1O)c1n2N=CN=C(N)c2cc1)(=O)NC(C(OCC(CC)CC)=O)C
571	R-NPG-9.cdx	S(C)c1ccc(cc1)\C=C(\N1C(=O)c2c(cccc2)C1=O)/C(O)=O
572	N14.mol	S1(=O)N=C(Nc2c1cccc2)c1ccccc1
573	N16.mol	S1(=O)N=C(Nc2c1cccc2)c1ncccc1
574	N17.mol	S1(=O)N=C(Nc2cc(ccc12)C(F)(F)F)c1ccccc1
575	R-NPG-24.cdx	S1C(CCC1\C=C(\N1C(=O)c2c(cccc2)C1=O)/C(O)=O)C
576	RXN-6.cdx	S1CC(=Nn2c1nnc2-c1ccc(OC)cc1)c1c2c(ccc1)cccc2
577	RXN-3.cdx	S1CC(=Nn2c1nnc2-c1ccc(OC)cc1)c1ccc(F)cc1
578	RXN-1.cdx	S1CC(=Nn2c1nnc2-c1ccc(OC)cc1)c1ccc(OC)cc1
579	RXN-7.cdx	S1CC(=Nn2c1nnc2-c1ccc(OC)cc1)c1ccc([N+](=O)[O-])cc1
580	RXN-5.cdx	S1CC(=Nn2c1nnc2-c1ccc(OC)cc1)c1ccc(cc1)-c1ccccc1
581	RXN-4.cdx	S1CC(=Nn2c1nnc2-c1ccc(OC)cc1)c1ccc(cc1)C
582	N-6.cdx	S1CC(=Nn2c1nnc2-c1cccnc1)c1c2c(ccc1)cccc2
583	N-3.cdx	S1CC(=Nn2c1nnc2-c1cccnc1)c1ccc(F)cc1
584	N-1.cdx	S1CC(=Nn2c1nnc2-c1cccnc1)c1ccc(OC)cc1
585	N-7.cdx	S1CC(=Nn2c1nnc2-c1cccnc1)c1ccc([N+](=O)[O-])cc1
586	N-5.cdx	S1CC(=Nn2c1nnc2-c1cccnc1)c1ccc(cc1)-c1ccccc1
587	N-4.cdx	S1CC(=Nn2c1nnc2-c1cccnc1)c1ccc(cc1)C
588	I-6.cdx	S1CC(=Nn2c1nnc2-c1ccncc1)c1c2c(ccc1)cccc2
589	I-3.cdx	S1CC(=Nn2c1nnc2-c1ccncc1)c1cc(F)ccc1
590	I-1.cdx	S1CC(=Nn2c1nnc2-c1ccncc1)c1cc(OC)ccc1
591	I-7.cdx	S1CC(=Nn2c1nnc2-c1ccncc1)c1cc([N+](=O)[O-])ccc1
592	I-5.cdx	S1CC(=Nn2c1nnc2-c1ccncc1)c1ccc(cc1)-c1ccccc1
593	I-4.cdx	S1CC(=Nn2c1nnc2-c1ccncc1)c1ccc(cc1)C
594	F-6.cdx	S1CC(=Nn2c1nnc2-c1occc1)c1c2c(ccc1)cccc2
595	F-3.cdx	S1CC(=Nn2c1nnc2-c1occc1)c1ccc(F)cc1
596	F-1.cdx	S1CC(=Nn2c1nnc2-c1occc1)c1ccc(OC)cc1
597	F-7.cdx	S1CC(=Nn2c1nnc2-c1occc1)c1ccc([N+](=O)[O-])cc1
598	F-5.cdx	S1CC(=Nn2c1nnc2-c1occc1)c1ccc(cc1)-c1ccccc1
599	F-4.cdx	S1CC(=Nn2c1nnc2-c1occc1)c1ccc(cc1)C
600	N13.mol	S1N=C(Nc2c1cccc2)c1ccccc1
601	N15.mol	S1N=C(Nc2c1cccc2)c1ncccc1
602	RXN-14.cdx	S1c2n(N=C1COc1ccc(F)cc1)c(nn2)-c1ccc(OC)cc1
603	NP-6.cdx	S1c2n(N=C1COc1ccc(F)cc1)c(nn2)-c1cccnc1
604	TP-6.cdx	S1c2n(N=C1COc1ccc(F)cc1)c(nn2)-c1ccncc1
605	FP-6.cdx	S1c2n(N=C1COc1ccc(F)cc1)c(nn2)-c1occc1
606	RXN-10.cdx	S1c2n(N=C1COc1ccc(O)cc1)c(nn2)-c1ccc(OC)cc1
607	NP-2.cdx	S1c2n(N=C1COc1ccc(O)cc1)c(nn2)-c1cccnc1
608	TP-2.cdx	S1c2n(N=C1COc1ccc(O)cc1)c(nn2)-c1ccncc1
609	FP-2.cdx	S1c2n(N=C1COc1ccc(O)cc1)c(nn2)-c1occc1
610	RXN-13.cdx	S1c2n(N=C1COc1ccc(OC)cc1)c(nn2)-c1ccc(OC)cc1

	mol	SMILES
611	NP-5.cdx	S1c2n(N=C1COc1ccc(OC)cc1)c(nn2)-c1cccnc1
612	TP-5.cdx	S1c2n(N=C1COc1ccc(OC)cc1)c(nn2)-c1ccncc1
613	FP-5.cdx	S1c2n(N=C1COc1ccc(OC)cc1)c(nn2)-c1occc1
614	RXN-12.cdx	S1c2n(N=C1COc1ccc(cc1)C)c(nn2)-c1ccc(OC)cc1
615	NP-4.cdx	S1c2n(N=C1COc1ccc(cc1)C)c(nn2)-c1cccnc1
616	FP-4.cdx	S1c2n(N=C1COc1ccc(cc1)C)c(nn2)-c1occc1
617	RXN-11.cdx	S1c2n(N=C1COc1ccccc1C)c(nn2)-c1ccc(OC)cc1
618	NP-3.cdx	S1c2n(N=C1COc1ccccc1C)c(nn2)-c1cccnc1
619	TP-3.cdx	S1c2n(N=C1COc1ccccc1C)c(nn2)-c1ccncc1
620	FP-3.cdx	S1c2n(N=C1COc1ccccc1C)c(nn2)-c1occc1
621	RXN-9.cdx	S1c2n(N=C1COc1ccccc1O)c(nn2)-c1ccc(OC)cc1
622	NP-1.cdx	S1c2n(N=C1COc1ccccc1O)c(nn2)-c1cccnc1
623	TP-1.cdx	S1c2n(N=C1COc1ccccc1O)c(nn2)-c1ccncc1
624	FP-1.cdx	S1c2n(N=C1COc1ccccc1O)c(nn2)-c1occc1
625	NT-8.cdx	S1c2n(N=C1Cc1ccc(F)cc1)c(nn2)-c1cccnc1
626	FT-6.cdx	S1c2n(N=C1Cc1ccc(F)cc1)c(nn2)-c1occc1
627	NT-9.cdx	S1c2n(N=C1Cc1ccc(OCC)cc1)c(nn2)-c1cccnc1
628	TT-7.cdx	S1c2n(N=C1Cc1ccc(OCC)cc1)c(nn2)-c1ccncc1
629	FT-9.cdx	S1c2n(N=C1Cc1ccc(OCC)cc1)c(nn2)-c1occc1
630	NT-8.cdx	S1c2n(N=C1c1[nH]ccc1)c(nn2)-c1cccnc1
631	TT-8.cdx	S1c2n(N=C1c1[nH]ccc1)c(nn2)-c1ccncc1
632	FT-7.cdx	S1c2n(N=C1c1[nH]ccc1)c(nn2)-c1occc1
633	NT-3.cdx	S1c2n(N=C1c1ccoc1)c(nn2)-c1cccnc1
634	TT-4.cdx	S1c2n(N=C1c1ccoc1)c(nn2)-c1ccncc1
635	FT-3.cdx	S1c2n(N=C1c1ccoc1)c(nn2)-c1occc1
636	NT-5.cdx	S1c2n(N=C1c1ccoc1C)c(nn2)-c1cccnc1
637	TT-5.cdx	S1c2n(N=C1c1ccoc1C)c(nn2)-c1ccncc1
638	FT-5.cdx	S1c2n(N=C1c1ccoc1C)c(nn2)-c1occc1
639	NT-4.cdx	S1c2n(N=C1c1occc1)c(nn2)-c1cccnc1
640	TT-3.cdx	S1c2n(N=C1c1occc1)c(nn2)-c1ccncc1
641	FT-4.cdx	S1c2n(N=C1c1occc1)c(nn2)-c1occc1
642	MI-79.mol	S=C(NC(=O)c1ccccc1)N\N=C(/C)\c1c2OC(=O)C=Cc2ccc1O
643	MI-59.mol	S=C(NC(=O)c1ccccc1)N\N=C(/C)\c1cc2CCOc2cc1
644	MI-219	S=C(NC(=O)c1ccccc1)N\N=C\c1ccc([N+](=O)[O-])cc1
645	MI-70.mol	S=C(NC1CCCCC1)N\N=C(/C)\c1c2OC(=O)C=Cc2ccc1O
646	MI-50.mol	S=C(NC1CCCCC1)N\N=C(/C)\c1cc2CCOc2cc1
647	MI-30.mol	S=C(NC1CCCCC1)N\N=C(/C)\c1cc2c(cc1)cccc2
648	MI-110.mol	S=C(NC1CCCCC1)N\N=C(/C)\c1nccnc1C
649	MI-190.mol	S=C(NC1CCCCC1)N\N=C\1/CCc2cc(OC)c(OC)cc/12
650	MI-210.mol	S=C(NC1CCCCC1)N\N=C\c1ccc([N+](=O)[O-])cc1
651	MI-37.mol	S=C(NCC)N\N=C(/C)\c1cc2c(cc1)cccc2
652	MI-117.mol	S=C(NCC)N\N=C(/C)\c1nccnc1C
653	MI-217	S=C(NCC)N\N=C\c1ccc([N+](=O)[O-])cc1
654	MI-21.mol	S=C(NCCN1CCOCC1)N\N=C(/C)\c1cc2c(cc1)cccc2
655	MI-34.mol	S=C(NCCc1ccccc1)N\N=C(/C)\c1cc2c(cc1)cccc2
656	MI-64.mol	S=C(N\N=C(/C)\c1c2OC(=O)C=Cc2ccc1O)N
657	MI-44.mol	S=C(N\N=C(/C)\c1cc2CCOc2cc1)N
658	MI-24.mol	S=C(N\N=C(/C)\c1cc2c(cc1)cccc2)N
659	MI-104.mol	S=C(N\N=C(/C)\c1nccnc1C)N
660	S23	S=C(N\N=C\1/CC[C@@]2(C3[C@](CCC2[C@]/1(C(OCc1ccccc1)=O)C)(C)[C@@]1(CC[C@@]2([C@H](C1=CC3=
661	MI-184.mol	S=C(N\N=C\1/CCc2cc(OC)c(OC)cc/12)N
662	MI-204.mol	S=C(N\N=C\c1ccc([N+](=O)[O-])cc1)N
663	MI-214.mol	S=C(Nc1c(cccc1C)C)N\N=C\c1ccc([N+](=O)[O-])cc1
664	MI-66.mol	S=C(Nc1c2c(ccc1)cccc2)N\N=C(/C)\c1c2OC(=O)C=Cc2ccc1O
665	MI-46.mol	S=C(Nc1c2c(ccc1)cccc2)N\N=C(/C)\c1cc2CCOc2cc1
666	MI-26.mol	S=C(Nc1c2c(ccc1)cccc2)N\N=C(/C)\c1cc2c(cc1)cccc2
667	MI-106.mol	S=C(Nc1c2c(ccc1)cccc2)N\N=C(/C)\c1nccnc1C
668	MI-186.mol	S=C(Nc1c2c(ccc1)cccc2)N\N=C\1/CCc2cc(OC)c(OC)cc/12
669	MI-206.mol	S=C(Nc1c2c(ccc1)cccc2)N\N=C\c1ccc([N+](=O)[O-])cc1
670	MI-35.mol	S=C(Nc1cc(F)ccc1)N\N=C(/C)\c1cc2c(cc1)cccc2
671	MI-65.mol	S=C(Nc1cc(OC)ccc1)N\N=C(/C)\c1c2OC(=O)C=Cc2ccc1O

	mol	SMILES
672	MI-45.mol	S=C(Nc1cc(OC)ccc1)N\N=C(/C)\c1cc2CCOc2cc1
673	MI-25.mol	S=C(Nc1cc(OC)ccc1)N\N=C(/C)\c1cc2c(cc1)cccc2
674	MI-216.mol	S=C(Nc1cc(cc(c1)C(F)(F)F)C(F)(F)F)N\N=C\c1ccc([N+](=O)[O-])cc1
675	MI-73.mol	S=C(Nc1ccc(F)cc1)N\N=C(/C)\c1c2OC(=O)C=Cc2ccc1O
676	MI-53.mol	S=C(Nc1ccc(F)cc1)N\N=C(/C)\c1cc2CCOc2cc1
677	MI-113.mol	S=C(Nc1ccc(F)cc1)N\N=C(/C)\c1nccnc1C
678	MI-193.mol	S=C(Nc1ccc(F)cc1)N\N=C\1/CCc2cc(OC)c(OC)cc/12
679	MI-213.mol	S=C(Nc1ccc(F)cc1)N\N=C\c1ccc([N+](=O)[O-])cc1
680	MI-23.mol	S=C(Nc1ccc([N+](=O)[O-])cc1)N\N=C(/C)\c1cc2c(cc1)cccc2
681	MI-103.mol	S=C(Nc1ccc([N+](=O)[O-])cc1)N\N=C(/C)\c1nccnc1C
682	MI-203.mol	S=C(Nc1ccc([N+](=O)[O-])cc1)N\N=C\c1ccc([N+](=O)[O-])cc1
683	MI-28.mol	S=C(Nc1ccc(cc1)C)N\N=C(/C)\c1cc2c(cc1)cccc2
684	MI-120.mol	S=C(Nc1ccc(cc1)CC)N\N=C(/C)\c1nccnc1C
685	MI-200.mol	S=C(Nc1ccc(cc1)CC)N\N=C\1/CCc2cc(OC)c(OC)cc/12
686	MI-220.cdx	S=C(Nc1ccc(cc1)CC)N\N=C\c1ccc([N+](=O)[O-])cc1
687	MI-72.mol	S=C(Nc1ccccc1)N\N=C(/C)\c1c2OC(=O)C=Cc2ccc1O
688	MI-52.mol	S=C(Nc1ccccc1)N\N=C(/C)\c1cc2CCOc2cc1
689	MI-32.mol	S=C(Nc1ccccc1)N\N=C(/C)\c1cc2c(cc1)cccc2
690	MI-112.mol	S=C(Nc1ccccc1)N\N=C(/C)\c1nccnc1C
691	MI-192.mol	S=C(Nc1ccccc1)N\N=C\1/CCc2cc(OC)c(OC)cc/12
692	MI-212.mol	S=C(Nc1ccccc1)N\N=C\c1ccc([N+](=O)[O-])cc1
693	MI-78.mol	S=C(Nc1ccccc1C)N\N=C(/C)\c1c2OC(=O)C=Cc2ccc1O
694	MI-58.mol	S=C(Nc1ccccc1C)N\N=C(/C)\c1cc2CCOc2cc1
695	MI-27.mol	S=C(Nc1ccccc1F)N\N=C(/C)\c1cc2c(cc1)cccc2
696	TRN.cdx	S=C1NN=C(N1N)c1cccnc1
697	TRI.cdx	S=C1NN=C(N1N)c1ccncc1
698	N4.mol	S=C=NCC(N1CCc2c([C@@H]1[C@H]1OCc3c1ccc(OC)c3OC)c(OC)c1OCOc1c2)c1cc2Cc3c(-c2cc1)cccc3
699	N1.mol	S=C=NCC(N1CCc2c([C@@H]1[C@H]1OCc3c1ccc(OC)c3OC)c(OC)c1OCOc1c2)c1ccc(F)cc1
700	N2.mol	S=C=NCC(N1CCc2c([C@@H]1[C@H]1OCc3c1ccc(OC)c3OC)c(OC)c1OCOc1c2)c1ccc(F)cc1
701	N5.mol	S=C=NCC(N1CCc2c([C@@H]1[C@H]1OCc3c1ccc(OC)c3OC)c(OC)c1OCOc1c2)c1ccc(cc1)C
702	N3.mol	S=C=NCC(N1CCc2c([C@@H]1[C@H]1OCc3c1ccc(OC)c3OC)c(OC)c1OCOc1c2)c1cccnc1
703	N8.mol	S=C=NCCCC(C)[C@@H]1[C@@H]2[C@H]([C@H]3[C@@H]([C@@H]4[C@@H](C[C@H](O)CC4)CC3)C[C@@H]2O)CC1
704	N6.mol	S=C=NCCCC(C)[C@H]1CC[C@H]2[C@H]3[C@H](C[C@H](O)[C@]12C)[C@@]1([C@@H](C[C@H](O)CC1)C[C@H]3
705	N10.mol	S=C=NCCC[C@@H](C)[C@H]1CC[C@H]2[C@H]3[C@H](CC[C@]12C)[C@@]1(C(C[C@@H](O)CC1)C[C@@H]3O)C
706	N9.mol	S=C=NCCC[C@@H](C)[C@H]1CC[C@H]2[C@H]3[C@H](CC[C@]12C)[C@@]1(C(C[C@H](O)CC1)C[C@H]3O)C
707	N7.mol	S=C=NCCC[C@H](C)[C@@H]1C2CC[C@]3(C)[C@]4(CC[C@@H](O)C[C@]4(C=C[C@@]3(C)[C@@]2(CC1)C)C)C
708	TS-108.cdx	S\1C=C(N(/C/1=N/N=C(C)C12CC3CC(C1)CC(C2)C3)c1c(cccc1C)C)c1ccccc1
709	TS-106.cdx	S\1C=C(N(/C/1=N/N=C(C)C12CC3CC(C1)CC(C2)C3)c1c2c(ccc1)cccc2)c1ccccc1
710	TS-105.cdx	S\1C=C(N(/C/1=N/N=C(C)C12CC3CC(C1)CC(C2)C3)c1ccccc1)c1ccccc1
711	TS-107.cdx	S\1C=C(N(/C/1=N/N=C(C)C12CC3CC(C1)CC(C2)C3)c1ccccc1C)c1ccccc1
712	TS-103.cdx	S\1C=C(N(/C/1=N/N=C(\C)/c1cc2NC(=O)COc2cc1)c1c(cccc1C)C)c1ccccc1
713	TS-102.cdx	S\1C=C(N(/C/1=N/N=C(\C)/c1cc2NC(=O)COc2cc1)c1cc(OC)ccc1)c1ccccc1
714	TS-101.cdx	S\1C=C(N(/C/1=N/N=C(\C)/c1cc2NC(=O)COc2cc1)c1ccccc1)c1ccccc1
715	TS-104.cdx	S\1C=C(N(/C/1=N/N=C(\C)/c1cc2NC(=O)COc2cc1)c1ccccc1C)c1ccccc1
716	TS-112.cdx	S\1C=C(N(/C/1=N/N=C(\C)/c1cc2OCCOc2cc1)c1c(cccc1C)C)c1ccccc1
717	TS-110.cdx	S\1C=C(N(/C/1=N/N=C(\C)/c1cc2OCCOc2cc1)c1c2c(ccc1)cccc2)c1ccccc1
718	TS-115.cdx	S\1C=C(N(/C/1=N/N=C/c1c2c([nH]c1)cccc2)C1CCCCC1)c1ccccc1
719	TS-116.cdx	S\1C=C(N(/C/1=N/N=C/c1c2c([nH]c1)cccc2)c1c(cccc1C)C)c1ccccc1
720	TS-113.cdx	S\1C=C(N(/C/1=N/N=C/c1c2c([nH]c1)cccc2)c1ccccc1)c1ccccc1
721	TS-111.cdx	S\1C=C(N(Cc2ccccc2)/C/1=N/N=C(\C)/c1cc2OCCOc2cc1)c1ccccc1
722	TS-114.cdx	S\1C=C(N(Cc2ccccc2)/C/1=N/N=C/c1c2c([nH]c1)cccc2)c1ccccc1
723	M-2.cdx	Sc1oc(nn1)-c1[nH]c2c(c1)cccc2
724	M-1.cdx	Sc1oc(nn1)-c1cc(OC)c(OC)c(OC)c1
725	M-15.cdx	Sc1oc(nn1)CCc1cc(OC)ccc1
726	M-7.cdx	Sc1oc(nn1)CCc1ccc(OC)cc1
727	M-9.cdx	Sc1oc(nn1)CCc1ccccc1OC
728	M-14.cdx	Sc1oc(nn1)Cc1cc(OC)ccc1
729	M-12.cdx	Sc1oc(nn1)Cc1ccc(OC)cc1
730	M-4.cdx	Sc1oc(nn1)Cc1ccc(cc1)C
731	M-6.cdx	Sc1oc(nn1)Cc1ccccc1F
732	M-13.cdx	Sc1oc(nn1)Cc1ccccc1OC

	mol	SMILES
733	Safavi-R32.mol	[Si](OC)(OC)(OC)CCCOCC1OC1
734	Safavi-R31.mol	[Si](OCC)(OCC)(OCC)CCCOCC1OC1
735	N-2	[Si](OCc1c(OC)c(OC)ccc1C(O)C1N(CCc2c1c(OC)c1OCOc1c2)C)(C(C)(C)C)(C)C
736	N-D-12c	[Si](OCc1c(OC)c(OC)ccc1C(O)C1N(CCc2c1c(OC)c1OCOc1c2)CC(c1ccc(F)cc1)c1ccc(F)cc1)(C(C)(C)C)
737	N-D-1c	[Si](OCc1c(OC)c(OC)ccc1C(O)C1N(CCc2c1c(OC)c1OCOc1c2)CC1CC1)(C(C)(C)C)(C)C
738	N-D-15c	[Si](OCc1c(OC)c(OC)ccc1C(O)C1N(CCc2c1c(OC)c1OCOc1c2)CCOc1ccccc1)(C(C)(C)C)(C)C
739	N-D-18c	[Si](OCc1c(OC)c(OC)ccc1C(O)C1N(CCc2c1c(OC)c1OCOc1c2)Cc1c2OCOc2ccc1)(C(C)(C)C)(C)C
740	N-D-11c	[Si](OCc1c(OC)c(OC)ccc1C(O)C1N(CCc2c1c(OC)c1OCOc1c2)Cc1c2c(cc3c1cccc3)cccc2)(C(C)(C)C)(C)
741	N-D-2c	[Si](OCc1c(OC)c(OC)ccc1C(O)C1N(CCc2c1c(OC)c1OCOc1c2)Cc1ccccc1)(C(C)(C)C)(C)C
742	S67	[Si](O[C@H]1C[C@]2([C@@H](CC[C@@]3(C2C(=O)C=C2[C@@H]4[C@@H](C)[C@@H](CC[C@@]4(CC[C@]23C)C
743	SK-10	c12c(c3c(c4CC[C@@]5(C(c4cc3)[C@@H](C)[C@@H](CC5)C)C)cc1)cccc2C
744	SK-12	c12c(c3c(c4c(c5cc(ccc5cc4)C)cc3)cc1)cccc2C
745	N12.mol	n1ccccc1/C(=N/c1ccccc1CCC)/N
746	1.cdx	o1c2nc3c(CCC(=O)C3(OC)C\C=C(\C)/C)c(OC)c2cc1
747	12.cdx	o1c2nc3c(ccc(O)c3C\C=C(\C)/C)c(OC)c2cc1
748	9.cdx	o1c2nc3c(ccc(OCC(O)C(O)(C)C)c3OC)c(OC)c2cc1
749	7.cdx	o1c2nc3c(cccc3OC)c(OC)c2cc1
750	MI-124.mol	s1c(/C(=N/NC(=S)N)/C)c(nc1C)C
751	MI-130.mol	s1c(/C(=N/NC(=S)NC2CCCCC2)/C)c(nc1C)C
752	MI-137.mol	s1c(/C(=N/NC(=S)NCC)/C)c(nc1C)C
753	MI-126.mol	s1c(/C(=N/NC(=S)Nc2c3c(ccc2)cccc3)/C)c(nc1C)C
754	MI-133.mol	s1c(/C(=N/NC(=S)Nc2ccc(F)cc2)/C)c(nc1C)C
755	MI-123.mol	s1c(/C(=N/NC(=S)Nc2ccc([N+](=O)[O-])cc2)/C)c(nc1C)C
756	MI-140.mol	s1c(/C(=N/NC(=S)Nc2ccc(cc2)CC)/C)c(nc1C)C
757	MI-132.mol	s1c(/C(=N/NC(=S)Nc2ccccc2)/C)c(nc1C)C
758	MI-150.mol	s1c(C)c(cc1C)/C(=N/NC(=S)NC1CCCCC1)/C
759	MI-157.mol	s1c(C)c(cc1C)/C(=N/NC(=S)NCC)/C
760	MI-153.mol	s1c(C)c(cc1C)/C(=N/NC(=S)Nc1ccc(F)cc1)/C
761	MI-143.mol	s1c(C)c(cc1C)/C(=N/NC(=S)Nc1ccc([N+](=O)[O-])cc1)/C
762	MI-160.mol	s1c(C)c(cc1C)/C(=N/NC(=S)Nc1ccc(cc1)CC)/C
763	MI-152.mol	s1c(C)c(cc1C)/C(=N/NC(=S)Nc1ccccc1)/C
764	32.cdx	s1c(ccc1C=O)-c1c2c(sc1C(OCCCC)=O)c1sc(C(OCCCC)=O)c(c1cc2)-c1sc(cc1)C=O
765	7.cdx	s1c2c(-c3c(NC2=O)cccc3)c2c1c1sc3c(-c4c(NC3=O)cccc4)c1cc2
766	31.cdx	s1c2c(ccc3c2sc(C(OCCCC)=O)c3-c2c(F)c(F)c(F)c(F)c2F)c(-c2c(F)c(F)c(F)c(F)c2F)c1C(OCCCC)=O
767	16.cdx	s1c2c(ccc3c2sc(C(OCCCC)=O)c3-c2c(F)cccc2F)c(-c2c(F)cccc2F)c1C(OCCCC)=O
768	14.cdx	s1c2c(ccc3c2sc(C(OCCCC)=O)c3-c2c(cccc2F)C(F)(F)F)c(-c2c(cccc2F)C(F)(F)F)c1C(OCCCC)=O
769	15.cdx	s1c2c(ccc3c2sc(C(OCCCC)=O)c3-c2cc(F)ccc2F)c(-c2cc(F)ccc2F)c1C(OCCCC)=O
770	12.cdx	s1c2c(ccc3c2sc(C(OCCCC)=O)c3-c2cc3c(cc2)cccc3)c(-c2cc3c(cc2)cccc3)c1C(OCCCC)=O
771	23.cdx	s1c2c(ccc3c2sc(C(OCCCC)=O)c3-c2ccc(F)cc2)c(-c2ccc(F)cc2)c1C(OCCCC)=O
772	20.cdx	s1c2c(ccc3c2sc(C(OCCCC)=O)c3-c2ccc(OC)cc2)c(-c2ccc(OC)cc2)c1C(OCCCC)=O
773	21.cdx	s1c2c(ccc3c2sc(C(OCCCC)=O)c3-c2ccc([N+](=O)[O-])cc2)c(-c2ccc([N+](=O)[O-])cc2)c1C(OCCCC)=
774	22.cdx	s1c2c(ccc3c2sc(C(OCCCC)=O)c3-c2ccc(cc2)C)c(-c2ccc(cc2)C)c1C(OCCCC)=O
775	27.cdx	s1c2c(ccc3c2sc(C(OCCCC)=O)c3-c2ccc(cc2)C=O)c(-c2ccc(cc2)C=O)c1C(OCCCC)=O
776	24.cdx	s1c2c(ccc3c2sc(C(OCCCC)=O)c3-c2cccc(C(F)(F)F)c2F)c(-c2cccc(C(F)(F)F)c2F)c1C(OCCCC)=O
777	11.cdx	s1c2c(ccc3c2sc(C(OCCCC)=O)c3-c2ccccc2)c(-c2ccccc2)c1C(OCCCC)=O
778	30.cdx	s1c2c(ccc3c2sc(C(OCCCC)=O)c3-c2ccccc2C#N)c(-c2ccccc2C#N)c1C(OCCCC)=O
779	29.cdx	s1c2c(ccc3c2sc(C(OCCCC)=O)c3-c2ccccc2C(=O)C)c(-c2ccccc2C(=O)C)c1C(OCCCC)=O
780	13.cdx	s1c2c(ccc3c2sc(C(OCCCC)=O)c3-c2ccccc2C(F)(F)F)c(-c2ccccc2C(F)(F)F)c1C(OCCCC)=O
781	6.cdx	s1c2c(ccc3c2sc(C(OCCCC)=O)c3-c2ccccc2N)c(-c2ccccc2N)c1C(OCCCC)=O
782	4.cdx	s1c2c(ccc3c2sc(C(OCCCC)=O)c3-c2ccccc2O)c(-c2ccccc2O)c1C(OCCCC)=O
783	5.cdx	s1c2c(ccc3c2sc(C(OCCCC)=O)c3-c2ccccc2O)c(-c2ccccc2O)c1C(OCCCC)=O
784	18.cdx	s1c2c(ccc3c2sc(C(OCCCC)=O)c3-c2ccccc2O)c(-c2ccccc2O)c1C(OCCCC)=O
785	19.cdx	s1c2c(ccc3c2sc(C(OCCCC)=O)c3-c2ccccc2OC)c(-c2ccccc2OC)c1C(OCCCC)=O
786	33.cdx	s1c2c(ccc3c2sc(C(OCCCC)=O)c3-c2ccccc2OC)c(-c2ccccc2OC)c1C(OCCCC)=O
787	26.cdx	s1c2c(ccc3c2sc(C(OCCCC)=O)c3-c2oc(cc2)C=O)c(-c2oc(cc2)C=O)c1C(OCCCC)=O
788	IN-3,4-DM-B.cdx	s1c2c(nc1N\N=C/c1cc(OC)ccc1OC)cccc2
789	IN-PDMA-B.cdx	s1c2c(nc1N\N=C/c1ccc(N(C)C)cc1)cccc2
790	IN-HB.cdx	s1c2c(nc1N\N=C/c1ccc(O)cc1)cccc2
791	IN-2,4-DH-B.cdx	s1c2c(nc1N\N=C/c1ccc(O)cc1O)cccc2
792	IN-PA.cdx	s1c2c(nc1N\N=C/c1ccc(OC)cc1)cccc2
793	IN-NB.cdx	s1c2c(nc1N\N=C/c1ccc([N+](=O)[O-])cc1)cccc2

	mol	SMILES
794	IN-MT.cdx	s1c2c(nc1N\N=C/c1ccc(cc1)C(OC)=O)cccc2
795	INB.cdx	s1c2c(nc1N\N=C/c1ccccc1)cccc2
796	IN-OA.cdx	s1c2c(nc1N\N=C/c1ccccc1OC)cccc2
797	IN-P-2CD.cdx	s1c2c(nc1N\N=C/c1ncccc1)cccc2
798	INT.cdx	s1c2c(nc1N\N=C\c1ccc(cc1)\C=N\Nc1sc3c(n1)cccc3)cccc2
799	9.cdx	s1c2c3c(-c4ccc(-c5c6c(sc5C(OCCCC)=O)c5sc(c(-c7ccc(-c8c(c2sc8C(OCCCC)=O)cc3)cc7)c5cc6)C(OC
800	10.cdx	s1c2c3c(-c4sc(-c5c6c(sc5C(OCCCC)=O)c5sc(c(-c7sc(-c8c(c2sc8C(OCCCC)=O)cc3)cc7)c5cc6)C(OCCC
801	28.cdx	s1cccc1-c1c2c(sc1C(OCCCC)=O)c1sc(C(OCCCC)=O)c(c1cc2)-c1sccc1
802	N18.mol	s1cccc1C=1Nc2c(SN=1)cccc2
803	Ritonavir	s1cncc1COC(=O)NC(Cc1ccccc1)C(O)CC(NC(=O)C(NC(=O)N(Cc1nc(sc1)C(C)C)C)C(C)C)Cc1ccccc1
804	Baicalein.cdx	O1c2c(c(O)c(O)c(O)c2)C(=O)C=C1c1ccccc1
805	Boceprevir .cdx	O=C(N[C@H]([C@@H](O)C(=O)N)CC1CCC1)[C@H]1N(C[C@@]2(C)[C@]1(C)C2(C)C)C(=O)[C@@H](NC(=O)NC(
806	Carmofur.cdx	FC1CN(C(=O)NCCCCCC)C(=O)NC1=O
807	Disulfiram.cdx	S(SC(=S)N(CC)CC)C(=S)N(CC)CC
808	Ebselen.cdx	[Se]1N(C(=O)c2c1cccc2)c1ccccc1
809	GC376.cdx	S(O)(=O)(=O)[C@H](O)[C@@H](NC(=O)[C@@H](NC(OCc1ccccc1)=O)CC(C)C)C[C@@H]1CCNC1=O
810	Masitinib.cdx	s1cc(nc1Nc1cc(NC(=O)c2ccc(cc2)CN2CCN(CC2)C)ccc1C)-c1cccnc1
811	N3.cdx	o1nc(cc1C)C(=O)N[C@H](C(=O)N[C@@H](C(C)C)C(=O)N[C@@H](CC(C)C)C(=O)N[C@H](\C=C\C(OCc1ccccc
812	O6K.cdx	O=C1N(C=CC=C1NC(OC(C)(C)C)=O)[C@@H](CC1CC1)C(=O)N[C@H]([C@@H](O)C(=O)NCc1ccccc1)C[C@@H]1C
813	px-12.cdx	S(SC(CC)C)c1[nH]ccn1
814	Shikonin.cdx	Oc1c2c(C(=O)CC([C@H](O)C\C=C(\C)/C)C2=O)c(O)cc1
815	Telaprevir.cdx	O=C(N[C@H](C(=O)N[C@@H](C(C)(C)C)C(=O)N1CC2C(CCC2)[C@H]1C(=O)N[C@H]([C@@H](O)C(=O)NC1CC1)
816	Tideglusib.cdx	S1N(c2c3c(ccc2)cccc3)C(=O)N(Cc2ccccc2)C1=O
817	ued.cdx	O=C1NCC[C@H]1C[C@H](NC(=O)[C@@H](NC(OCc1ccccc1)=O)CC(C)C)CO
818	X77.cdx	O=C(N([C@@H](C(=O)NC1CCCCC1)c1cccnc1)c1ccc(cc1)C(C)(C)C)c1nc[nH]c1
