# Supplementary material for: In Silico Prediction of Novel Inhibitors of SARS-CoV-2 Main Protease through Structure-Based Virtual Screening and Molecular Dynamic Simulation
Source: Pharmaceuticals (Basel). 2021 Sep 3;14(9):896. doi: 10.3390/ph14090896 (PMC8471057; doi:10.3390/ph14090896)
Supplement: Supplementary file 1 [file pharmaceuticals-14-00896-s001.zip › SI.pdf]

Supporting Information

In-silico Prediction of Novel Inhibitors of SARS-CoV-2 Main Protease through Structure-based Virtual Screening and Molecular Dynamic Simulation

Sobia Ahsan Halim[1,*], Muhammad Waqas[1,2], Ajmal Khan[1], and Ahmed Al-Harrasi[1,*]

[1]Natural and Medical Sciences Research Center, University of Nizwa, Birkat Al-Mouz, Nizwa 616, Sultanate of Oman

[2]Department of Biotechnology and Genetic Engineering, Hazara University Mansehra Pakistan

*Correspondence: sobia_halim@unizwa.edu.om (SAH); aharrasi@unizwa.edu.om (A.A.-H.)

Contents:

Table S1. Re-docking Results of MOE
Table S2. The cross-docking analysis of MOE
Calculation of Enrichment Factor (EF) and %EF
Table S3. Percent (%) Enrichment Factor and AUC of VS
Figure S1. The Receiver Operating Characteristic (ROC) curve of MOE on eight proteins
Table S4. Docking scores and rank of selected Hits
Figure S2. The 2D-structures of Selected Hits (1-30)
Table S5. Physicochemical Properties of Selected Hits (1-30)
Table S6. Solubility of Selected Hits (1-30)
Table S7. Pharmacokinetic properties of selected Hits
Table S8. Drug likeness and Medicinal properties of selected Hits (1-30)
Table S9. The ADMET results of admetSAR server
Table S10. Interaction Analysis of Selected Hits (1-30)
Table S11. Protein-ligand complexes used in Re-docking and Cross-docking

Table S1. Re-docking Results of MOE

S. No.	PDB IDs	Resolution (Å)	RMSD (Å)	S. No.	PDB IDs	Resolution (Å)	RMSD (Å)
1	6M2N	2.2	0.29	11	6Y2F	1.95	2.33
2	6W79	1.46	0.67	12	6ZRT	2.1	2.85
3	5R81	1.95	0.69	13	6WNP	1.44	2.9
4	5R7Y	1.65	0.71	14	7JU7	1.6	2.91
5	5R84	1.83	0.79	15	6WTK	2	2.93
6	5RE4	1.88	1.08	16	5R80	1.93	2.96
7	5R83	1.58	1.16	17	5R82	1.31	4.24
8	6W63	2.1	1.17	18	6LU7	2.16	5.27
9	5R7Z	1.59	1.39	19	7BQY	1.7	7.42
10	5RE9	1.72	1.94	20	7BRR	1.35	7.45

Table S2. The cross-docking analysis of MOE

Proteins	The ranks of Ligands in their cognate proteins																			
	6Y2F	6WTK	6W79	7BQY	6ZRT	7JU7	6LU7	6W63	7BRR	6WNP	6M2N	5R84	5R81	5R7Z	5R7Y	5RE9	5R80	5R83	5RE4	5R82
**6Y2F**	1	5	5	3	1	4	7	4	2	1	2	1	3	3	4	2	1	4	2	3
**6WTK**	4	1	9	6	5	10	6	8	4	5	9	9	6	9	7	6	4	8	3	8
**6W79**	8	6	1	5	6	6	5	5	8	4	9	5	14	7	5	1	8	5	9	5
**7BQY**	2	3	6	1	4	1	1	1	3	2	3	4	4	2	1	5	7	2	4	1
**6ZRT**	3	9	8	4	2	8	2	6	6	6	5	8	20	4	3	3	9	3	1	12
**7JU7**	9	10	13	10	10	2	8	10	7	9	4	10	1	5	8	17	3	10	8	6
**6LU7**	7	2	4	2	3	3	3	2	1	3	1	6	2	1	2	4	5	1	6	2
**6W63**	6	7	3	9	8	7	4	3	10	8	7	2	19	8	10	7	6	7	14	7
**7BRR**	5	4	7	7	7	5	10	9	5	10	6	3	5	6	6	9	2	6	5	4
**6WNP**	10	8	2	8	9	9	9	7	9	7	10	7	18	10	9	8	10	9	18	9
**6M2N**	12	11	12	14	12	13	11	12	14	14	11	13	10	12	17	10	11	13	7	11
**5R84**	11	13	11	11	15	12	12	13	13	11	12	12	8	16	16	12	13	14	12	10
**5R81**	16	14	18	17	16	17	13	15	17	16	16	18	13	14	15	15	18	17	15	18
**5R7Z**	15	15	15	15	14	14	18	16	16	15	15	14	9	13	12	11	14	11	10	16
**5R7Y**	18	18	16	18	17	15	15	18	18	17	17	17	12	18	14	13	17	16	17	14
**5RE9**	14	12	10	12	11	11	14	11	11	13	13	15	7	11	11	14	12	12	11	13
**5R80**	17	17	17	16	18	18	17	17	15	19	18	16	15	15	18	18	15	18	16	17
**5R83**	13	16	14	13	13	16	16	14	12	12	14	11	11	17	13	16	16	15	13	15
**5RE4**	20	19	19	19	20	19	20	19	19	20	19	19	16	20	19	20	19	20	19	19
**5R82**	19	20	20	20	19	20	19	20	20	18	20	20	17	19	20	19	20	19	20	20

The results are shown in three-color scheme, where green represents high rank, low and moderate ranks are shown by red and yellow colours, respectively.

Calculation of Enrichment Factor (EF) and %EF

EF and %EF were calculated by equations 1 and 2. Success was declared when 50% of the known inhibitors were ranked in top 50 or top 100 position of screened library in each protein.

Enrichment Factor = ((HITS sampled)/(HITS Total))/((N sampled)/(N Total)) Eq. 1

% Enrichment Factor = (Enrichment Factor)/(Ideal Enrichment Factor) x 100 Eq. 2

HIT sampled and HITS total corresponds to the number of active inhibitors found in top N sampled ligands of docked library, and total number of active compounds, respectively. N sampled and N total are number of ligands in the docked database to be examined, and total number of compounds in the database, respectively.

The optimal threshold to decide the accuracy of virtual screening was set by the following criteria: if all the known inhibitors are successfully identified in top 50 or 100 compounds in screened database, then the ideal EF would be 18, and 9, respectively.

Table S3. Percent (%) Enrichment Factor and AUC of VS

Library Screened	% Enrichment Factor							
	6Y2F	6WTK	6W79	7BQY	6ZRT	7JU7	6LU7	6W63
In top 50	20	27	53	33	20	13	13	27
In top 100	33	47	73	53	53	60	27	60
In top 250	67	73	100	73	80	73	87	67
	AUC-ROC							
	6Y2F	6WTK	6W79	7BQY	6ZRT	7JU7	6LU7	6W63
	0.80	0.80	0.90	0.83	0.84	0.79	0.81	0.83

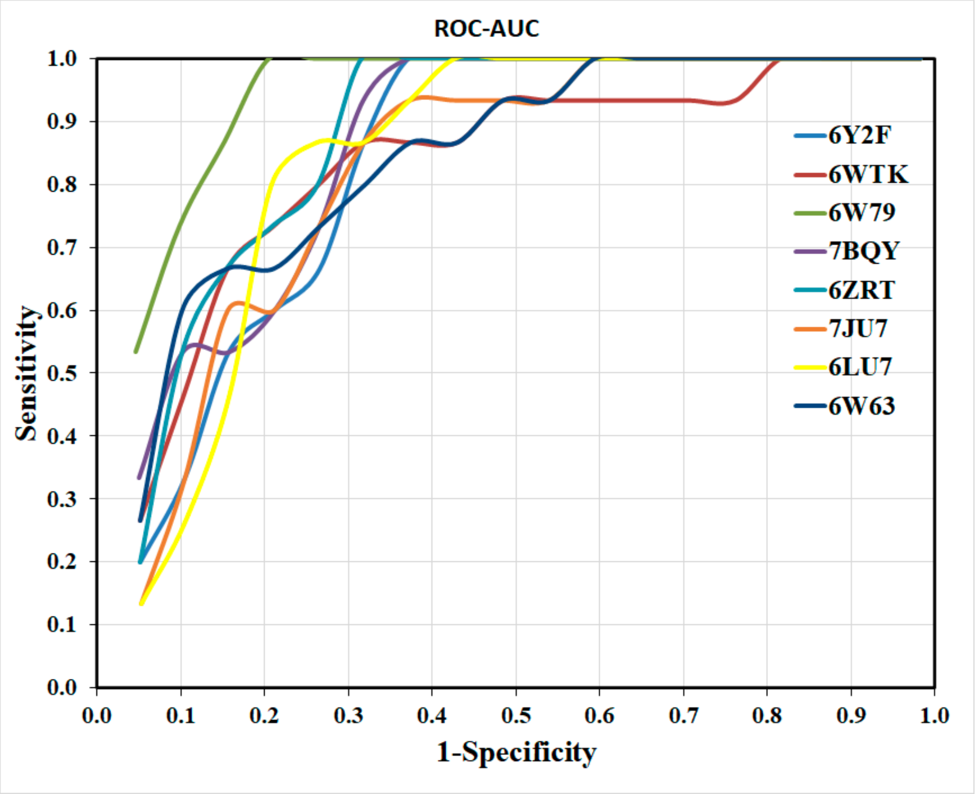

Figure S1. The Receiver Operating Characteristic (ROC) curve of MOE on eight proteins

Table S4. Docking scores and rank of selected Hits

Compounds	Compound Codes	6W63		6W79		6WTK		6Y2F		6LU7		7JU7		7BQY		6ZRT	
		Rank	Score	Rank	Score	Rank	Score	Rank	Score	Rank	Score	Rank	score	Rank	Score	Rank	Score
1	JAOETH35	19	-13.46	18	-13.94	2	-16.95	6	-15.22	1	-16.68	67	-12.6	4	-15.3	8	-14.15
2	QTEM-10	1	-16.28	1	-16.08	6	-14.98	2	-15.86	3	-16.2	1	-15.55	9	-14.76	1	-15.34
3	JAOMET4	10	-13.96	14	-14.31	7	-14.76	4	-15.47	4	-15.93	4	-15.08	6	-15.09	2	-15.1
4	QTME-16	3	-15.03	6	-14.69	1	-17.52	5	-15.35	6	-15.15	5	-14.71	44	-13.16	11	-14.06
5	JAOETH-90	13	-13.79	8	-14.48	4	-15.31	9	-14.7	7	-15.11	55	-12.74	7	-14.97	13	-13.82
6	SBEH-40	7	-14.55	13	-14.36	32	-13.55	36	-13.4	8	-15.04	10	-14.04	21	-13.96	37	-13.01
7	QTME-12	26	-13.31	21	-13.59	41	-13.27	25	-13.69	10	-14.8	16	-13.64	23	-13.95	40	-13
8	SBME-25	5	-14.85	10	-14.44	29	-13.65	8	-14.73	11	-14.75	2	-15.299	3	-15.9	5	-14.48
9	SBEH-60	4	-14.85	7	-14.61	11	-14.37	10	-14.61	15	-14.63	15	-13.74	2	-16.02	50	-12.87
10	QTME-14	11	-13.96	3	-15.75	10	-14.41	3	-15.73	19	-14.26	60	-12.71	31	-13.48	23	-13.38
11	SBME-P5	68	-12.19	26	-13.43	9	-14.67	30	13.56	45	-13.14	3	-15.11	70	-12.74	14	-13.65
12	SB-1	91	-12.02	4	-15.73	68	-12.74	37	-13.38	72	-12.75	99	-12.3	43	-13.17	15	-13.58
13	R-NPG-14	16	-13.64	67	-12.55	25	-13.76	49	-13.19	88	-12.59	61	-12.71	20	-13.96	36	-13.03
14	QTH-90	6	-14.8	5	-15.11	5	-15.23	1	-16.36	13	-14.7	161	-11.97	1	-16.11	22	-13.41
15	SBME-P3	99	-11.89	83	-12.37	40	-13.27	148	-12.19	24	-13.89	26	-13.28	73	-12.66	44	-12.94
16	5.cdx	70	-12.18	25	-13.48	79	-12.63	141	-12.23	44	-13.15	18	-13.55	25	-13.71	51	-12.86
17	JCDACH90	38	-12.79	72	-12.51	36	-13.43	11	-14.54	47	-13.11	188	-11.78	19	-14.06	19	-13.47
18	R-NPG-3	36	-12.8	37	-13.05	35	-13.49	177	-12.07	96	-12.53	90	-12.36	14	-14.15	10	-14.14
19	N-D-12A	117	-11.75	122	-12.04	18	-14.09	99	-12.54	14	-14.64	102	-12.27	17	-14.13	6	-14.23
20	R-NPG-21	25	-13.38	34	-13.15	117	-12.32	105	-12.52	21	-14.16	7	-14.48	185	-11.899	34	-13.05
21	S5-5	59	-12.34	65	-12.56	8	-14.71	14	-14.2	42	-13.22	22	-13.37	181	-11.93	172	-11.78
22	AM-12	33	-12.99	102	-12.2	26	-13.71	156	-12.14	55	-12.99	43	-12.92	127	-12.23	179	-11.74
23	N-D-12B	60	-12.33	129	-11.98	22	-13.88	12	-14.38	63	-12.897	194	-11.7	109	-12.35	203	-11.65
24	F-14	107	-11.81	189	-11.64	143	-12.16	80	-12.67	64	-12.84	12	-14.03	176	-11.95	83	-12.37
25	6D	200	-11.3	48	-12.76	184	-11.91	194	-11.92	70	-12.796	58	-12.72	41	-13.188	210	-11.63
26	6JA	186	-11.37	166	-11.76	72	-12.69	50	-13.17	36	-13.39	109	-12.22	63	-12.779	66	-12.56
27	6FA	24	-13.38	143	-11.89	114	-12.33	24	-13.72	99	-12.48	88	-12.36	54	-13.01	108	-12.16
28	RXN-24	47	-12.64	78	-12.46	94	-12.48	95	-12.59	77	-12.698	247	-11.46	30	-13.53	67	-12.54
29	SBME-P6	54	-12.43	87	-12.34	12	-14.32	66	-12.86	22	-13.95	248	-11.28	36	-13.34	4	-14.57
30	6MA	21	-13.46	40	-12.95	244	-11.7	53	-13.14	83	-12.64	8	-14.45	27	-13.69	55	-12.79

The rank and score of compounds at top-100 position are highlighted in green colour, while compounds from top-100 to top-200 and from top-200 to top-250 position are highlighted in yellow and orange colours, respectively.

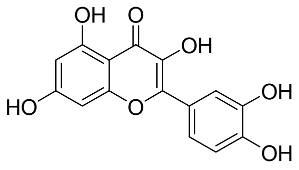

(1)
2-(3,4-dihydroxyphenyl)-3,5,7-trihydroxy-4H-chromen-4-one

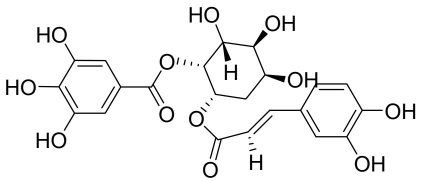

(2)
1R,2R,3S,4S,6S)-6-((E)-3-(3,4-dihydroxyphenyl)acryloyloxy)-2,3,4-trihydroxycyclohexyl 3,4,5-trihydroxybenzoate

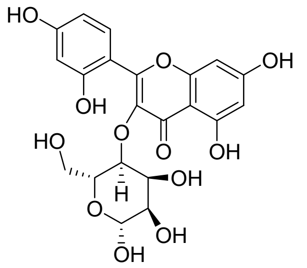

(3)
2-(2,4-dihydroxyphenyl)-5,7-dihydroxy-3-((2R,3S,4S,5R,6R)-4,5,6-trihydroxy-2-(hydroxymethyl)-tetrahydro-2H-pyran-3-yloxy)-4H-chromen-4-one

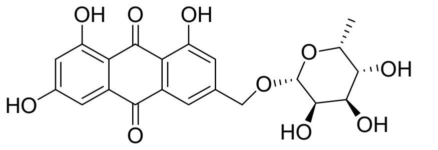

(4)
1,3,8-trihydroxy-6-(((2R,3R,4R,5R,6R)-3,4,5-trihydroxy-6-methyl-tetrahydro-2H-pyran-2-yloxy)methyl)anthracene-9,10-dione

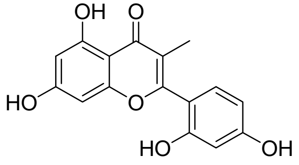

(5)
2-(2,4-dihydroxyphenyl)-5,7-dihydroxy-3-methyl-4H-chromen-4-one

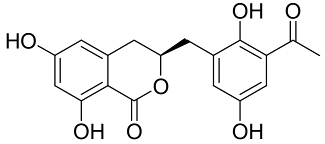

(6)
(S)-3-(3-acetyl-2,5-dihydroxybenzyl)-6,8-dihydroxy-3,4-dihydroisochromen-1-one

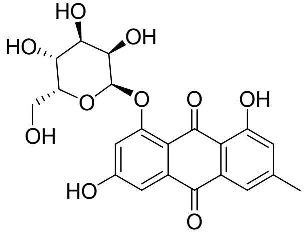

(7)
1,6-dihydroxy-3-methyl-8-((2R,3R,4R,5R,6R)-3,4,5-trihydroxy-6-(hydroxymethyl)-tetrahydro-2H-pyran-2-yloxy)anthracene-9,10-dione

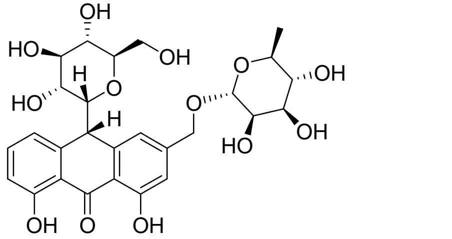

(8)
(S)-4,5-dihydroxy-9-((2R,3R,4R,5S,6R)-3,4,5-trihydroxy-6-(hydroxymethyl)-tetrahydro-2H-pyran-2-yl)-2-(((2R,3R,4R,5R,6S)-3,4,5-trihydroxy-6-methyl-tetrahydro-2H-pyran-2-yloxy)methyl)anthracen-10(9H)-one

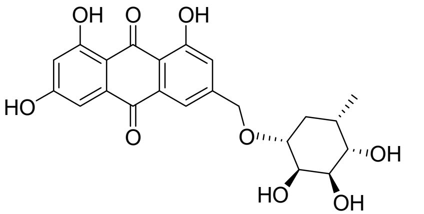

(9)
1,3,8-trihydroxy-6-(((1R,2R,3R,4S,5S)-2,3,4-trihydroxy-5-methylcyclohexyloxy)methyl)anthracene-9,10-dione

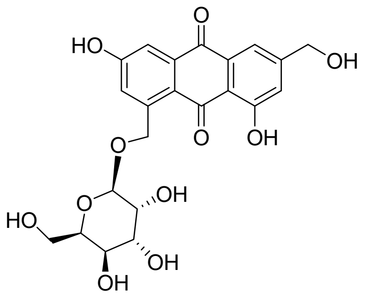

(10)
1,6-dihydroxy-3-(hydroxymethyl)-8-(((2R,3R,4R,5R,6R)-3,4,5-trihydroxy-6-(hydroxymethyl)-tetrahydro-2H-pyran-2-yloxy)methyl)anthracene-9,10-dione

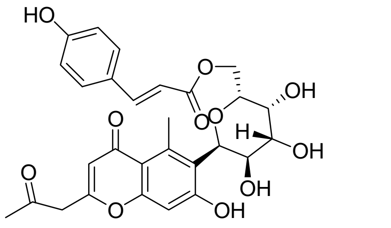

(11)
(E)-((2R,3R,4R,5R,6R)-3,4,5-trihydroxy-6-(7-hydroxy-5-methyl-4-oxo-2-(2-oxopropyl)-4H-chromen-6-yl)-tetrahydro-2H-pyran-2-yl)methyl 3-(4-hydroxyphenyl)acrylate

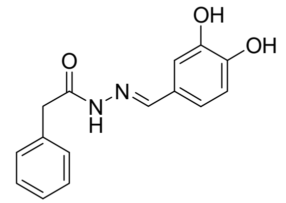

(12)
(E)-N'-(3,4-dihydroxybenzylidene)-2-phenylacetohydrazide

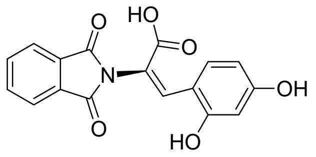

(13)
(E)-3-(2,4-dihydroxyphenyl)-2-(1,3-dioxoisoindolin-2-yl)acrylic acid

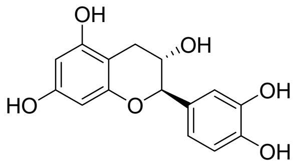

(14)
(2R,3S)-2-(3,4-dihydroxyphenyl)-3,4-dihydro-2H-chromene-3,5,7-triol

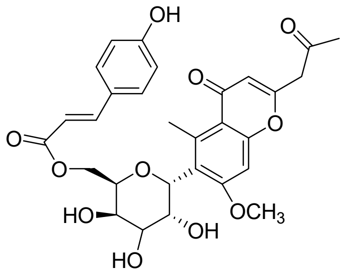

(15)
(E)-((2R,3R,5R,6R)-3,4,5-trihydroxy-6-(7-methoxy-5-methyl-4-oxo-2-(2-oxopropyl)-4H-chromen-6-yl)-tetrahydro-2H-pyran-2-yl)methyl 3-(4-hydroxyphenyl)acrylate

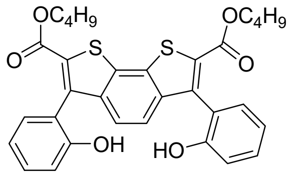

(16)
4,11-dibutyl 5,10-bis(2-hydroxyphenyl)-3,12-dithiatricyclo[7.3.0.$0^{2,6}$]dodeca-1,4,6,8,10-pentaene-4,11-dicarboxylate

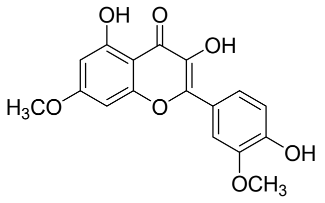

(17)
3,5-dihydroxy-2-(4-hydroxy-3-methoxyphenyl)-7-methoxy-4H-chromen-4-one

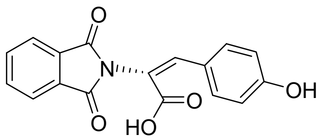

(18)
(E)-2-(1,3-dioxoisoindolin-2-yl)-3-(4-hydroxyphenyl)acrylic acid

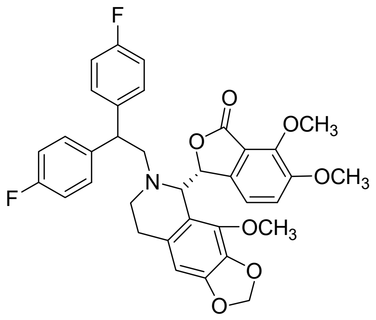

(19)
(R)-3-((R)-6-(2,2-bis(4-fluorophenyl)ethyl)-4-methoxy-5,6,7,8-tetrahydro-[1,3]dioxolo[4,5-g]isoquinolin-5-yl)-6,7-dimethoxyisobenzofuran-1(3H)-one

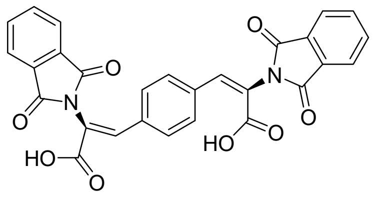

(20)
3-{4-[2-carboxy-2-(1,3-dioxo-2,3-dihydro-1H-isoindol-2-yl)eth-1-en-1-yl]phenyl}-2-(1,3-dioxo-2,3-dihydro-1H-isoindol-2-yl)prop-2-enoic acid

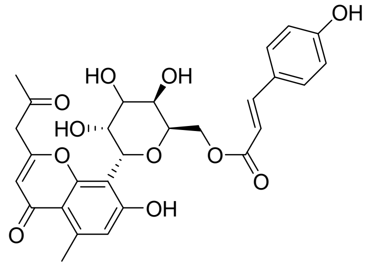

(21)
(E)-((2R,3R,5R,6R)-3,4,5-trihydroxy-6-(7-hydroxy-5-methyl-4-oxo-2-(2-oxopropyl)-4H-chromen-8-yl)-tetrahydro-2H-pyran-2-yl)methyl 3-(4-hydroxyphenyl)acrylate

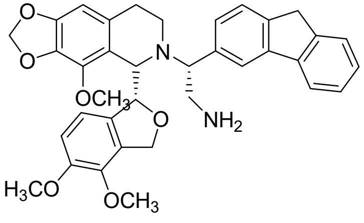

(22)
(R)-2-((R)-5-((R)-4,5-dimethoxy-1,3-dihydroisobenzofuran-1-yl)-4-methoxy-7,8-dihydro-[1,3]dioxolo[4,5-g]isoquinolin-6(5H)-yl)-2-(9H-fluoren-3-yl)ethanamine

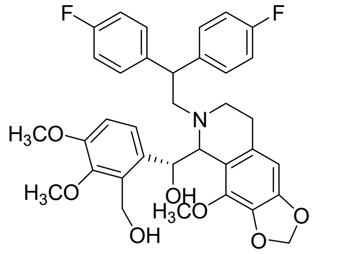

(23)
(R)-{6-[2,2-bis(4-fluorophenyl)ethyl]-4-methoxy-2H,5H,6H,7H,8H-[1,3]dioxolo[4,5-g]isoquinolin-5-yl}[2-(hydroxymethyl)-3,4-dimethoxyphenyl]methanol

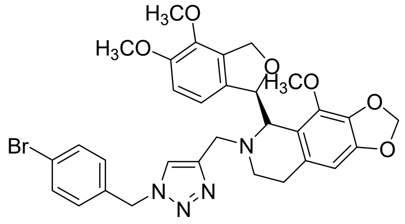

(24)
(S)-6-((1-(4-bromobenzyl)-1H-1,2,3-triazol-4-yl)methyl)-4-methoxy-5-((R)-5-methoxy-4-methyl-1,3-dihydroisobenzofuran-1-yl)-5,6,7,8-tetrahydro-[1,3]dioxolo[4,5-g]isoquinoline

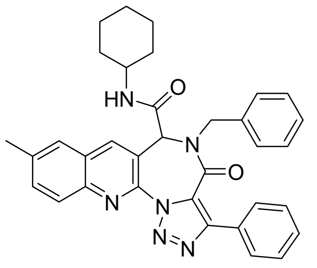

(25)
8-benzyl-N-cyclohexyl-14-methyl-7-oxo-5-phenyl-2,3,4,8,18-pentaazatetracyclo[8.8.0.$0^{2,6}$.$0^{12,17}$]octadeca-1(10),3,5,11,13,15,17-heptaene-9-carboxamide

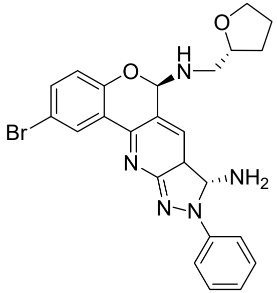

(26)
(9R,13R)-4-bromo-N9-{[(2R)-oxolan-2-yl]methyl}-14-phenyl-8-oxa-14,15,17-triazatetracyclo[8.7.0.$0^{2,7}$.$0^{12,16}$]heptadeca-1(17),2,4,6,10,15-hexaene-9,13-diamine

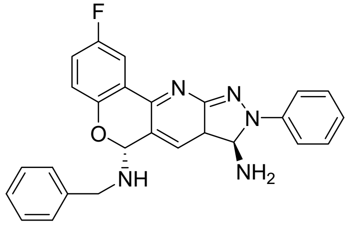

(27)
(9R,13R)-N9-benzyl-4-fluoro-14-phenyl-8-oxa-14,15,17-triazatetracyclo[8.7.0.$0^{2,7}$.$0^{12,16}$]heptadeca-1(17),2(7),3,5,10,15-hexaene-9,13-diamine

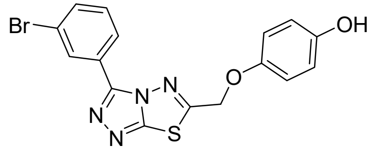

(28)
4-((3-(3-bromophenyl)-[1,2,4]triazolo[3,4-b][1,3,4]thiadiazol-6-yl)methoxy)phenol

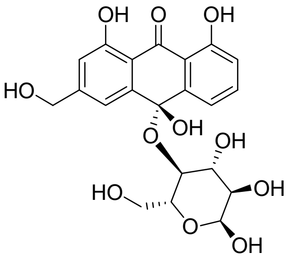

(29)
(S)-4,5,9-trihydroxy-2-(hydroxymethyl)-9-((2R,3S,4R,5R,6S)-4,5,6-trihydroxy-2-(hydroxymethyl)-tetrahydro-2H-pyran-3-yloxy)anthracen-10(9H)-one

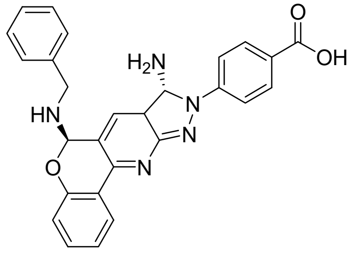

(30)
4-[(9R,13R)-13-amino-9-(benzylamino)-8-oxa-14,15,17-triazatetracyclo[8.7.0.$0^{2,7}$.$0^{12,16}$]heptadeca-1(17),2(7),3,5,10,15-hexaen-14-yl]benzoic acid

Figure S2. The chemical-structures of Selected Hits (1-30)

Table S5. Physicochemical Properties of Selected Hits (1-30)

Compound	Physicochemical Properties									
	Formula	MW (g.mol)	NHA	NAHA	Fraction Csp3	NRB	HBA	HBD	MR	TPSA (Å^2)
**1**	C15H10O7	302.24	22	16	0.00	1	7	5	78.04	131.36 s
**2**	C22H22O13	494.40	35	12	0.27	8	13	8	114.84	223.67
**3**	C21H20O12	464.38	33	16	0.29	4	12	8	110.16	210.51
**4**	C21H20O10	432.38	31	12	0.33	3	10	6	103.17	173.98
**5**	C21H20O11	448.38	32	16	0.29	3	11	7	109.00	190.28
**6**	C18H16O7	344.32	25	12	0.22	3	7	4	88.15	124.29
**7**	C21H20O10	432.38	31	12	0.33	3	10	6	102.91	173.98
**8**	C27H32O13	564.54	40	12	0.52	5	13	9	133.18	226.83
**9**	C21H20O10	432.38	31	12	0.33	3	10	6	103.17	173.98
**10**	C22H22O11	462.40	33	12	0.36	5	11	7	108.43	194.21
**11**	C28H28O11	540.52	39	16	0.32	8	11	5	138.24	183.96
**12**	C15H13BrN2O3	349.18	21	12	0.07	5	4	3	83.94	81.92
**13**	C17H11NO6	325.27	24	12	0.00	3	6	3	86.96	115.14
**14**	C15H14O6	290.27	21	12	0.20	1	6	5	74.33	110.38
**15**	C29H30O11	554.54	40	16	0.34	9	11	4	142.71	172.96
**16**	C169H23NO3	313.39	23	10	0.32	6	3	2	94.70	62.32
**17**	C17H14O7	330.29	24	16	0.12	3	7	3	86.97	109.36
**18**	C17H11NO5	309.27	23	12	0.00	3	5	2	84.93	94.91
**19**	C35H31F2NO7	615.62	45	24	0.29	8	10	0	163.57	75.69
**20**	C28H16N2O8	508.44	38	18	0.00	6	8	2	139.38	149.36
**21**	C28H28O11	540.52	39	16	0.32	8	11	5	138.24	183.96
**22**	C36H36N2O6	592.68	44	24	0.33	7	8	1	169.90	84.64
**23**	C35H35F2NO7	619.65	45	24	0.31	10	10	2	166.50	89.85
**24**	C31H31BrN4O6	635.51	42	23	0.35	8	9	0	160.97	89.33
**25**	C34H32N6O2	556.66	42	27	0.26	6	5	1	165.94	93.01
**26**	C24H24BrN5O2	494.38	32	12	0.33	4	6	2	137.21	84.84
**27**	C26H22FN5O	439.48	33	18	0.15	4	6	2	135.76	75.24
**28**	C16H11BrN4O2S	403.25	24	20	0.06	4	5	1	94.80	100.78
**29**	C21H22O11	450.39	32	12	0.38	4	11	8	104.09	197.37
**30**	C27H23N5O3	465.50	35	18	0.15	5	7	3	142.76	112.54

MW = molecular weight, NHA = number of heavy atoms, NAHA = number of aromatic heavy atoms, NRB = number of rotatable bonds, HBA = number of hydrogen bond acceptors, HBD = number of hydrogen bond donors, MR = molar refractivity, TPSA = topological polar surface area

Table S6. Solubility of Selected Hits (1-30)

Compounds	Lipophilicity	Water Solubility		
	Log $P_{o/w}$ (iLOGP)	Log *S* (ESOL)	S	Class
**1**	1.63	-3.16	2.11e-01 mg/ml ; 6.98e-04 mol/l	Soluble
**2**	2.29	-2.96	5.36e-01 mg/ml ; 1.08e-03 mol/l	Soluble
**3**	1.09	-2.30	2.34e+00 mg/ml ; 5.04e-03 mol/l	Soluble
**4**	2.18	-2.84	6.22e-01 mg/ml ; 1.44e-03 mol/l	Soluble
**5**	1.74	-2.94	5.11e-01 mg/ml ; 1.14e-03 mol/l	Soluble
**6**	2.14	-4.15	2.45e-02 mg/ml ; 7.11e-05 mol/l	MS
**7**	1.86	-3.18	2.84e-01 mg/ml ; 6.57e-04 mol/l	Soluble
**8**	0.25	-2.46	1.94e+00 mg/ml ; 3.44e-03 mol/l	Soluble
**9**	2.18	-2.84	6.22e-01 mg/ml ; 1.44e-03 mol/l	Soluble
**10**	1.39	-2.23	2.72e+00 mg/ml ; 5.89e-03 mol/l	Soluble
**11**	2.17	-3.26	2.95e-01 mg/ml ; 5.46e-04 mol/l	Soluble
**12**	2.24	-3.83	5.16e-02 mg/ml ; 1.48e-04 mol/l	Soluble
**13**	1.30	-3.23	1.91e-01 mg/ml ; 5.86e-04 mol/l	Soluble
**14**	1.33	-2.22	1.74e+00 mg/ml ; 5.98e-03 mol/l	Soluble
**15**	2.77	-3.48	1.82e-01 mg/ml ; 3.28e-04 mol/l	Soluble
**16**	3.14	-3.86	4.36e-02 mg/ml ; 1.39e-04 mol/l	MS
**17**	2.81	-3.56	9.04e-02 mg/ml ; 2.74e-04 mol/l	MS
**18**	1.40	-3.37	1.32e-01 mg/ml ; 4.27e-04 mol/l	Soluble
**19**	4.74	-7.51	1.92e-05 mg/ml ; 3.13e-08 mol/l	PS
**20**	2.03	-5.04	4.65e-03 mg/ml ; 9.15e-06 mol/l	PS
**21**	2.77	-3.26	2.95e-01 mg/ml ; 5.46e-04 mol/l	soluble
**22**	4.67	-6.43	2.20e-04 mg/ml ; 3.72e-07 mol/l	PS
**23**	4.73	-6.64	1.41e-04 mg/ml ; 2.28e-07 mol/l	PS
**24**	4.19	-6.23	3.71e-04 mg/ml ; 5.83e-07 mol/l	MS
**25**	4.19	-7.27	2.98e-05 mg/ml ; 5.36e-08 mol/l	PS
**26**	3.39	-4.95	5.50e-03 mg/ml ; 1.11e-05 mol/l	MS
**27**	3.47	-5.05	3.88e-03 mg/ml ; 8.82e-06 mol/l	MS
**28**	3.03	-5.06	3.50e-03 mg/ml ; 8.68e-06 mol/l	MS
**29**	1.25	-1.57	1.22e+01 mg/ml ; 2.70e-02 mol/l	VS
**30**	2.81	-3.32	2.20e-01 mg/ml ; 4.73e-04 mol/l	Soluble

MS = Moderately soluble, PS = Poorly soluble, VS = Very soluble

Table S7. Pharmacokinetic properties of selected Hits

Compounds	GIA	BBBP	P-gp Subs	CYP1A2 Inh	CYP2C19 Inh	CYP2C9 Inh	CYP2D6 Inh	CYP3A4 Inh	Log K_p (cm/s)
**1**	High	No	No	Yes	No	No	Yes	Yes	-7.05
**2**	Low	No	Yes	No	No	No	No	No	-8.94
**3**	Low	No	No	No	No	No	No	No	-9.71
**4**	Low	No	Yes	No	No	No	No	No	-8.67
**5**	Low	No	No	No	No	No	No	No	-8.86
**6**	High	No	No	No	No	No	Yes	No	-613
**7**	Low	No	Yes	No	No	No	No	No	-8.29
**8**	Low	No	No	No	No	No	No	No	-10.61
**9**	Low	No	Yes	No	No	No	No	No	-8.67
**10**	Low	No	No	No	No	No	No	No	-9.59
**11**	Low	No	No	No	No	No	No	No	-9.26
**12**	High	No	No	Yes	No	Yes	No	No	-6.48
**13**	High	No	No	No	No	No	No	No	-6.93
**14**	High	No	Yes	No	No	No	No	No	-7.82
**15**	Low	No	Yes	No	No	No	No	Yes	-9.11
**16**	High	Yes	No	No	Yes	Yes	No	No	-5.79
**17**	High	No	No	Yes	No	Yes	Yes	Yes	-6.76
**18**	High	No	No	No	No	No	No	No	-6.58
**19**	High	No	No	No	No	Yes	Yes	No	-5.57
**20**	Low	No	No	No	No	Yes	No	No	-7.04
**21**	Low	No	Yes	No	No	No	No	No	-9.26
**22**	High	No	No	No	No	No	No	Yes	-6.56
**23**	High	No	Yes	No	No	No	No	Yes	-6.44
**24**	High	No	Yes	No	No	Yes	Yes	Yes	-7.27
**25**	High	No	No	No	Yes	Yes	Yes	Yes	-5.30
**26**	High	No	Yes	No	Yes	Yes	Yes	Yes	-7.02
**27**	High	Yes	Yes	No	Yes	Yes	Yes	No	-6.33
**28**	High	No	No	Yes	Yes	Yes	No	No	-6.09
**29**	Low	No	No	No	No	No	No	No	-10.26
**30**	High	No	Yes	No	No	No	No	No	-8.52

GIA = Gastrointestinal absorption, BBBP = Blood brain barrier penetration, P-gp = P-glycoprotein, CYP = Cytochrome P450, Subs = Substrate, Inh = Inhibition, Log Kp = Skin permeation

Table S8. Drug likeness and Medicinal properties of selected Hits (1-30)

Compounds	Drug likeness						Medicinal Chemistry		
	Lipinski	Ghose	Veber	Egan	Muegge	BA	PAINS	Lead-likeness	SA
**1**	Yes	Yes	Yes	Yes	Yes	0.55	1 alert: catechol_A	Yes	3.23
**2**	2 violations: NorO>10, NHorOH>5	2 violations: MW>480, WLOGP<-0.4	1 violation: TPSA>140	1 violation: TPSA>131.6	3 violations: TPSA>150, H-acc>10, H-don>5	0.17	1 alert: catechol_A	2 violations: MW>350, Rotors>7	5.09
**3**	2 violations: NorO>10, NHorOH>5	1 violation: WLOGP<-0.4	1 violation: TPSA>140	1 violation: TPSA>131.6	3 violations: TPSA>150, H-acc>10, H-don>5	0.17	0 alert	1 violation: MW>350	5.38
**4**	1 violation: NHorOH>5	Yes	1 violation: TPSA>140	1 violation: TPSA>131.6	2 violations: TPSA>150, H-don>5	0.55	1 alert: quinone_A	1 violation: MW>350	4.96
**5**	2 violations: NorO>10, NHorOH>5	Yes	1 violation: TPSA>140	1 violation: TPSA>131.6	3 violations: TPSA>150, H-acc>10, H-don>5	0.17	0 alert	1 violation: MW>350	5.36
**6**	Yes	Yes	Yes	Yes	Yes	0.55	0 alert	Yes	3.48
**7**	1 violation: NH or OH>5	1 violation: WLOGP<-0.4	1 violation: TPSA>140	1 violation: TPSA>131.6	2 violations: TPSA>150, H-don>5	0.55	1 alert: quinone_A	1 violation: MW>350	5.06
**8**	3 violations: MW>500, NorO>10, NHorOH>5	4 violations: MW>480, WLOGP<-0.4, MR>130, #atoms>70	1 violation: TPSA>140	1 violation: TPSA>131.6	3 violations: TPSA>150, H-acc>10, H-don>5	0.17	0 alert	1 violation: MW>350	6.30
**9**	1 violation: NHorOH>5	Yes	1 violation: TPSA>140	No; 1 violation: TPSA>131.6	No; 2 violations: TPSA>150, H-don>5	0.55	1 alert: quinone_A	1 violation: MW>350	4.96
**10**	2 violations: NorO>10, NHorOH>5	1 violation: WLOGP<-0.4	1 violation: TPSA>140	1 violation: TPSA>131.6	3 violations: TPSA>150, H-acc>10, H-don>5	0.17	1 alert: quinone_A	1 violation: MW>350	5.17
**11**	2 violations: MW>500, NorO>10	2 violations: MW>480, MR>130	1 violation: TPSA>140	1 violation: TPSA>131.6	2 violations: TPSA>150, H-acc>10	0.17	0 alert	2 violations: MW>350, Rotors>7	5.71
**12**	Yes	Yes	Yes	Yes	Yes	0.55	2 alerts: catechol_A, hzone_phenol_B	Yes	2.55
**13**	Yes	Yes	Yes	Yes	Yes	0.56	0 alert	Yes	2.70
**14**	Yes	Yes	Yes	Yes	Yes	0.55	1 alert: catechol_A	Yes	3.50
**15**	2 violations: MW>500, NorO>10	2 violations: MW>480, MR>130	1 violation: TPSA>140	1 violation: TPSA>131.6	2 violations: TPSA>150, H-acc>10	0.17	0 alert	2 violations: MW>350, Rotors>7	5.80
**16**	Yes	Yes	Yes	Yes	Yes	0.55	0 alert	Yes	3.45
**17**	Yes	Yes	Yes	Yes	Yes	0.55	0 alert	Yes	3.41
**18**	Yes	Yes	Yes	Yes	Yes	0.56	0 alert	Yes	2.52
**19**	2 violations: MW>500, MLOGP>4.15	4 violations: MW>480, WLOGP>5.6, MR>130, #atoms>70	Yes	1 violation: WLOGP>5.88	2 violations: MW>600, XLOGP3>5	0.17	0 alert	3 violations: MW>350, Rotors>7, XLOGP3>3.5	5.43
**20**	1 violation: MW>500	2 violations: MW>480, MR>130	1 violation: TPSA>140	1 violation: TPSA>131.6	Yes	0.56	0 alert	1 violation: MW>350	3.30
**21**	2 violations: MW>500, NorO>10	2 violations: MW>480, MR>130	1 violation: TPSA>140	1 violation: TPSA>131.6	2 violations: TPSA>150, H-acc>10	0.17	0 alert	2 violations: MW>350, Rotors>7	5.78
**22**	1 violation: MW>500	3 violations: MW>480, MR>130, #atoms>70	Yes	Yes	1 violation: #rings>7	0.55	0 alert	2 violations: MW>350, XLOGP3>3.5	5.65

**23**	1 violation: MW>500	3 violations: MW>480, MR>130, #atoms>70	Yes	Yes	2 violations: MW>600, XLOGP3>5	0.55	0 alert	3 violations: MW>350, Rotors>7, XLOGP3>3.5	5.46
**24**	1 violation: MW>500	3 violations: MW>480, MR>130, #atoms>70	Yes	Yes	No; 1 violation: MW>600	0.55	0 alert	3 violations: MW>350, Rotors>7, XLOGP3>3.5	5.19
**25**	2 violations: MW>500, MLOGP>4.15	3 violations: MW>480, MR>130, #atoms>70	Yes	Yes	1 violation: XLOGP3>5	0.17	0 alert	2 violations: MW>350, XLOGP3>3.5	5.05
**26**	Yes	2 violations: MW>480, MR>130	Yes	Yes	Yes	0.55	0 alert	1 violation: MW>350	5.41
**27**	1 violation: MLOGP>4.15	1 violation: MR>130	Yes	Yes	Yes	0.55	0 alert	2 violations: MW>350, XLOGP3>3.5	5.17
**28**	Yes	Yes	Yes	Yes	Yes	0.55	0 alert	2 violations: MW>350, XLOGP3>3.5	3.23
**29**	2 violations: NorO>10, NHorOH>5	1 violation: WLOGP<-0.4	1 violation: TPSA>140	1 violation: TPSA>131.6	3 violations: TPSA>150, H-acc>10, H-don>5	0.17	0 alert	1 violation: MW>350	5.20
**30**	Yes	1 violation: MR>130	Yes	Yes	Yes	0.55	0 alert	1 violation: MW>350	5.24

Table S9. The ADMET results of admetSAR server

Compounds	Caco-2	HIA	P-gp I	P-gp S	Carcinogenicity	Ames Mutagenesis	AOT	Dose for AOT (kg/mol)	AD
**1**	No	Yes	No	No	No	Yes	III	2.559	ID
**2**	No	Yes	No	No	No	No	III	2.805	Warning
**3**	No	Yes	No	No	No	Yes	III	3.287	Warning
**4**	No	Yes	No	No	No	No	III	2.243	ID
**5**	No	Yes	No	No	No	Yes	III	2.174	ID
**6**	No	Yes	No	No	No	No	II	2.444	ID
**7**	No	Yes	No	No	No	No	III	2.403	ID
**8**	No	Yes	No	No	No	Yes	III	2.977	Warning
**9**	No	Yes	No	No	No	No	III	2.243	ID
**10**	No	Yes	No	No	No	No	IV	2.419	ID
**11**	No	Yes	Yes	No	No	Yes	III	2.843	ID
**12**	No	Yes	No	No	No	Yes	III	1.723	ID
**13**	No	Yes	No	No	No	No	III	1.681	ID
**14**	No	Yes	No	No	No	Yes	IV	1.972	ID
**15**	No	Yes	Yes	Yes	No	Yes	III	2.876	ID
**16**	Yes	Yes	No	No	No	No	III	2.257	ID
**17**	Yes	Yes	No	No	No	No	III	2.689	ID
**18**	No	Yes	No	No	No	No	III	1.749	ID
**19**	No	Yes	Yes	No	No	Yes	III	2.661	ID
**20**	No	Yes	Yes	No	No	No	III	1.652	ID
**21**	No	Yes	Yes	No	No	Yes	III	2.664	ID
**22**	No	Yes	Yes	Yes	No	Yes	III	2.537	warning
**23**	No	Yes	Yes	Yes	No	No	III	3.19	ID
**24**	No	Yes	Yes	Yes	No	No	III	2.263	ID
**25**	No	Yes	Yes	Yes	No	Yes	III	2.443	ID
**26**	No	Yes	Yes	Yes	No	Yes	III	2.678	ID
**27**	No	Yes	Yes	Yes	No	Yes	III	2.445	ID
**28**	No	Yes	No	No	No	No	III	1.906	ID
**29**	No	Yes	No	No	No	Yes	III	3.205	warning
**30**	No	Yes	Yes	Yes	No	Yes	III	2.237	ID

AD = Applicability domain, AOT = Acute Oral Toxicity, HIA = Human Intestinal Absorption, BBBP = Blood brain barrier permeability, HOB = human oral bioavailability, ID = In Domain, P-gp I = P-glycoprotein inhibitor, P-gp S = P-glycoprotein Substrate

For In domain, Out domain and Warning, please read (http://lmmd.ecust.edu.cn/admetsar2/about/ad). In admetSAR (v2), six physicochemical or topological properties are used to define the applicability domain, which are molecular weight, alogP, number of atoms, number of rings, H-bond acceptors, and H-bond donors. We analysed the distribution of these properties in all the training sets of the predictive models.

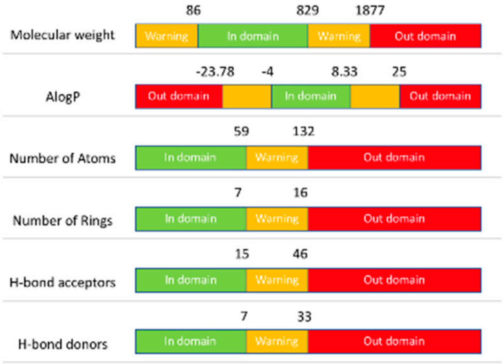

Compounds with the molecular weight higher than 99% or lower than 99% of the training set will be regarded as warning, and if it is higher than the maximum of the training set, it will be tagged as out domain. Similarly, compounds with the alogP higher than 99% or lower than 99% of the training set will be regarded as warning, and if it is higher than the maximum or lower than the minimum of the training set, it will be tagged as out domain. For the other four stistics properties, since their lower bound is 0 and is reasonable, we only tag the overhigh compounds.

Table S10. Interaction Analysis of Selected Hits (1-30)

Compounds	Binding Subsites	Ligand Atom	Receptor Atoms	Bond	Distance (Å)
**1**	S1	O25	O-Phe140	HBD	2.14
	S3	O29	O-His164	HBD	2.07
	S1, S2	O31	SG-Cys145	HBD	3.34
	S1	O22	NE2-His163	HBA	1.58
**2**	S1	O17	O-Glu166	HBD	2.01
	S1, S2	O50	SG-Cys145	HBD	2.1
	S3	O54	SD-Met165	HBD	2.51
**3**	Near S1	O23	O-Leu141	HBD	2.18
	S1	O42	O-Glu166	HBD	2.32
	S1	O22	N-Glu166	HBA	2.46
**4**	S1	O47	O-Glu166	HBD	2.2
	S1	O20	N-Glu166	HBA	3.07
	D3	O49	NE2-Gln192	HBA	3.28
**5**	S1, S2	O22	SG-Cys145	HBD	3.25
	Near S1	O25	O-Leu141	HBD	1.87
**6**	D3	O27	O-Thr190	HBD	2.34
	Near S1	O34	OD1-Asn142	HBD	2.26
	D3	O27	NE2-Gln192	HBA	1.8
**7**	S1, S2	O20	SG-Cys145	HBD	2.46
	S1	O21	OE1-Glu166	HBD	2.39
	D3	O47	O-Thr190	HBD	1.77
	S2	C13	5 ring His41	H-Pi	3.09
	S2	O23	5-ring His41	H-Pi	3.31
**8**	S1, S2	O11	SG-CYS145	HBD	2.83
	Near S1	O46	O-LEU141	HBD	2.15
	S1	O24	N-GLY143	HBA	1.57
	S1	O46	NE2-HIS163	HBA	2.49
	D3	O69	N-THR190	HBA	2.03
	D3	O69	NE2-GLN192	HBA	2.22
	S2	6-ring	CE1-HIS41	H-Pi	3.19
**9**	S1	O23	SG-Ser144	HBD	2.93
	D3	O45	N-Thr190	HBA	2.92
	D3	O45	NE2-Gln192	HBA	2.8
**10**	D3	O49	O-GLN192	HBD	1.57
	D3	O39	NE2-Gln192	HBA	1.9
	S1	O51	NE2-His163	HBA	2.41
	D3	O55	N-Thr190	HBA	2.48
	S2	C13	5-ring His41	H-pi	3.28
**11**	Near S1	O38	OD1-Asn142	HBD	2.12
	D3	O66	O-Arg188	HBD	2.17
	S1	O13	N-Glu166	HBA	3
**12**	D3	O30	O-Arg188	HBD	2.42
	S1	N18	N-Glu166	HBA	2.1
**13**	D3	O28	O-Arg188	HBD	2.34
	S1	O30	O-Glu166	HBD	1.9
	S1	O33	N-Glu166	HBA	1.62

**14**	S1	O28	OG-SER144	HBD	2.85
	S1	O16	N-GLU166	HBA	2.66
**15**	S1	O26	NE2-HIS163	HBA	3.25
	D3	O47	N-THR190	HBA	3.24
	D3	O47	NE2-GLN192	HBA	2.82
**16**	S1	O40	N-SER144	HBA	2.98
	S1	O40	OG-SER144	HBA	2.63
	S1	O41	N-GLY143	HBA	2.12
**17**	D3	O22	N-Thr190	HBA	2.52
	D3	O22	NE2-Gln192	HBA	2.48
**18**	D3	O29	O-Gln192	HBD	2.05
	S1	O14	N-Glu166	HBA	1.60
	S1	5-ring	CB-Glu166	Π-H	3.07
**19**	S1	N-10	N-GLU166	HBA	2.31
	S1	6-ring	N-GLU166	Π-H	3.64
**20**	S1, S2	O13	N-CYS145	HBA	3.00
	S1	O52	N-SER144	HBA	2.638
	S1	O52	OG-SER144	HBA	1.96
**21**	S1, S2	O62	SG-CYS145	HBD	2.38
**22**	S3	N57	O-HIS164	HBD	2.80
**23**	S1	O45	N-GLU166	HBA	2.72
	S3	6-ring	CA-MET165	Π-H	2.76
**24**	S1	N41	N-GLU166	HBA	2.37
**25**	S1	N23	O-GLU166	HBA	1.71
	S1	N58	NE2-HIS163	HBA	2.54
**26**	S1	O8	N-GLY143	HBA	2.25
	S1	N37	N-GLY143	HBA	3.24
**27**	S1	N18	N-GLU166	HBA	2.76
	S1	N22	N-GLU166	HBA	3.04
**28**	S1	N15	N-Ser144	HBA	2.56
	S1	N15	OG-Ser144	HBA	2.32
**29**	S1	O38	O-GLU166	HBA	1.77
	S1	O53	O-PHE140	HBD	2.23
	S1	O23	N-GLU166	HBA	2.55
	S1	O33	N-GLU166	HBA	2.90
	S2	6-ring	5-ring-HIS41	Pi-pi	3.93
**30**	S1	O9	N-GLY143	HBA	1.87

HBA = Hydrogen bond acceptor, HBD = Hydrogen bond donor, D3 = Domain 3

Table S11. Protein-ligand complexes used in Re-docking and Cross-docking

S. No.	PDB ID	Ligands		Interacting Residues	Resolution (Å)	Reference
1	6M2N	5,6,7-trihydroxy-2-phenyl-4H-chromen-4-one (**3WL, Baicalein, IC50 = 0.94 µM**)*	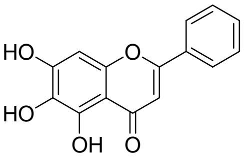	Gly143, Glu166	2.20	Su, H-x, et al. Anti-SARS-CoV-2 activities in vitro of Shuanghuanglian preparations and bioactive ingredients, Acta Pharmacologica Sinica, 41, 1167–1177 (2020)
2	6LU7	N-[(5-methylisoxazol-3-yl)carbonyl]alanyl-l-valyl-n~1~-((1r,2z)-4-(benzyloxy)-4-oxo-1-{[(3r)-2-oxopyrrolidin-3-yl]methyl}but-2-enyl)-l-leucinamide (**N3, IC_{50} = 0.4** and **1.0 µM**)*	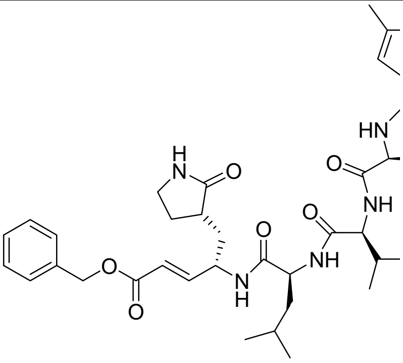	Phe140. Gly143, His163, Glu166, Gln189, Thr190	2.16	Jin, Z., et al., Structure of M^{pro} from COVID-19 virus and discovery of its inhibitors. Nature, 582, 289–293 (2020)
3	6W63	N-(4-tert-butylphenyl)-N-[(1R)-2-(cyclohexylamino)-2-oxo-1-(pyridin-3-yl)ethyl]-1H-imidazole-4-carboxamide **(X77, Kd = 0.057 µM)***	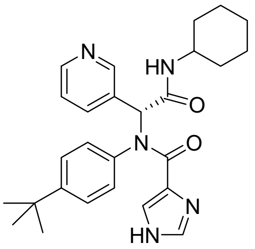	Gly143, His163, Glu166	2.10	Activity is taken from https://www.medchemexpress.com/x77.html
4	6W79	N-(4-tert-butylphenyl)-N-[(1R)-2-(cyclohexylamino)-2-oxo-1-(pyridin-3-yl)ethyl]-1H-imidazole-4-carboxamide **(X77, Kd = 0.057 µM)**	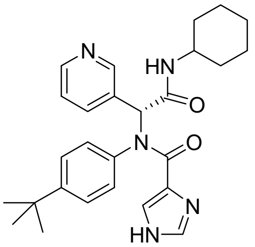	Gly143, His163, Glu166	1.46	https://www.medchemexpress.com/x77.html
5	6WNP	Boceprevir (**IC_{50} = 4.13 µM**)	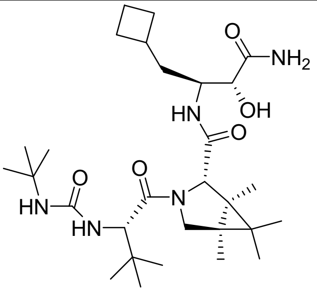	His41, Gly143, Cys145, Glu166	1.44	Ma, C. et al., Boceprevir, GC-376, and calpain inhibitors II, XII inhibit SARS-CoV-2 viral replication by targeting the viral main protease, Cell Research, 30, 678–692 (2020)

6	6WTK	N~2~-[(benzyloxy)carbonyl]-N-{(2S)-1-hydroxy-3-[(3S)-2-oxopyrrolidin-3-yl]propan-2-yl}-L-leucinamide (**UED, IC_{50} = 0.40 µM**)	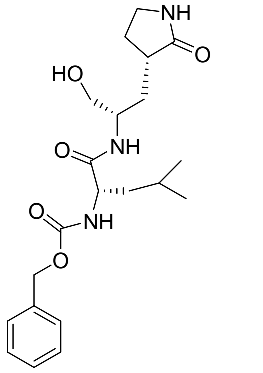	Phe140, Gly143, Cys145, His163, His164, Glu166, Gln189	2.00	Vuong, W et al., Feline coronavirus drug inhibits the main protease of SARS-CoV-2 and blocks virus replication, Nature Communications,11, 1-8 (2020)
7	6Y2F	~{tert}-butyl ~{N}-[1-[(2~{S})-3-cyclopropyl-1-oxidanylidene-1-[[(2~{S},3~{R})-3-oxidanyl-4-oxidanylidene-1-[(3~{S})-2-oxidanylidenepyrrolidin-3-yl]-4-[(phenylmethyl)amino]butan-2-yl]amino]propan-2-yl]-2-oxidanylidene-pyridin-3-yl]carbamate (**O6K, 13b, IC_{50} = 0.67 µM**)	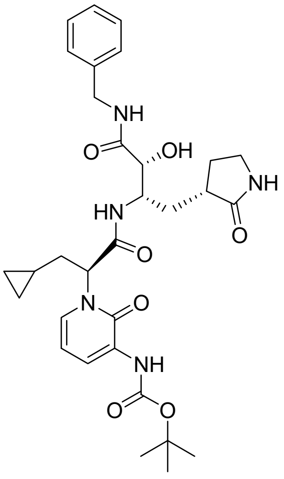	His41, Phe140, Gly143, Cys145, His163, His164, Glu166	1.95	Zhang, L. et al., Crystal structure of SARS-CoV-2 main protease provides a basis for design of improved α-ketoamide inhibitors, Science, 24, 368, 6489, 409-412 (2020)
8	6ZRT	Telaprevir ((1S,3ar,6as)-2-[(2S)-2-({(2S)-2-cyclohexyl-2-[(pyrazin-2-ylcarbonyl)amino]acetyl}amino)-3,3-dimethylbutanoyl]-N-[(2R,3S)-1-(cyclopropylamino)-2-hydroxy-1-oxohexan-3-yl]octahydrocyclopenta[c]pyrrole-1-carboxamide) (**IC_{50} = 55.72 µM**)	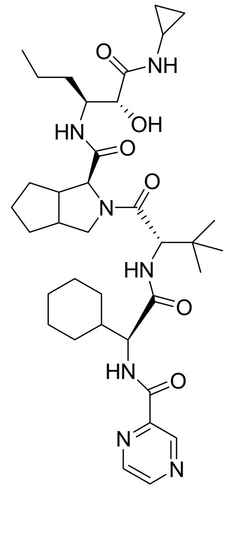	His41, Gly143, Cys145, His164, Glu166, Gln189	2.10	Oerlemans, R., et al. Repurposing the HCV NS3-4A protease drug boceprevir as COVID-19 therapeutics, RSC Med Chem 12, 370-379 (2020)

9	7BQY	N-[(5-methylisoxazol-3-yl) carbonyl]alanyl-l-valyl-n~1~-((1r,2z)-4-(benzyloxy)-4-oxo-1-{[(3r)-2-oxopyrrolidin-3-yl]methyl}but-2-enyl)-l-leucinamide (**N3, IC_{50} = 0.4** and **1.0 µM**)	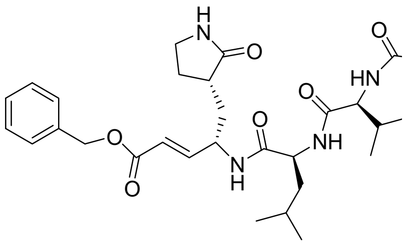	Phe140, Gly143, Cys145, His163, His164, Glu166, Gln189, Thr190	1.70	Jin, Z., et al., Structure of M^{pro} from COVID-19 virus and discovery of its inhibitors. Nature, 582, 289–293 (2020)
10	7D1M (7BRR)	(1S,2S)-2-({N-[(benzyloxy)carbonyl]-L-leucyl}amino)-1-hydroxy-3-[(3S)-2-oxopyrrolidin-3-yl]propane-1-sulfonic acid (**GC376, IC_{50} = 0.15 µM**)	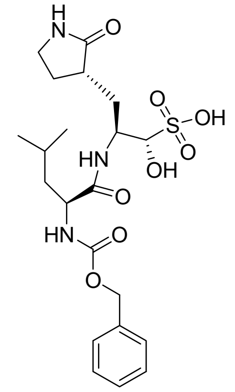	Phe140, Cys145, His163, Glu166, Gln189	1.40	Fu, L. et al., Both Boceprevir and GC376 efficaciously inhibit SARS-CoV-2 by targeting its main protease, Nat Commun, 11, 4417-4417 (2020)
11	7JU7	Masitinib (**IC_{50} = 2.5 and 35 µM**)	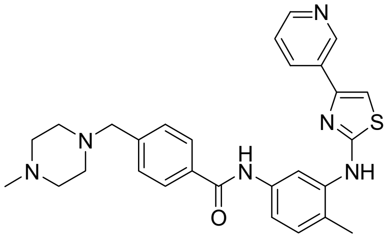	His163, His164	1.60	Drayman, N., et al., Drug repurposing screen identifies masitinib as a 3CLpro inhibitor that blocks replication of SARS-CoV-2 in vitro, bioRxiv, doi: https://doi.org/10.1101/2020.08.31.274639, 2020
12	5R80	Methyl 4-sulfamoylbenzoate	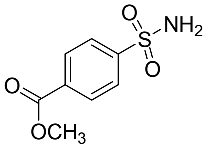	Glu166	1.93	Douangamath, A., et al. Crystallographic and electrophilic fragment screening of the SARS-CoV-2 main protease, Nat Commun 11: 5047-5047 (2020)
13	5R81	1-methyl-3,4-dihydro-2~{H}-quinoline-7-sulfonamide	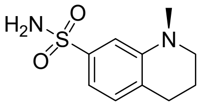	---	1.95	Douangamath, A., et al. Crystallographic and electrophilic fragment screening of the SARS-CoV-2 main protease, Nat Commun 11: 5047-5047 (2020)
14	5R82	6-(ethylamino)pyridine-3-carbonitrile	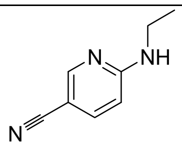	Gln189	1.31	Douangamath, A., et al. Crystallographic and electrophilic fragment screening of the SARS-CoV-2 main protease, Nat Commun 11: 5047-5047 (2020)

15	5R83	N-phenyl-N'-pyridin-3-ylurea	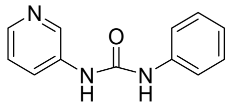	His163, Glu166	1.58	Douangamath, A., et al. Crystallographic and electrophilic fragment screening of the SARS-CoV-2 main protease, Nat Commun 11: 5047-5047 (2020)
16	5R84	2-cyclohexyl-~{N}-pyridin-3-yl-ethanamide	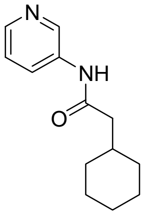	His163, Glu166	1.83	Douangamath, A., et al. Crystallographic and electrophilic fragment screening of the SARS-CoV-2 main protease, Nat Commun 11: 5047-5047 (2020)
17	5R7Y	N-(2-phenylethyl)methanesulfonamide	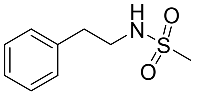	---	1.65	Douangamath, A., et al. Crystallographic and electrophilic fragment screening of the SARS-CoV-2 main protease, Nat Commun 11: 5047-5047 (2020)
18	5R7Z	~{N}-[2-(5-fluoranyl-1~{H}-indol-3-yl)ethyl]ethanamide	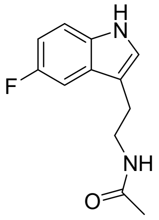	His41, Glu166	1.59	Douangamath, A., et al. Crystallographic and electrophilic fragment screening of the SARS-CoV-2 main protease, Nat Commun 11: 5047-5047 (2020)
19	5RE4	N-(4-methylpyridin-3-yl)acetamide	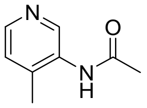	His163, Glu166	1.88	Douangamath, A., et al. Crystallographic and electrophilic fragment screening of the SARS-CoV-2 main protease, Nat Commun 11: 5047-5047 (2020)
20	5RE9	2-(4-methylphenoxy)-1-(4-methylpiperazin-4-ium-1-yl)ethanone	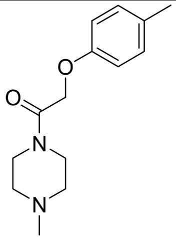	---	1.72	Douangamath, A., et al. Crystallographic and electrophilic fragment screening of the SARS-CoV-2 main protease, Nat Commun 11: 5047-5047 (2020)

Other M^{pro} Inhibitors selected from Publications

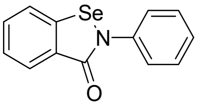 **Ebselen** (IC_{50} = 0.67 μM) Amporndanai, K. et al., Inhibition mechanism of SARS-CoV-2 main protease by ebselen and its derivatives, Nature Communications, 12, (2021)	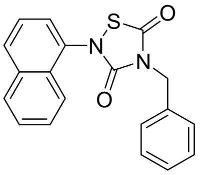 **Tideglusib** (IC_{50} = 1.55 μM)
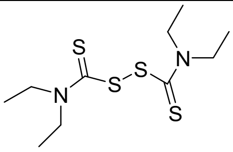	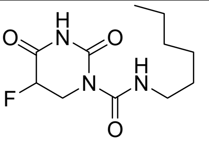

**Disulfiram** (IC_{50} = 9.35 μM)	**Carmofur** (IC_{50} = 1.82 μM)
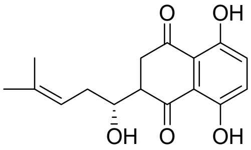 **Shikonin** (IC_{50} = 15.75 μM)	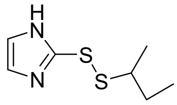 **PX-12** (IC_{50} = 21.39 μM)

Fifteen known inhibitors of M^{pro} including N3 (ligand complexed in 6LU7), Baicalein, X77, Boceprevir, UED, O6K, Telaprevir, GC376, Masitinib, Ebselen, Tideglusib, Disulfiram, Carmofur, Shikonin, and PX-12 (Selected from Literature: Nitsche, C.; Ullrich, S. The SARS-CoV-2 main protease as drug target. *Bioorg Med Chem Lett*. **2020**, *30*, 127377).
